# Supplementary material for: Enhanced PeriOperative Care and Health protection programme for the prevention of surgical site infections after elective abdominal surgery (EPOCH): study protocol of a randomised controlled, multicentre, superiority trial
Source: BMJ Open. 2020 May 25;10(5):e038196. doi: 10.1136/bmjopen-2020-038196 (PMC7252990; doi:10.1136/bmjopen-2020-038196)
Supplement: Supplementary data [file bmjopen-2020-038196supp001.pdf]

Appendix 1. Additional measurements

Background
Studies have shown a negative correlation between tissue oxygen tension and SSI.[1-4] Conversely, improving tissue oxygen tension has shown reduce the risk of SSI.[5] Several interventions, thought to improve tissue oxygen tension, including hyperoxygenation, active warming and goal directed fluid therapy, have shown to help reduce the risk of SSI.[5-7] However, the evidence for these interventions is based on clinical outcomes and the precise mechanism of effect is not entirely clear. In vitro experiments have indicated that optimized oxygenation may enhance immunologic response,[8] but only one study on these interventions actually measured oxygen tension as a process measure.[5] The study, found a marked increase in oxygen tension and over 50% reduction in SSI.[5] Various investigators have tried to reproduce these results with varying success.[9] Since the early positive results, much has changed in perioperative care with considerable implications for cardiovascular and respiratory physiology. Open procedures with aggressive hydration regimens have made room for laparoscopic surgery with enhanced recovery programs and restricted fluid regimens. These changes could modify the effect on oxygen tension and SSI. The interventions thought to improve oxygen tension, Hyperoxygenation, active warming, and goal directed fluid therapy, are all components of the EPOCH bundle. This poses an extraordinary opportunity to investigate the effect of these interventions combined on tissue oxygen tension and immunologic response in the current era. Further understanding of these underlying mechanisms may facilitate optimization if the interventions prove effective, or understanding if they do not. Her we aim to study the effect of the EPOCH interventions on tissue oxygen tension and immunologic response.

Population
Patients enrolled in the Amsterdam UMC, location AMC who did not use immunosuppressants in the previous 90 days (e.g. glucocorticoids, cytostatic agents, antibodies, drugs acting on immunophilins, biologicals) will be approached for participation in additional measurements. This includes subcutaneous tissue oxygen tension measurement, mitochondrial tissue oxygen tension measurement and measurement of immunologic effects. As immunosuppressants blunt immunologic effects, potential differences, if any, will not be detectable in these patients. Therefore, patients using immunosuppressants are excluded for these additional measurements

Considering the substantial cost involved a small subset of the larger population will be measured based on a separate sample size calculation. Previous studies have shown the standard deviation in mean subcutaneous tissue oxygen tension is approximately 43 mm Hg.[5] Based on an alpha of 0.05, a beta of 0.2 (power of 0.8) and standard deviation in mean subcutaneous tissue oxygen tension of 43 mm Hg, 12 participants per arm are needed to show the previously reported 50 mmHg difference in subcutaneous tissue oxygen tension. Because the EPOCH study includes both laparoscopic and open surgery, and the two approaches have meaningful differences in hemodynamics and hemodynamic management, 24 consecutive eligible patients undergoing laparoscopic surgery, and 24 consecutive eligible patients undergoing open surgery will be enrolled until the final sample size is obtained for each. For the measurement of mitochondrial oxygen tension and immunologic effects, no reliable data is available to conduct a sample size estimation. Therefore, both will be measured as a pilot study investigating the 48 participants undergoing subcutaneous tissue oxygen tension measurement. Analysis of these pilot data will inform the need for further data collection.

Outcomes measures
- The difference in mean perioperative subcutaneous PtO_2 (mmHg) at the upper arm between the two treatment arms as measured by the Licox (Integra LifeSciences Corp, Plainsboro, New Jersey).

- The difference in mean perioperative mitochondrial PtO_2 (mmHg) in the epidermis, measured parasternal, between the two treatment arms as measured by the COMET (Photonics Healthcare B.V.).
- The difference in perioperative immune response between the two treatment arms as measured by neutrophil intracellular ROS generation, phagocytosis assays, plasma cytokine concentrations, plasma malondialdehyde concentration, leukocytic HLA-DRA mRNA expression, and ex vivo whole blood stimulation in blood samples withdrawn at five pre-specified timepoints.

Techniques

For subcutaneous tissue oxygen tension measurement, The Licox (Integra LifeSciences Corp, Plainsboro, New Jersey) system will be used. Licox continuously calculates tissue partial tension of oxygen through a Clark type polarographic oxygen electrode in a micro-catheter for oxygen measurements positioned in the tip of a flexible probe. The probe, with an outer diameter of 1.0mm will be inserted in the subcutaneous tissue with a 19-gauge venflon needle after local anaesthesia, and shortly before induction of general anaesthesia. Probes and instruments required for placement are sterile, single use and will be positioned maintaining sterile technique. The probe will be removed before discharge from the recovery room. For mitochondrial tissue oxygen measurement, the Cellular Oxygen METabolism (COMET) device (Photonics Healthcare B.V. (PH) will be used. The COMET is a non-invasive bedside monitoring system. After application of a dermatologic agent aminolevulinic acid (ALA) patch to the patient skin on the night before surgery, a sensor is applied to the skin of the thorax. The skin sensor creates a diverging excitation pulse allowing a superficial optical measurement of the oxygen in the mitochondria of the cells in the epidermis in a circular area of about 5 mm^2. Fluorescence light from the tissue is collected and transmitted via the detection fibre towards the detector system.[10-12] Blood samples (EDTA anticoagulated, LH anticoagulated, PAXgene) will be withdrawn at 5 pre-specified time points in a limited subset of the population (24 patients); along with routine preoperative blood sampling, after induction of anaesthesia but prior to incision, at the end of surgery, one hour postoperatively and at the first postoperative outpatient visit. Neutrophil ROS generation will be quantified using 2',7' dichlorofluoresciein diacetate (DCFH-DA; Sigma, St Louis, MO, USA), as described by Perazzio et al.[13] Neutrophil phagocytosis will be measured using the pHrodo Red *E. coli* BioParticles Phagocytosis Kit for Flow Cytometry according to the manufacturer's instructions (LifeTechnologies, Bleiswijk, the Netherlands). EDTA-anticoagulated blood will be centrifuged immediately after withdrawal (2000 g, 10 minutes, 4 °C). Plasma will be stored at − 80 °C until analysis. Concentrations of cytokines (including but not limited to TNFα, IL-6, IL-8, and IL-10) will be analysed batch-wise using a Luminex assay (detection range 3.2-10000 pg/mL) according to the manufacturer's instructions (Milliplex, Millipore, Billerica, MA, USA). RNA will be isolated for HLA-DRA mRNA expression using the Paxgene Blood RNA kit (Qiagen, Valencia, CA, USA) and transcribed into cDNA using the iScript cDNA Synthesis kit (Bio-rad, Hercules, CA, USA). qPCR analysis was performed using TaqMan gene expression assays (Life Technologies, Paisley, UK) for the reference gene peptidylpropylisomerase B (PPIB) (#Hs00168719_m1) and HLA-DRA (#Hs00219575_m1) on a CFX96 Real-Time PCR Detection System (Bio-Rad, Hercules, CA, USA). Lipid peroxidation will be determined by measuring levels of malondialdehyde (MDA) in EDTA plasma using the Thiobarbituric Acid Reactive Substances (TBARS) Parameter Assay Kit (R&D Systems, Abingdon, UK) according to the manufacturer's instructions. Leukocyte cytokine production capacity will be assessed by ex vivo stimulation of whole blood with Lipopolysacharide (LPS) ex vivo, as described by Kox et al.[14] Blood gas parameters will be analysed in LH anticoagulated blood using the Rapidlab 1200 Series (Bayer Healthcare LLC, Berlin, Germany) according to manufacturer's instructions

References

1. Hunt TK. Surgical wound infections: an overview. Am J Med 1981;70:712-8.
2. Hopf HW, Hunt TK, West JM, et al. Wound tissue oxygen tension predicts the risk of wound infection in surgical patients. Arch Surg 1997;132:997-1004; discussion 5.
3. Govinda R, Kasuya Y, Bala E, et al. Early postoperative subcutaneous tissue oxygen predicts surgical site infection. Anesth Analg 2010;111:946-52.
4. Ives CL, Harrison DK, Stansby GS. Tissue oxygen saturation, measured by near-infrared spectroscopy, and its relationship to surgical-site infections. Br J Surg 2007;94:87-91.
5. Greif R, Akça O, Horn EP, Kurz A, Sessler DI. Supplemental perioperative oxygen to reduce the incidence of surgical-wound infection. N Engl J Med 2000;342:161-7.
6. Kurz A, Sessler DI, Lenhardt R. Perioperative normothermia to reduce the incidence of surgical-wound infection and shorten hospitalization. Study of Wound Infection and Temperature Group. N Engl J Med 1996;334:1209-15.
7. Dalfino L, Giglio MT, Puntillo F, Marucci M, Brienza N. Haemodynamic goal-directed therapy and postoperative infections: earlier is better. A systematic review and meta-analysis. Crit Care 2011;15:R154.
8. Qadan M, Battista C, Gardner SA, Anderson G, Akca O, Polk HC, Jr. Oxygen and surgical site infection: a study of underlying immunologic mechanisms. Anesthesiology 2010;113:369-77.
9. de Jonge S, Egger M, Latif A, et al. Effectiveness of 80% vs 30-35% fraction of inspired oxygen in patients undergoing surgery: an updated systematic review and meta-analysis. Br J Anaesth 2019;122:325-34.
10. Mik EG, Stap J, Sinaasappel M, et al. Mitochondrial PO2 measured by delayed fluorescence of endogenous protoporphyrin IX. Nat Methods 2006;3:939-45.
11. Mik EG, Johannes T, Zuurbier CJ, et al. In vivo mitochondrial oxygen tension measured by a delayed fluorescence lifetime technique. Biophys J 2008;95:3977-90.
12. Harms FA, de Boon WM, Balestra GM, et al. Oxygen-dependent delayed fluorescence measured in skin after topical application of 5-aminolevulinic acid. J Biophotonics 2011;4:731-9.
13. Perazzio SF, Salomao R, Silva NP, Andrade LE. Increased neutrophil oxidative burst metabolism in systemic lupus erythematosus. Lupus 2012;21:1543-51.
14. Kox M, Vrouwenvelder MQ, Pompe JC, van der Hoeven JG, Pickkers P, Hoedemaekers CW. The effects of brain injury on heart rate variability and the innate immune response in critically ill patients. J Neurotrauma 2012;29:747-55.
