# Supplementary material for: Enhanced PeriOperative Care and Health protection programme for the prevention of surgical site infections after elective abdominal surgery (EPOCH): study protocol of a randomised controlled, multicentre, superiority trial
Source: BMJ Open. 2020 May 25;10(5):e038196. doi: 10.1136/bmjopen-2020-038196 (PMC7252990; doi:10.1136/bmjopen-2020-038196)
Supplement: Supplementary data [file bmjopen-2020-038196supp002.pdf]

Effect of an Enhanced PeriOperative Care and Health Program

Academisch Medisch Centrum
Universiteit van Amsterdam

Patiënt informatie brief
Studie naar het effect van een geoptimaliseerd zorgprogramma rondom operaties op het voorkomen van wondinfecties.

Geachte mevrouw / mijnheer,
U bent door uw behandelend arts gevraagd om mee te doen aan een klinisch medisch wetenschappelijk onderzoek. In deze brief kunt u de informatie over dit onderzoek nog eens rustig nalezen, zodat u een weloverwogen beslissing kunt nemen. Wanneer u dat wilt, betrek dan ook uw eventuele partner of familie in de afweging. Voor algemene informatie verwijzen wij u naar de brochure "Medisch-wetenschappelijk onderzoek; Algemene informatie voor de proefpersoon". Wanneer u deze nog niet in bezit hebt, vraag dan uw arts ernaar.

Achtergrond
Wondinfecties na operaties behoren tot de meest voorkomende ziekenhuisinfecties. In Nederland ontwikkelt ongeveer 9% van alle patiënten die een buikoperatie ondergaat een wondinfectie. Wondinfecties variëren in ernst van een oppervlakkige ontsteking tot abcessen of bloedvergiftiging en geven altijd een verminderd operatie resultaat. Vaak moeten patiënten met een wondinfectie langer in het ziekenhuis blijven, opnieuw opgenomen worden of soms zelfs opnieuw geopereerd worden. Hierdoor zorgen wondinfecties voor onnodig veel pijn, ongemak, extra ziektelast en een aanzienlijke toename in de kosten. Wondinfecties zijn niet altijd te vermijden maar er zijn sterke aanwijzingen dat we onze huidige zorg rond operaties kunnen verbeteren om het risico naar beneden te brengen.

Doel van dit onderzoek
In Nederland is de standaard zorg om wondinfecties te voorkomen nu beschreven in de POWI (Post Operatieve Wond Infectie) bundel. Dit is een bundel van vier maatregelen die het risico op wondinfecties moeten verkleinen. Hierin staat dat patiënten op tijd antibiotica moeten krijgen, de deur tijdens de operatie niet te vaak open mag, u niet voor de operatie geschoren mag worden en dat u na de operatie niet te ver afgekoeld mag zijn. Er zijn sterke aanwijzingen dat andere, relatief eenvoudige, maatregelen tijdens de operatie het risico op wondinfecties terug kunnen brengen. Toch worden ze lang nog niet altijd toegepast. Nog niet alle artsen zijn overtuigd van het effect. Veel van deze maatregelen hebben effect op elkaar. Met deze studie willen we onderzoeken of de maatregelen als bundel elkaar versterken en een sterke vermindering geven op het aantal wondinfecties.

Deze bundel bestaat uit:

- Actieve verwarming rondom de operatie
  - Voor, tijdens en na de operatie krijgt de patiënt verwarmde dekens om te voorkomen dat hij of zij afkoelt

- Extra zuurstof tijdens de operatie
  - Tijdens de operatie geven we de patiënt lucht waar wat extra zuurstof in zit (80% i.p.v. 30%)

de Jonge SW, *et al. BMJ Open* 2020; 10:e038196. doi: 10.1136/bmjopen-2020-038196

EPOCH Effect of an Enhanced PeriOperative Care and Health Program

amc Academisch Medisch Centrum Universiteit van Amsterdam

- Bloedsuiker controle tijdens en kort na de operatie
    - Tijdens de operatie houden we het bloedsuikergehalte extra goed in de gaten en geven we de patiënt bloedsuiker verlagende medicijnen als dat nodig is. Na de operatie controleren we nog twee keer of het bloedsuikergehalte in orde is.

- Regulering van uw bloedsomloop op basis van een doelgericht protocol
    - We volgen een duidelijk doelgericht protocol waarin staat wanneer, en hoeveel vocht we toedienen tijdens de operatie om de bloedsomloop optimaal te houden.

- Gestandaardiseerde behandeling van de wond
    - Het wondgebied wordt volgens een gestandaardiseerde manier behandeld: Poetsen met alcohol en wondspoeling met jodium voor het dichtmaken van de wond.

Al deze maatregelen vinden voor het grootste deel plaats terwijl u onder narcose bent.

Daarnaast willen we onderzoeken welke rol patiënten kunnen spelen in het vaststellen van wondinfecties aan de hand van de resultaten van dit onderzoek.

Hoe wordt het onderzoek uitgevoerd?
Alle patiënten die een buikoperatie ondergaan zullen worden gevraagd mee te doen aan dit onderzoek. Om uit te zoeken of onze EPOCH bundel van maatregelen beter werkt dan de standaard zorg worden patiënten in twee groepen verdeeld. De eerste groep patiënten krijgt de standaard zorg zoals de dokters dat nu tijdens de operatie gewend zijn. De tweede groep krijgt de standaard zorg zoals de eerste groep, maar aangevuld met de EPOCH bundel aan extra maatregelen rondom de operatie. Verder gebeurt alles rondom de operatie zoals u dat met uw arts besproken heeft.
Als u besluit mee te doen met het onderzoek, wordt u ingedeeld in een van de twee groepen. Om de verdeling zo eerlijk mogelijk te houden, word deze bepaald door loting. Per dag wordt geloot in welke operatiekamers de standaard zorg wordt toegepast en in welke de EPOCH bundel daaraan toe wordt gevoegd.

Wat is er anders dan de reguliere behandeling die u krijgt?
Afhankelijk van de groep waarin u wordt geloot krijgt u de standaard zorg rondom operaties zoals we die nu gewend zijn, of diezelfde zorg aangevuld met de bundel aan extra maatregelen uit onze studie. Daarnaast wordt u gevraagd om een digitale foto te maken van de wond – indien u daar de technische mogelijkheden toe heeft – na 10 en na 30 dagen, en wanneer u in verband met zorgen om de wond contact hebt gehad met een arts. Met deze foto's kunnen we het proces van wondgenezing goed vervolgen. Op 30, 60 en 90 dagen na de behandeling wordt u gevraagd een aantal vragenlijsten in te vullen. Dit kan op uw smart Phone via de website of op papier. Deze vragenlijsten hebben betrekking op uw wondgenezing, klachten en welbevinden. Dit zal afhankelijk van de vragenlijst ongeveer 10 of 15 minuten per keer in beslag nemen. Bij de laatste vragenlijsten zitten twee extra vragenlijsten die nog eens 30 minuten in beslag nemen. In totaal 65 minuten verspreid over drie maanden. Hiervoor kunt u benaderd worden door de arts-onderzoeker, die hierdoor inzage heeft in uw persoonsgegevens.

de Jonge SW, *et al. BMJ Open* 2020; 10:e038196. doi: 10.1136/bmjopen-2020-038196

EPO2CH
Effect of an Enhanced PeriOperative Care and Health Program

amc
Academisch Medisch Centrum
Universiteit van Amsterdam

Wat zijn mogelijke voor- en nadelen van deelname aan dit onderzoek?
Bij deelname aan dit onderzoek wordt u geloot tussen de standaard zorg, of de geoptimaliseerde zorg (EPOCH bundel). U heeft dus een 50/50 kans. Wij denken dat de geoptimaliseerde behandeling het risico op wondinfecties zou kunnen verlagen, maar dat moeten we nog bewijzen. Er is dus bij aanvang van dit onderzoek nog geen bewezen voordeel voor deelname aan dit onderzoek. Behalve 3 vingerprikjes voor de bloed glucose controle zijn van de extra maatregelen geen nadelen bekend. Wat wel een mogelijk nadeel van de studie is, is dat het invullen van de vragenlijsten wat extra tijd van u vergt. Verspreid over drie maanden is dat ongeveer een uur.

Wordt u geïnformeerd als er tussentijds voor u relevante informatie over de studie bekend wordt?
Het onderzoek zal zo nauwkeurig mogelijk en volgens een vooraf opgesteld plan verlopen. Maar de situatie kan veranderen. Als dat zo is, bespreken we dat direct met u. U beslist dan zelf of u met het onderzoek wilt stoppen of doorgaan.

Vrijwilligheid van deelname
Uw medewerking aan dit onderzoek is vrijwillig. Als u toestemming geeft om aan dit onderzoek mee te doen, heeft u te allen tijde de vrijheid om op die beslissing terug te komen. U hoeft hiervoor geen reden op te geven. Ook uw behandelend arts kan uw deelname aan het onderzoek stopzetten als deze vindt dat dit ten aanzien van uw gezondheid beter is. Uw arts bespreekt dit dan met u. Een beslissing om uw medewerking te beëindigen zal geen nadelige gevolgen hebben op de verdere behandeling en geen invloed hebben op de zorg en aandacht waarop u in ons ziekenhuis recht hebt.

Wanneer u afziet van deelname?
Als u geen toestemming geeft voor deelname aan het onderzoek, ondergaat u de standaard zorg rondom operaties zoals we dat nu gewend zijn conform de huidige richtlijnen.

Gebruik en bewaren van uw gegevens
Voor dit onderzoek worden uw persoonsgegevens verzameld, gebruikt en bewaard. Het gaat om gegevens zoals uw naam, adres, geboortedatum en om gegevens over uw gezondheid. Het verzamelen, gebruiken en bewaren van uw gegevens is nodig om de vragen die in dit onderzoek worden gesteld te kunnen beantwoorden en de resultaten te kunnen publiceren. Wij vragen voor het gebruik van uw gegevens uw toestemming.

Vertrouwelijkheid van uw gegevens
Om uw privacy te beschermen krijgen uw gegevens een code. Uw naam en andere gegevens die u direct kunnen identificeren worden daarbij weggelaten. Alleen met de sleutel van de code zijn gegevens tot u te herleiden. De sleutel van de code blijft veilig opgeborgen in de lokale onderzoeksinstelling. De gegevens bevatten alleen de code, maar niet uw naam of andere gegevens waarmee u kunt worden geïdentificeerd. Ook in rapporten en publicaties over het onderzoek zijn de gegevens niet tot u te herleiden.

de Jonge SW, *et al. BMJ Open* 2020; 10:e038196. doi: 10.1136/bmjopen-2020-038196

Toegang tot uw gegevens voor controle
Sommige personen kunnen op de onderzoekslocatie toegang krijgen tot al uw gegevens. Ook tot de gegevens zonder code. Dit is nodig om te kunnen controleren of het onderzoek goed en betrouwbaar is uitgevoerd. Personen die ter controle inzage krijgen in uw gegevens zijn: de commissie die de veiligheid van het onderzoek in de gaten houdt, een controleur die voor het AMC als opdrachtgever werkt, nationale toezichthoudende autoriteiten, bijvoorbeeld, de Inspectie Gezondheidszorg. Zij houden uw gegevens geheim. Wij vragen u voor deze inzage toestemming te geven.

Bewaartermijn gegevens
Uw gegevens moeten 15 jaar worden bewaard op de onderzoekslocatie en 15 jaar bij de opdrachtgever (het AMC).
Het wordt bewaard om daarop in de loop van dit onderzoek nog nieuwe bepalingen te kunnen doen die te maken hebben met dit onderzoek.

Bewaren en gebruik van gegevens voor ander onderzoek
Uw gegevens kunnen na afloop van dit onderzoek ook nog van belang zijn voor ander wetenschappelijk onderzoek op het gebied van wondinfecties. Daarvoor zullen uw gegevens 15 jaar worden bewaard. U kunt op het toestemmingsformulier aangeven of u hier wel of niet mee instemt. Indien u hier niet mee instemt, kunt u gewoon deelnemen aan het huidige onderzoek.

Intrekken toestemming
U kunt uw toestemming voor gebruik van uw persoonsgegevens altijd weer intrekken. Dit geldt voor dit onderzoek en ook voor het bewaren en het gebruik voor het toekomstige onderzoek. De onderzoeksgegevens die zijn verzameld tot het moment dat u uw toestemming intrekt worden nog wel gebruikt in het onderzoek.

Meer informatie over uw rechten bij verwerking van gegevens
Voor algemene informatie over uw rechten bij verwerking van uw persoonsgegevens kunt u de website van de Autoriteit Persoonsgegevens raadplegen.
Bij vragen over uw rechten kunt u contact opnemen met de verantwoordelijke voor de verwerking van uw persoonsgegevens. Voor dit onderzoek is dat het AMC, zie contactgegevens verderop.
Bij vragen of klachten over de verwerking van uw persoonsgegevens raden we u aan eerst contact op te nemen met de onderzoek locatie. U kunt ook contact opnemen met de Functionaris voor de Gegevensbescherming van het AMC (zie contactgegevens) of de Autoriteit Persoonsgegevens.

Registratie van het onderzoek
Informatie over dit onderzoek is ook opgenomen in een overzicht van medisch-wetenschappelijke onderzoeken namelijk (www.trialregister.nl). Daarin zijn geen gegevens opgenomen die naar u herleidbaar zijn. Na het onderzoek kan de website een samenvatting van de resultaten van dit onderzoek tonen. U vindt dit onderzoek onder nummer NTR5694.

de Jonge SW, *et al. BMJ Open* 2020; 10:e038196. doi: 10.1136/bmjopen-2020-038196

Bent u verzekerd wanneer u aan het onderzoek meedoet?
Aangezien aan deelname aan deze studie geen risico's verbonden zijn, heeft de Medisch Ethische Toetsingscommissie ontheffing verleend van de verplichting om voor de deelnemers een speciale schadeverzekering af te sluiten

Is er een vergoeding wanneer u besluit aan dit onderzoek mee te doen?
Er worden geen vergoedingen gedaan voor het meedoen aan dit onderzoek of voor uw reiskosten.

Hoe te handelen bij klachten?
Als u klachten heeft over het onderzoek, kunt u dit melden aan de onderzoeker. Wilt u dit liever niet, dan kunt u contact opnemen met het Bureau Patiëntvoorlichting van uw ziekenhuis. De patiëntenvoorlichting van het AMC Amsterdam is te bereiken op telefoonnummer: 020-5663355. Uw behandelend arts kan informatie geven over de patiënten service in uw ziekenhuis. Als (mogelijke) deelnemer aan dit onderzoek kunt u voor inlichtingen en advies ook terecht bij de onafhankelijke arts van de studie Dr. dr. T. Schepers chirurg AMC Amsterdam, te bereiken via telefoonnummer 020-5661895. Deze arts is niet bij de uitvoering van de studie betrokken, maar wel goed op de hoogte. Nadere uitleg kan uiteraard verstrekt worden door de behandelend arts, door ondergetekenden of door de onafhankelijk arts verbonden aan deze studie.

Tot slot
Mocht u na het lezen van deze brief of tijdens de onderzoeksperiode nog nadere informatie willen ontvangen of komen er nog vragen bij u op, dan kunt u altijd contact opnemen met een van de onderstaande contactpersonen of de website www.epochtrial.com bezoeken. Voor advies over deelname aan studie kunt u ook terecht bij een onafhankelijk arts die veel weet over de studie. Zijn gegevens vindt u hieronder.

Contactgegevens onderzoekers:

Studiecoördinator
Niels Wolfhagen
Academisch Medisch Centrum
Afdeling chirurgie
Postbus 22660 1100 DD Amsterdam
Telefoonnummer: 020-5666626
Email: epoch@amc.uva.nl

Hoofdonderzoeker
Prof. dr. M.A. Boermeester, Chirurg
Academisch Medisch Centrum
Afdeling chirurgie
Postbus 22660 1100 DD Amsterdam
Telefoonnummer: 020-5662666
Email: m.a.boermeester@amc.uva.nl

Effect of an Enhanced PeriOperative Care and Health Program

Academisch Medisch Centrum
Universiteit van Amsterdam

<u>Onafhankelijk arts</u>
dr. T. Schepers
Academisch Medisch Centrum, Afdeling chirurgie
Postbus 22660 1100 DD Amsterdam
020-5661895
Email: t.schepers@amc.uva.nl

<u>Functionaris voor de Gegevensbescherming van de instelling:</u>
Mw mr. J.B.M. Inge
Meibergdreef 9, kamer E2-212
1105AZ Amsterdam
Telefoonnummer: 020-5662015
E-mail: fg@amc.nl
Website:www.amc.nl

Wij hopen op uw medewerking.
Met vriendelijke groet namens de studiegroep,

Prof. M.A. Boermeester
Chirurg
Academisch Medisch Centrum

de Jonge SW, *et al. BMJ Open* 2020; 10:e038196. doi: 10.1136/bmjopen-2020-038196

Toestemmingsformulier (Informed Consent)
Studie naar het effect van een geoptimaliseerd zorgprogramma rondom operaties.

Titel studie: EPOCH-trial: Effect of an Enhanced PeriOperative Care and Health protection program

Mijn specialist heeft mij gevraagd deel te nemen aan een studie naar een geoptimaliseerd zorgprogramma . Ik heb de bijgevoegde patiënt informatiebrief gelezen, het betreffende onderzoek besproken met de arts, ik heb de gelegenheid gehad om vragen te stellen en begrepen wat het onderzoek inhoudt.

Ik begrijp dat deelname aan dit onderzoek vrijwillig is en dat ik mij op elk moment, zonder opgave van reden, terug kan trekken uit dit onderzoek. Als ik dit doe, zal dit geen enkele invloed hebben op de verdere behandeling die ik van mijn arts(-en) zal ontvangen. Mijn huisarts wordt op de hoogte gebracht van mijn deelname aan dit onderzoek.

Ik geef toestemming tot inzage in mijn relevante medische gegevens door medewerkers van autoriteiten die belast zijn met de controle van medisch onderzoek. Ik begrijp dat de informatie die ik verstrek, zal worden verwerkt en geanalyseerd op de wijze die nodig is voor dit klinisch onderzoek en in overeenstemming met de Wet Bescherming Persoonsgegevens. Mijn gegevens worden geanonimiseerd en gekoppeld aan een studie nummer.

Ik verklaar deel te willen nemen aan dit onderzoek. Het ondertekenen van dit formulier heeft geen gevolgen voor mijn wettelijke rechten. Ik weet dat ik ook mag beslissen niet mee te doen. Mijn gegevens worden tot 15 jaar na afloop van het onderzoek bewaard.

- Ik geef **wel/geen toestemming*** om mij na dit onderzoek te benaderen voor vervolgonderzoek.
- **Ik geef wel/geen toestemming*** om mijn persoonsgegevens langer te bewaren en te gebruiken voor toekomstig onderzoek op het gebied van wondinfecties**.**
- Ik wil **wel/niet*** ingelicht worden over de eindresultaten van dit onderzoek.

Naam patiënt: ………………
Handtekening: ………………
Datum: … - … - ….

Ik heb de inhoud en het doel van het onderzoek uitgelegd aan bovenstaande patiënt. Hij/zij begrijpt de informatie en is in de gelegenheid gesteld vragen te stellen.

Naam arts: ………………
Naam ziekenhuis: ………………
Handtekening: ………………
Datum: … - … - …

Versie 8, 23-08-2019

de Jonge SW, *et al. BMJ Open* 2020; 10:e038196. doi: 10.1136/bmjopen-2020-038196

de Jonge SW, *et al. BMJ Open* 2020; 10:e038196. doi: 10.1136/bmjopen-2020-038196

Toestemmingsformulier (Informed Consent)

Studie naar het effect van een geoptimaliseerd zorgprogramma rondom operaties.

Titel studie: EPOCH-trial: Effect of an Enhanced PeriOperative Care and Health protection program

Mijn specialist heeft mij gevraagd deel te nemen aan een studie naar een geoptimaliseerd zorgprogramma . Ik heb de bijgevoegde patiënt informatiebrief gelezen, het betreffende onderzoek besproken met de arts, ik heb de gelegenheid gehad om vragen te stellen en begrepen wat het onderzoek inhoudt.

Ik begrijp dat deelname aan dit onderzoek vrijwillig is en dat ik mij op elk moment, zonder opgave van reden, terug kan trekken uit dit onderzoek. Als ik dit doe, zal dit geen enkele invloed hebben op de verdere behandeling die ik van mijn arts(-en) zal ontvangen. Mijn huisarts wordt op de hoogte gebracht van mijn deelname aan dit onderzoek.

Ik geef toestemming tot inzage in mijn relevante medische gegevens door medewerkers van autoriteiten die belast zijn met de controle van medisch onderzoek. Ik begrijp dat de informatie die ik verstrek, zal worden verwerkt en geanalyseerd op de wijze die nodig is voor dit klinisch onderzoek en in overeenstemming met de Wet Bescherming Persoonsgegevens. Mijn gegevens worden geanonimiseerd en gekoppeld aan een studie nummer.

Ik verklaar deel te willen nemen aan dit onderzoek. Het ondertekenen van dit formulier heeft geen gevolgen voor mijn wettelijke rechten. Ik weet dat ik ook mag beslissen niet mee te doen. Mijn gegevens worden tot 15 jaar na afloop van het onderzoek bewaard.

- Ik geef **wel/geen toestemming*** om mij na dit onderzoek te benaderen voor vervolgonderzoek.
- **Ik geef wel/geen toestemming*** om mijn persoonsgegevens langer te bewaren en te gebruiken voor toekomstig onderzoek op het gebied van wondinfecties**.**
- Ik wil **wel/niet*** ingelicht worden over de eindresultaten van dit onderzoek.

Naam patiënt: ………………
Handtekening: ………………
Datum: … - … - ….

Ik heb de inhoud en het doel van het onderzoek uitgelegd aan bovenstaande patiënt. Hij/zij begrijpt de informatie en is in de gelegenheid gesteld vragen te stellen.

Naam arts: ………………
Naam ziekenhuis: ………………
Handtekening: ………………
Datum: … - … - ….

Versie 8, 23-08-2019
