# Supplementary material for: Enhanced PeriOperative Care and Health protection programme for the prevention of surgical site infections after elective abdominal surgery (EPOCH): study protocol of a randomised controlled, multicentre, superiority trial
Source: BMJ Open. 2020 May 25;10(5):e038196. doi: 10.1136/bmjopen-2020-038196 (PMC7252990; doi:10.1136/bmjopen-2020-038196)
Supplement: Author's manuscript [file bmjopen-2020-038196.draft_revisions.pdf]

BMJ Open

BMJ Open is committed to open peer review. As part of this commitment we make the peer review history of every article we publish publicly available.

When an article is published we post the peer reviewers' comments and the authors' responses online. We also post the versions of the paper that were used during peer review. These are the versions that the peer review comments apply to.

The versions of the paper that follow are the versions that were submitted during the peer review process. They are not the versions of record or the final published versions. They should not be cited or distributed as the published version of this manuscript.

BMJ Open is an open access journal and the full, final, typeset and author-corrected version of record of the manuscript is available on our site with no access controls, subscription charges or pay-per-view fees (http://bmjopen.bmj.com).

If you have any questions on BMJ Open's open peer review process please email info.bmjopen@bmj.com

BMJ Open

Enhanced PeriOperative Care and Health protection program for the prevention of surgical site infections after elective abdominal surgery (EPOCH); study protocol of a randomised controlled multicentre superiority trial

Journal:	*BMJ Open*
Manuscript ID	bmjopen-2020-038196
Article Type:	Protocol
Date Submitted by the Author:	02-Mar-2020

Complete List of Authors:	de Jonge, Stijn; Amsterdam UMC - Locatie AMC, Department of Surgery<br>Wolfhagen, Niels; Amsterdam UMC - Locatie AMC, Department of Surgery<br>Boldingh, Quirine; Amsterdam UMC - Locatie AMC, Department of Surgery<br>Bom, Wouter; Amsterdam UMC - Locatie AMC, Department of Surgery<br>Posthuma, Linda; Amsterdam UMC - Locatie AMC, Department of Anesthesiology<br>Scheijmans, Jochem; Amsterdam UMC - Locatie AMC, Department of Surgery<br>van der Leeuw, Bart; Albert Schweitzer Hospital, Department of Anesthesiology<br>van der Hoeven, Joost; Albert Schweitzer Hospital, Department of Surgery<br>Hering, Jens Peter; Dijklander Ziekenhuis, Anesthesiology<br>Sonneveld, Dirk; Dijklander Ziekenhuis, Department of Surgery<br>van Geffen, Otto; Tergooiziekenhuizen, Department of Anesthesiology<br>Hendriks, Eduard; Tergooiziekenhuizen, Department of Surgery<br>Kluyver, Ewoud; Rode Kruis Ziekenhuis, Department of Anesthesiology<br>Demirkiran, Ahmet; Rode Kruis Ziekenhuis, Department of Surgery<br>van Lonkhuijzen, Luc; Amsterdam UMC - Locatie AMC, Department of gynaecologic oncology<br>Slotema, Thomas; Jeroen Bosch Hospital, Department of Anesthesiology<br>Draaisma, Werner; Jeroen Bosch Ziekenhuis, Department of Surgery<br>Koopman, Seppe; Maasstad Ziekenhuis, Department of Anesthesiology<br>van Rossem, Charles; Maasstad Ziekenhuis, Department of Surgery<br>Over, Linda; Gelre Ziekenhuizen, Department of Anesthesiology<br>van Duijvendijk, Peter; Gelre Ziekenhuizen, Department of Surgery<br>Dijkgraaf, Marcel; Amsterdam UMC - Locatie AMC, Clinical Epidemiology, Biostatistics and Bioinformatics<br>Hollmann, Markus; Amsterdam UMC - Locatie AMC, Department of Anesthesiology<br>Boermeester, Marja; Amsterdam UMC - Locatie AMC, Department of Surgery

Keywords:	Adult surgery < SURGERY, Adult anaesthesia < ANAESTHETICS, PREVENTIVE MEDICINE, WOUND MANAGEMENT

I, the Submitting Author has the right to grant and does grant on behalf of all authors of the Work (as defined in the below author licence), an exclusive licence and/or a non-exclusive licence for contributions from authors who are: i) UK Crown employees; ii) where BMJ has agreed a CC-BY licence shall apply, and/or iii) in accordance with the terms applicable for US Federal Government officers or employees acting as part of their official duties; on a worldwide, perpetual, irrevocable, royalty-free basis to BMJ Publishing Group Ltd ("BMJ") its licensees and where the relevant Journal is co-owned by BMJ to the co-owners of the Journal, to publish the Work in this journal and any other BMJ products and to exploit all rights, as set out in our licence.

The Submitting Author accepts and understands that any supply made under these terms is made by BMJ to the Submitting Author unless you are acting as an employee on behalf of your employer or a postgraduate student of an affiliated institution which is paying any applicable article publishing charge ("APC") for Open Access articles. Where the Submitting Author wishes to make the Work available on an Open Access basis (and intends to pay the relevant APC), the terms of reuse of such Open Access shall be governed by a Creative Commons licence – details of these licences and which Creative Commons licence will apply to this Work are set out in our licence referred to above.

Other than as permitted in any relevant BMJ Author's Self Archiving Policies, I confirm this Work has not been accepted for publication elsewhere, is not being considered for publication elsewhere and does not duplicate material already published. I confirm all authors consent to publication of this Work and authorise the granting of this licence.

For peer review only

TITLE PAGE

Enhanced PeriOperative Care and Health protection program for the prevention of surgical site infections after elective abdominal surgery (EPOCH); study protocol of a randomised controlled multicentre superiority trial

S.W. de Jonge[1], N. Wolfhagen[1], Q.J.J. Boldingh[1], W.J. Bom[1], L.M. Posthuma[2], J.C.G. Scheijmans[1], B.M.F. van der Leeuw[3], J.A.B. van der Hoeven[4], J.P. Hering[5], D.J.A. Sonneveld[6], O.E. van Geffen[7], E.R. Hendriks[8], E.B. Kluyver[9], A.Demirkiran[10], L.R.C.W. van Lonkhuijzen[11], T. Slotema[12], W.A. Draaisma[13], J.S.H.A. Koopman[14], C.C. van Rossem[15], L.M. Over[16], P. van Duijvendijk[17], M.G.W. Dijkgraaf[18], M.W. Hollmann[2], M.A. Boermeester[1]

[1] Department of surgery, Amsterdam UMC, location AMC, Amsterdam Gastroenterology & Metabolism, University of Amsterdam, Amsterdam, The Netherlands

[2]Department of anesthesiology, Amsterdam UMC, location AMC, University of Amsterdam, Amsterdam, The Netherlands

[3]Department of anesthesiology, Albert Schweitzer Hospital, Dordrecht, The Netherlands

[4]Department of surgery, Albert Schweitzer Hospital, Dordrecht, The Netherlands

[5]Department of anesthesiology, Dijklander Ziekenhuis, Hoorn, The Netherlands

[6]Department of surgery, Dijklander Ziekenhuis, Hoorn, The Netherlands

[7]Department of anesthesiology, Tergooi Ziekenhuis, Hilversum, The Netherlands

[8]Department of surgery, Tergooi Ziekenhuis, Hilversum, The Netherlands

[9]Department of anesthesiology, Rode Kruis Hospital, The Netherlands

[10]Department of surgery, Rode Kruis Hospital, The Netherlands

[11]Department of gynaecologic oncology, Amsterdam UMC, University of Amsterdam, Centre for Gynaecologic Oncology Amsterdam, Amsterdam, The Netherlands

[12]Department of anesthesiology, Jeroen Bosch Hospital, 's-Hertogenbosch, the Netherland

[13]Department of surgery, Jeroen Bosch Hospital, 's-Hertogenbosch, the Netherland

[14]Department of anesthesiology, Maasstad Ziekenhuis, Rotterdam, the Netherlands

[15]Department of surgery, Maasstad Ziekenhuis, Rotterdam, the Netherlands

[16]Department of anesthesiology, Gelre Ziekenhuizen, Apeldoorn

[17]Department of surgery, Gelre Ziekenhuizen, Apeldoorn, the Netherlands

[18]Department of clinical epidemiology, biostatistics and bioinformatics, Amsterdam UMC, location AMC, University of Amsterdam, Amsterdam, The Netherlands

Word count: 4028 (excluding title page, abstract, article summary, figures and tables)

For peer review only - http://bmjopen.bmj.com/site/about/guidelines.xhtml

Corresponding author during review process: n.wolfhagen@amsterdamumc.nl
Corresponding author: m.a.boermeerster@amsterdamumc.nl

Appendices

1. Protocol regarding additional measurements
2. Informed consent

For peer review only

ABSTRACT

Introduction: Surgical site infections (SSI) are a common postoperative complication. During the development of the new WHO guidelines on SSI prevention also in the Netherlands was concluded that perioperative care in the Netherlands could be optimised beyond the current standard practice. We selected a limited set of readily available, cheap and evidence-based interventions from these new guidelines that are not part of standard practice in the Netherland and formulated an Enhanced PeriOperative Care and Health bundle (EPOCH). Here, we describe the protocol for an open label randomised controlled parallel group superiority trial to test the effect of the EPOCH bundle added to (national) standard care in comparison to standard care alone on the incidence of SSI.

Methods and analysis: EPOCH consists of intraoperative high FiO_2 (0.80), Goal Directed Fluid Therapy, active pre- intra- and postoperative warming, perioperative glucose control and treatment of severe hyperglycemia (>10mmol^{l-1}), and standardised surgical site handling. Patients scheduled for elective abdominal surgery with an incision larger than 5 centimetres are eligible for inclusion. Participants are randomised daily, 1:1 according to variable block sizes, and stratified per participating centre to either EPOCH added to standard care or standard care only. The primary endpoint will be SSI incidence according to the CDC definition within 30 days as part of routine clinical follow-up. Four additional questionnaires will be sent out over the course of 90 days to capture disability and costs. Other secondary endpoints include anastomotic leakage, incidence of incisional hernia, serious adverse events, hospital readmissions, length of stay and cost effectiveness. Analysis of the primary endpoint will be on an intention-to-treat basis.

Ethics and dissemination: Ethics approval granted by the Amsterdam UMC Medical Ethics Committee (reference 2015_121). Results will be disseminated through peer-reviewed journals and summaries shared with stakeholders.

Registration number: Registered the Dutch Trial Register: NL5572

For peer review only

ARTICLE SUMMARY

Strength and limitations of this study

- The intervention under investigation is a bundle of readily available, cheap and evidence-based interventions to reduce surgical site infection rates.

- A randomised controlled trial is used to test the effectiveness of the intervention bundle.

- Because the study investigates a bundle of interventions, inference on the attributive effect of each separate intervention of the bundle is challenging.

- Subjects and researchers cannot be blinded, but outcome assessors are unaware of treatment allocation.

For peer review only

For peer review only - http://bmjopen.bmj.com/site/about/guidelines.xhtml

INTRODUCTION

Background and rationale

Surgical site infections (SSI) cause excess morbidity, mortality, prolonged hospital stay and consequently increase costs.[1 2] In high income countries, 9.4% of all patients undergoing gastrointestinal surgery suffer from SSI.[3] All SSI combined add up to the most frequently reported healthcare associated infection on admission.[4]

Approximately 55% of SSI are deemed preventable with evidence-based strategies.[5] However, individual measures alone have not resulted in a clinically relevant SSI reduction.[6-8] In contrast, efforts that have used systematic approaches, or bundles, have been successful to varying degrees.[9-11] The Institute for Healthcare Improvement developed the care-bundle concept as small set of evidence-based interventions to improve predefined clinical outcomes. When interventions are implemented as a bundle, better outcomes are reported compared to implementation of these same interventions individually.[12] Care bundles have been successfully used to reduce central venous catheter-line infection rates, ventilator-associated pneumonia rates and to improve the outcomes of patients presenting with sepsis.[12-15]

A national care bundle for SSI prevention (Postoperative Wound Infection or POWI bundle) was implemented in 2009 in the Netherlands.[16] The bundle comprises of hygiene discipline (measured by operating theatre door movements), timing of antibiotic prophylaxis (between 15 and 60 minutes prior to incision), normothermia (aimed at a core temperature of 36.5°C postoperatively), and the avoidance of preoperative hair removal.[17] However, a clear national reduction in SSI has not been demonstrated since start.[18] Of these interventions, only normothermia is evidence based.[19-22] For the other three interventions, there is either no evidence at all, or the available evidence indicates there is no strong association with SSI.[23-25]

The development of two new guidelines on SSI prevention by the World Health Organisation (WHO) and the Centers for Disease Control and prevention highlighted that perioperative care can be optimised beyond this limited, four-interventions, Dutch POWI bundle.[26-29] Nonetheless these interventions have not been widely implemented yet. Considering current standards of practice, costs and the potential for implementation, we selected from these guidelines a limited set of readily available, cheap and evidence-based interventions that are not part of current national standard perioperative care and formulated an enhanced perioperative care and health program (EPOCH). EPOCH comprises of intraoperative high FiO_2, intraoperative Goal Directed Fluid Therapy (GDFT), active pre- intra- and postoperative warming, glucose control and treatment of hyperglycemia (>10mmol^{l-1}), in diabetic as well as non-diabetic patients, and wound irrigation before closure using an aqueous antiseptic. Intraoperative high FiO_2, GDFT, active perioperative warming are expected to increase tissue perfusion and hence stimulate wound healing.

From a pragmatic perspective, as control to the EPOCH bundle elements, we chose standard care by discretion of the surgeon and anesthesiologist as the comparator to the same standard care, supplemented with EPOCH. EPOCH interventions and contrasting typical standard care are listed in table 1.

Objectives

Our primary objective is to determine if the EPOCH program added to standard care is superior to standard care with respect to the incidence of SSI as defined by the Centers for Disease Control and prevention (CDC)

For peer review only - http://bmjopen.bmj.com/site/about/guidelines.xhtml

within 30 days post-surgery in patients undergoing elective abdominal surgery with abdominal incisions larger than five centimetres. We hypothesise that the EPOCH program is superior to standard care alone, for the prevention of SSI.[30] Secondary objectives are described in table 2.

Trial Design

The EPOCH trial is designed as an open label pragmatic 1:1 randomised controlled parallel-group multicentre superiority trial.

For peer review only

For peer review only - http://bmjopen.bmj.com/site/about/guidelines.xhtml

METHODS AND ANALYSIS

Study setting

The trial will be conducted in a multicentre setting involving academic, top-clinical and general hospitals throughout the Netherlands. A list of all participating centres and anticipated inclusions will be kept up to date with the Dutch central commission on human research (CCMO) on www.toetsingonline.nl (dossier number: NL52796.018.15). All study sites are expected to be able to perform the EPOCH interventions. When needed, the anesthesiology or surgery expert on the Trial Steering Committee will instruct local clinicians before study initiation.

Eligibility criteria

Inclusion criteria:

In order to be eligible to participate in this study, a subject must meet the following criteria:

- Adult patients
- Scheduled for elective abdominal surgery involving an abdominal incision larger than five centimetres (this includes open surgery as well as laparoscopic surgery with incisions larger than 5 centimetres i.e. for the excision specimen)

Exclusion criteria

Subject meeting any of the following criteria will be excluded from participation in this study:

- The need for emergency surgery
- Scheduled operation concerning a reoperation for complications from recent surgery (within 3 months after the initial procedure)
- Scheduled operation as part of a two staged surgical procedure and with second procedure possibly scheduled before follow-up of primary endpoint.
- The inability to read or understand informed consent material or study questionnaires
- Participation in another study with interference of study interventions
- Pregnancy

For peer review only

Interventions

The EPOCH intervention bundle and typical standard care are listed in table 1. Beyond this, standard SSI prevention measures typically include preoperative antibiotic prophylaxis when indicated, sterile technique, surgical site preparation and adherence to current national four-interventions SSI prevention (POWI) bundle consisting of hygiene discipline (measured by operating theatre door movements), timing of antibiotic prophylaxis (between 15 and 60 minutes prior to incision), normothermia (aimed at a core temperature of 36.5°C postoperatively), and the avoidance of preoperative hair removal. To avoid withholding good care, surgeons and anesthesiologists can provide what they consider standard care at their discretion. However, it will be agreed that, whatever standard care is at the study initiation, this will not be structurally expanded to EPOCH interventions during the study period. We will aim to recruit study sites for collaboration that have not

included the EPOCH interventions in their standard of care. In 2015, at the time of the study design and initiation, none of the interventions that comprise EPOCH were regularly applied in Dutch hospitals.

Given that this is a pragmatic trial, patient care and comfort are prioritised at all times. Patient requests to cease any of the interventions are immediately honoured. Similarly, when a patient in the control group requests measures that would not have been withheld from them outside of the trial (i.e. warming in case of discomfort from cold) these are also honoured. In case of iodine allergy, chlorhexidine gluconate will be used for surgical site handling and vice versa. In the case of active atrial fibrillation, goal directed therapy based on dynamic preload parameters is impossible. Therefore, hypotensive periods after induction are treated with a vasopressive agent (norepinephrine) until a maximum of 0.08 gamma and thereafter with fluids. Beyond this, there are no criteria for discontinuation or modification of the allocated intervention. The interventions included in EPOCH are individually recommended by the WHO and the CDC and are considered safe.[26-28] At all times, the care team is permitted to amend any of the interventions in both groups to guarantee safe anesthesia and surgery.

Participation in trials that intervene with any of the interventions is not permitted. No other restrictions or recommendations are called for due to the pragmatic nature of the trial. Given the size of the study, any co-intervention that may affect the outcome is expected to randomise evenly to both treatment arms.

Table 1: Overview EPOCH interventions and typical standard care

**EPOCH interventions***	**(Expected) Typical standard care**
Intraoperative administration of high FiO2: Patients will be administered a FiO_2 of 80% after intubation during the entire procedure until extubation.	Intraoperative administration of a FiO2 of 30-40%.
Goal directed fluid therapy: Patients will receive hemodynamic therapy based on a goal directed approach. Optimisation is preferably based on dynamic pre-load parameters (pulse pressure variation or systolic pressure variation) derived from arterial catheter measurements. When no arterial catheter is indicated, a goal directed approach using a non-invasive hemodynamic monitoring system may be used. When both an arterial catheter and a non-invasive hemodynamic monitoring system are not available, hypotensive periods following induction should be compensated for by employing a vasopressive agent (norepinephrine) until a maximum of 0.08 gamma before fluids are administered.	Hemodynamic management based on clinical judgement including amongst other things heart rate, blood pressure and fluid balance.
Active pre-, intra- and postoperative warming: Patients will be put under a hot air blanket (for example a bair hugger) from arrival at the pre-operative holding, continued after arrival in the OR, until two hours after surgery in the recovery room. Core temperature is aimed at > 36.5 ^{0}C. If	Active intraoperative warming - but no pre- and postoperative warming -.

For peer review only - http://bmjopen.bmj.com/site/about/guidelines.xhtml

necessary additional legwarmers may be applied. Pre-warmed blankets, replaced when cooled down, can be used as an alternative when a hot air blankets are not available.	
Perioperative blood glucose control: All patients (diabetic and non-diabetic) will be subjected to blood glucose control throughout the procedure and on the first and second postoperative day. Blood glucose will be measured every hour during surgery (at least twice during procedures lasting more than two hours), once after surgery in the recovery room, and twice randomly (not fasting) on postoperative days 1 and 2. Blood glucose measurements over 10mmol^{l-1} will be treated with insulin. Success of glucose interventions will be checked.	Glucose control at discretion of the anesthesiologist
Surgical site handling: An alcohol based antiseptic agent (chlorhexidine gluconate will be used for skin preparation before incision. Before wound closure (after closure of the fascia), tissue will be irrigated with an aqueous antiseptic agent (0.35% - 10% aqueous povidone iodine solution or 1% aqueous chlorhexidine gluconate) followed by lavage with normal saline.	No specific requirements for pre-incisional skin preparation agent or wound irrigation before closure
* EPOCH interventions are supported by evidence-based WHO and CDC guideline recommendations	

Randomisation and treatment allocation

Patients are randomised per day, stratified by centre with a 1:1 ratio using variable block sizes to either intervention or control group with an internet based automated assignment system (CASTOR EDC).[31] The trial management team, or local study staff, will perform randomisation for each day and trial site after all potential participants for that day have decided on trial participation. This ensures allocation concealment.

Participation to the trial and treatment allocation is registered in a specific research section of the electronic medical record. Patients are randomised per day to avoid contamination between groups. Every day, an email will be sent to inform everyone involved in execution of the interventions, including anesthesiologists, surgeons, operation assistants, recovery nurses, and anesthesia and ward nurses, of the treatment allocation for the day.

Outcomes

The primary outcome measure is the difference in the proportion of patients developing a SSI between the two treatment arms as defined by the CDC definition within follow-up of 30 days, which will be evaluated from the Dutch National Surgical Complication Registry (LHCR) with parallel assessment through medical chart review by the trial management team to account for possible registration effect.[30] All study outcomes, their definitions and data collection methods are listed in table 2. Additionally, in a sub-study, perioperative subcutaneous PtO2 (mmHg), mitochondrial PtO2 (mmHg) and perioperative immune response will also be

For peer review only - http://bmjopen.bmj.com/site/about/guidelines.xhtml

studied in a smaller population of 24 patients. Further details regarding these measurements and respective analysis is described in appendix 1.

Table 2: Outcomes EPOCH trial

**Primary outcome**		
Outcome	Definition	Method of data collection
The incidence of surgical site infection within 30 days of follow-up after surgery.	SSI defined by the Centers for Disease Control and prevention (CDC)[30]	LHCR registry and medical chart review
**Secondary outcomes**		
The incidence of surgical site infection within 90 days of follow-up after surgery.	SSI defined by the Centers for Disease Control and prevention (CDC)[30]	LHCR registry and medical chart review
The incidence of surgical site infection within 30 days follow-up after surgery.	SSI defined through a post discharge self-assessment questionnaire[32 33]	Survey including a Dutch translation of the Bluebelle Wound Healing questionnaire[32 33]
The incidence of surgical site infection within 30 days follow-up after surgery.	SSI defined by visual characteristics of definition of SSI of the Centers for Disease Control and prevention (CDC)[30]	Survey including self-reported wound photos.
The incidence of anastomotic leakage within 30 days follow-up after surgery.	Anastomotic leakage defined by the international study group for rectal cancer[34]	LHCR registry and medical chart review
The incidence of incisional hernia within year follow-up after surgery.	Incisional hernia defined by the European Hernia Society[35 36]	LHCR registry and medical chart review
All-cause mortality within one year follow up after surgery.		Data retrieved from Dutch national registry (Basisregistratie Personen, BRP)
Serious adverse events (SAE) within 30 days follow up after surgery.	SAE defined as any event that results in death, is life threatening (at the time of the event), requires prolonged hospitalisation or re-	LHCR registry and medical chart review

For peer review only - http://bmjopen.bmj.com/site/about/guidelines.xhtml

	hospitalisation, results in persistent or significant disability or incapacity, is a congenital anomaly or birth defect, or any other event that may jeopardise the subject or require an intervention to prevent one of the outcomes listed before.	
Length of hospital stay	Number of days between surgery and discharge, and the number of days of potential readmissions.	Data retrieved from medical chart
Related costs	Direct, indirect, medical and non-medical costs. Costs per quality adjusted life year (QALY) constitute the primary health economic outcome.	Hospital health care utilisation retrieved from health informatics system. Survey on out-of-hospital healthcare utilisation and productivity losses from surveys containing the institute for Medical Technology Assessment's (iMTA) Medical Consumption Questionnaire (iMCQ) adjusted to the study setting and the iMTA Productivity Cost Questionnaire (iPCQ).[36] A survey with out-of-pocket expenses will be added to the survey.
Hospital readmissions	Yes or no	Data extraction from medical chart
Health and disability		Gathered with the WHO disability assessment schedule (WHODAS) 2.0 at 30, 60 and 90 days[37]
Health utility and QALY		Gathered with the EuroQol EQ-5D-3L at 30, 60 and 90 days[38]
Additional secondary outcome, depending on additional funding		
All-cause mortality within five years follow up after surgery.		Data retrieved from Dutch national registry (Basisregistratie Personen, BRP)

Participant timeline

A schematic diagram of the participant timeline is depicted in figure 1.

Sample size

Local SSI surveillance data (Unpublished: Amsterdam UMC, Location AMC; 2008-2012) show a 9.1% SSI risk with standard care. A maximum proportion of approximately 55% of SSI is deemed preventable by application of evidence-based strategies.[5] Accounting for a limited degree of contamination of intervention between

For peer review only - http://bmjopen.bmj.com/site/about/guidelines.xhtml

groups and other factors contributing to SSI, not affected by the bundle, we deem a clinically relevant relative risk reduction of 30% reasonable. This 30% relative reduction represents a 9.1% SSI risk with standard care and a 6.37% SSI risk in the intervention group. A sample size of 3000 evaluable patients, 1500 in each arm, is sufficient to detect this 30% difference in proportion of SSI between the two groups using a two-tailed Z-test with 80% power and a 5% level of significance.

A report by the European Centre of Disease prevention and Control (ECDC) indicates SSI incidence in the Netherlands to be considerably higher than 9.1% we used for our sample size estimation. We chose to use the more conservative value to prevent underestimation of the sample size. In addition, the randomisation per day per centre enables within-centre comparison, which is not yet accounted for in the current sample size estimation. Accounting for this may increase power or lower the number of patients required to attain the same power. A sample size recalculation accounting for within-centre effects will be performed after inclusion of the first 1500 patients and evaluation of the sample size assumption (i.e. SSI incidence in control group) by the Data Safety Monitoring Board (DSMB). As the primary outcome falls within regular follow-up visits, no missing data is expected. Therefore, the sample size is not corrected for missing data.

Recruitment

At the study initiation, 11 hospitals committed to trial participation with a combined expected enrolment of 5419 participants over 33 months representing nearly 50% oversampling. Inclusion criteria are broad and straightforward and, given the low risk and potential benefit of the interventions, willingness to participate is expected to be high. No financial incentives are provided to investigators or participants.

Blinding

Due to the nature of the interventions, trial participants and providers will not be blinded. Outcome assessors, ward doctors responsible for LHCR registration, are not involved in execution of the interventions and will be kept unaware of the treatment allocation.

Data collection methods

All data will be collected and stored in a web-based electronic data capture system (CASTOR EDC).[31] Clinical data with regard to safety and efficacy outcomes will be collected manually by the trial management team during routine trial site visits using a standardised electronic case record form (eCRF). Baseline and clinical data will be collected through a detailed export directly from the electronic medical record to avoid human error. After data validation, data will be imported into the eCRF. Any modification to entered data is password secured and can only be processed with a corresponding explanation for the audit trail.

Survey data (from surgeon, anesthesiologist and patients) will be collected via an eCRF on the EDC. When email is not available, printed CRFs via surface mail will be used. A trial nurse will process surface mail responses in the eCRF. The trial management team will track survey responses, send reminders and conduct follow up phone calls when necessary.

For peer review only - http://bmjopen.bmj.com/site/about/guidelines.xhtml

For peer review only

To monitor intervention adherence and potential contamination between groups, a postoperative survey is sent to surgeons and anesthesiologists on execution of the EPOCH interventions and other interventions that may affect the outcome. In addition, relevant process measures (e.g. administered FiO_2, measured temperature perioperative) are extracted from clinical data. For goal directed fluid therapy and surgical site preparation we rely fully on the self-assessment checklist.

For the primary outcome, SSI is prospectively evaluated according to the CDC criteria.[30] SSI are registered as part of routine postoperative care in accordance with the standardised Dutch National Surgical Complication Registration (LHCR). Physicians involved are expected to be familiar with the CDC criteria. Parallel assessment through medical chart review will be performed by research physicians on the trial management team to account for a potential registration effect. Data collection on secondary outcomes is included in table 2.

Retention and withdrawal

The burden of participation in the study will be minimised. With the exception of two glucose measurements postoperatively and active pre- and postoperative warming, the interventions take place while the patient is under anesthesia. No active adherence from the patient is required and non-adherence from patients is therefore not expected. No additional visits to the hospital are required. Respondents are asked to complete four questionnaires over a period of 90 days. The trial management team will provide support and actively remind respondents via email, surface mail and phone calls. No (financial) incentives will be provided. Whenever a subject does decide to withdraw, all data collected up until withdrawal will be used.

The investigator can decide to withdraw a subject from the study for urgent medical reasons or when intraoperative findings indicate that inclusion criteria are not met anymore (e.g. diagnostic laparoscopy, change in operative plan resulting in trans-anal approach), rendering the patient ineligible for the study. Patients withdrawn from the study are replaced.

Data management

Subjects are coded by a numeric randomisation code. Local investigators are responsible for the subject identification log. Data are stored digitally and will be kept by the project leader for 15 years after the inclusion of the last patient.

Statistical methods

All outcomes will be analysed according to the intention-to-treat principle. Binary outcomes will be analysed using log binomial regression to estimate relative risks. If this regression fails to converge, logistic regression will be performed using odds ratio and corresponding confidence interval for statistical interpretation and recalculated relative risk for point estimate interpretation. Continuous outcome data will be analysed using linear regression. Quality of life data will be analysed as repeated measurement using a linear mixed model. In all analyses, statistical uncertainties are expressed in 95% two-sided confidence intervals. P-values of <0.05 will indicate statistical significance.

For peer review only - http://bmjopen.bmj.com/site/about/guidelines.xhtml

Randomisation daily per trial site enables within-centre comparison of treatments and increases power. A sensitivity analysis of the primary outcome will be conducted that accounts for within centre comparison of treatments. When the hypothesis test of this analysis diverges from the primary analysis, all further analysis will also be conducted using this effect estimate. All outcomes will also be analysed according to the per protocol population after adjustment for confounding due to incomplete adherence to the assigned treatments or use of off-protocol concomitant therapies. Variables will be considered for adjustment based on Tyler van de Weele's principles for confounder selection and include preoperative body mass index (BMI), (insulin dependent) diabetes mellitus (DM), cardiovascular heart diseases other than hypertension, chronic obstructive pulmonary disease (COPD) and any other variables that meet these criteria and pass statistical variable selection.[39] Procedure duration is considered an important proxy for complexity of procedure and independent of the interventions comprising EPOCH. Therefore, it will also be considered for adjustment despite being measured during the exposure. In an additional analysis, this method will also be applied to each individual intervention of the EPOCH bundle, the POWI bundle, and other promising interventions outside the bundle under investigation to assess the attributive effect of each intervention separately.

The intention-to-treat population will comprise of all evaluable randomised patients regardless of adherence of treatment allocation. The per protocol population will comprise of all patients who were treated per protocol according to the postoperative adherence surveys and process measures, and all patients in the control group who were not treated with these interventions according to the postoperative adherence surveys and process measures. The safety analysis will be conducted on all patients exposed to the interventions, regardless of evaluable primary outcome. Missing data will be handled by multiple imputation by chained equation when the required assumptions apply. A more detailed description of statistical methods and analyses is described in the statistical analysis plan (SAP). The EPOCH SAP will be published separate from the EPOCH Protocol timely before analysis of the data.

Monitoring

An independent data safety and monitoring board (DSMB) safeguards the interests of trial participants and assesses the safety of the interventions during the trial. The DSMB consists of a statistician, an anesthesiologist and an infectious disease specialist. The DSMB will regularly monitor safety data, recruitment and loss to follow up and make recommendations about continuation of the trial. After every 50 new serious adverse events (any type) or 10 new deaths, the DSMB will re-evaluate. If required, monitoring can also be on ad hoc basis. There will be no interim analysis of the treatment effect. At its own discretion, the DSMB may advice to stop the study prematurely based on its findings. This advice is not bound to specific guidelines other than that it should serve the interest of the trial participants. If the sponsor decides not to (fully) implement the advice, the sponsor will inform the MEC.

Harms

All Serious Adverse Events (SAE) that occur within 30 days after surgery are recorded and reported to the DSMB irrespective of perceived relation to the intervention. SAE are defined as any event within 30 days of the

For peer review only - http://bmjopen.bmj.com/site/about/guidelines.xhtml

intervention that results in death, is life threatening (at the time of the event), requires prolonged hospitalisation or re-hospitalisation, results in persistent or significant disability or incapacity, is a congenital anomaly or birth defect, or any other event that may jeopardise the subject or require an intervention to prevent one of the outcomes listed before based on appropriate medical judgement. SAE will be followed up until they have abated, or until a stable situation has been reached.

All physicians involved in the EPOCH study are instructed to report serious adverse events to the study coordinator. Mortality has to be reported within 48 hours after the occurrence. In addition to spontaneous reporting, the trial management team will review medical charts within 30-day intervals. The MEC will receive a biannual line listing of all reported SAE.

In accordance with the Dutch human research law (WMO), the sponsor will suspend the study if there is sufficient ground that continuation of the study will jeopardise health or safety of study participants. The sponsor will notify the MEC without undue delay of a temporary halt including the reason for such an action. The study will be suspended pending a further decision by the MEC. When appropriate, the investigator will ensure that all subjects will be informed.

Auditing

The Amsterdam UMC clinical research unit may decide to conduct audits and monitoring visits at random, independently of trial coordinator and sponsor. Due to minimal risks of the interventions there are no scheduled monitoring visits.

Patient and Public involvement

The Dutch patient and healthcare consumer federation (NPCF) endorsed the study and provided input on the study proposal.

Rationale for the study design

An alternative to the present study design of 1:1 parallel group randomisation per day per trial site is cluster randomisation. Cluster randomisation can overcome the risk of contamination by randomising study participants by trial site. However, disadvantages of cluster randomisation include the risk of residual confounding due to imbalanced distribution of trial site characteristics (like baseline level of SSI prevention), and the need for a larger sample size to ensure equivalent statistical power.[40-42] This may occur despite large numbers of participants and clusters.[43] Substantial contamination can be tolerated within an individual randomised trial before a cluster design is better in terms of total sample size. By randomising per trial site per day, every patient treated at that particular site by that particular team will receive the same care during the entire day. At the individual patient level, the allocation remains completely random. This design foregoes the weaknesses of full cluster randomisation, while minimizing the risk of contamination of usual care by the EPOCH enhanced care. Furthermore, contamination is taken in to account in the sample size calculation.

For peer review only - http://bmjopen.bmj.com/site/about/guidelines.xhtml

ETHICS AND DISSEMINATION

Ethics

This study will be conducted in accordance with the Declaration of Helsinki (64th version, October 2013) and the laws governing human research in the Netherlands (Wet Medisch-wetenschappelijk Onderzoek met mensen – WMO and Best Clinical Practice (BCP)) and the guidelines of the Central Committee for Research involving Human Subjects (Centrale Commissie Mensgebonden Onderzoek - CCMO). The Amsterdam UMC MEC has approved this trial (reference 2015_121).

All intended protocol modifications with implications beyond administrative changes will be submitted to the MEC and only effectuated after formal approval for the change. Strictly administrative amendments will be notified to the MEC. Local principle investigators will be notified of all amendments. Participants will be notified if necessary. Current version of the protocol is version 10, 17-12-2019. All amendments are described in table 3.

Table 3. Revision chronology EPOCH study protocol

Revision chronology EPOCH study protocol		
Version no.	Date	Main reasons for change
1.	28-03-2015	NA, first submission to MEC
2.	14-07-2015	Modifications requested by MEC d.d. 09-05-2015
3.	28-07-2019	The use of sutures, instead of staples, for skin closure is no longer required in the intervention group. Evidence emerged before the study start that rebuked earlier perceptions on the risk of SSI after the use of staples.
4.	12-11-2015	Administrative modification requested by MEC d.d. 12-11-2015 to comply with renewed law for human research.
5.	22-02-2016	Addition of patient generated wound photos to data collection.
6.	05-07-2018	Addition of a post discharge SSI surveillance checklist at 30 days postoperatively to outcome data for all trial participants and tissue oxygen tension measurement and immunologic analysis to investigate mechanism of potential effect of the interventions for a limited subset of trial participants. Modification of SAE reporting from individual event reports to a list of all events every six months to alleviate administrative pressure.
7.	24-01-2019	Modification of technique for tissue oxygenation measurement and immunologic analysis before first measurement was conducted.
8.	26-05-2019	Modification of DSMB charter at request of the DSMB and modifications of the wording of inclusion criteria and replacement of non-evaluable

For peer review only - http://bmjopen.bmj.com/site/about/guidelines.xhtml

		patients to better reflect practice, and practical modifications to ease administrative burden and logistics.
9.	23-08-2019	Modification of sample size and inclusion criteria for additional measurements in limited subset of trial participants.
10	17-12-2019	Modification of exclusion criteria

Confidentiality

Informed consent forms will be securely stored at local study sites in locked cabinets with limited access. All data will be linked to an anonymised study number. The local investigators are responsible for the subject identification log. All local databases will be secured by password. All other information regarding the trial will be stored securely in a local trial master file. Data collected during this trial will only be used for the objective of this study described in the informed consent unless additional consent of the participant is retrieved. The trial steering committee and study coordinator will control access of the final data set.

Ancillary and post-trial care

No provisions for post-trial care are made. MEC has waived the obligation to take out a special insurance for participants because there are no risks associated with participation to this study.

Dissemination

No arrangements have been made concerning public disclosure. The study is registered in the Dutch trial register as Trial NL5572 (https://www.trialregister.nl/trial/5572). An overview of the WHO trial registration data set is described in table 4. The trial results will be submitted to a peer-reviewed journal regardless of the outcome and made open access available in accordance with the Netherlands Organization for Health Research and innovation (ZonMW) policy. Co-authorship will be based on the international committee of medical journal editors' guidelines. Contributors that do not fulfil these criteria will be listed as collaborator. The order of authors will be based on scientific input.

The study protocol and statistical analysis plan will be published after half of the planned sample size, 1500 evaluable participants, have been enrolled and before any of the outcome data will be available. Participant level data and statistical code will be made available on request after all trials results have been published and a data sharing agreement has been establishment.

For peer review only - http://bmjopen.bmj.com/site/about/guidelines.xhtml

Table 4: WHO trial registration data set

WHO trial registration data set	
Primary registry and trial identifying number	Trial NL5572 (NTR5694)
Date of registration in primary registry	2016-03-03
Secondary identifying numbers	NA
Source(s) of monetary or material support	ZonMW Dutch Healthcare efficacy program<br>Innovation fund Dutch healthcare insurers<br>Ethicon, Johnson & Johnson
Primary sponsor	Amsterdam University Medical Centre, Location AMC
Secondary sponsor(s)	NA
Contact for public queries	m.a.boermeester@amsterdamumc.nl
Contact for scientific queries	m.a.boermeester@amsterdamumc.nl
Public title	Enhanced care around surgery for the prevention of surgical site infections
Scientific title	Enhanced perioperative care for the prevention of surgical site infections, A Pragmatic, randomised controlled parallel-group multicentre superiority trial
Countries of recruitment	The Netherlands
Health condition(s) or problem(s) studied	Surgical Site infection
Intervention(s)	Intervention:<br>An enhanced perioperative care program added to standard care consisting of:<br>- Supplemental oxygen (FiO_2 0.80)<br>- Pre, intra, and postoperative warming<br>- Goal directed fluid therapy<br>- Perioperative glucose control < 10mmol^{l-1}<br>- Surgical wound irrigation<br><br>Control:<br>Standard care at discretion of treating physician
Key inclusion and exclusion criteria	Inclusion criteria:<br>Adult patients undergoing elective open or laparoscopic abdominal surgery with an incision larger than 5 cm.<br><br>Exclusion criteria:<br>- The need for emergency surgery

For peer review only - http://bmjopen.bmj.com/site/about/guidelines.xhtml

	- Scheduled operation concerning a reoperation for complications from recent surgery (within 3 months after the initial procedure)<br>- Scheduled operation as part of a two staged surgical procedure and second procedure possibly scheduled before follow-up of primary endpoint.<br>- The inability to read or understand informed consent material or study questionnaires<br>- Participation in another study with interference of study interventions or outcomes<br>- Pregnancy
Study type	A pragmatic, randomised controlled parallel-group multicentre superiority trial
Date of first enrolment	2016-03-01
Target sample size	3000
Recruitment status	Recruiting
Primary outcome(s)	Incidence of surgical site infections within 30 days evaluated from the Dutch National Surgical Complication Registry (LHCR) with parallel assessment by the CDC definition through medical chart reviews.
Key secondary outcomes	- SSI incidence evaluated at 30 days and 3 months follow-up by the CDC definition through medical chart review.<br>- Readmissions rate within 30 days follow-up through LHCR registration and medical chart review<br>- WHO disability assessment schedule 2.0 by self-administration through online/paper-form questionnaires at postop day 30, 60 and 90.<br>- (In)direct medical and non-medical costs, quality adjusted life years (QALY)<br>- Incidence of anastomotic leakage at 30 days follow-up through LHCR registration and medical chart review.<br>- Incidence of incisional hernia by medical chart review (diagnosed by either physical examination and/or ultrasonography or CT) one year after surgery.

For peer review only

Contribution

MB and MWH conceived of the study. MB, MWH, MD and SJ designed the study. MD provided statistical and methodological expertise. MB provided expertise from the surgical perspective and MW provided expertise from the anesthesiological perspective. MB, MWH, MD, SJ, QB, LP, WB, NW, and JS contributed to the study protocol. All local investigators (BL, JH, JPH, DS, OG, ER, EK, AD, LL, TS, WD, JK, CR, LO, PD) provided essential critical input on the study protocol. MB guided and supervised SJ with the grant application, obtaining medical ethics approval, and writing of the study protocol. SJ, QB, LP, WB, NW and JS will help with implementation and execution of the trial. SJ and NW will conduct the statistical analysis supervised by MD. SJ, NW and QB will draft the final manuscript under supervision of MB, MD and MW.

Funding

The EPOCH trial is funded by the Netherlands Organization for Health Research and innovation (ZonMw; project 843002606) with 10% compulsory co-financing provided by the Innovation Fund of Dutch healthcare insurers and by Ethicon (Johnson & Johnson).

Roles and responsibilities

Roles and responsibilities are described in table 5.

Table 5: roles and responsibilities in the EPOCH trial

Roles and responsibilities in the EPOCH trial	
**Role**	**Details and responsibilities**
Trial sponsor	Trial sponsor: Amsterdam University Medical Centre, Location AMC, Department of Surgery and Department of Anesthesiology<br>Principle Investigators: Prof. dr. M.A. Boermeester and prof. dr. dr. M.W. Hollmann<br>Address: Meibergdreef 9, 1105 AZ Amsterdam, The Netherlands<br>Email address: m.a.boermeester@amsterdamumc.nl, m.w.hollmann@amsterdamumc.nl<br>The sponsor conceived of the study and chairs the trial steering committee. The sponsor is not responsible for data collection, but is responsible for trial management, analysis, interpretations, writing of the report and decision to submit the report for publication. The sponsor does not provide any funding.
Principal investigator	The principal investigators (M.A. Boermeester and M.W. Hollmann) are responsible for the overall conduct of the trial.
Local investigators	The local investigator team consists of an anesthesiologist and a surgeon at each trial site. The local investigators are responsible for local trial execution.
Trial Steering Committee (TSC)	The Trials Steering Committee (TSC) consists of the principal investigators (M.A. Boermeester, Professor of Surgery and Clinical epidemiologist; M.W. Hollmann,

For peer review only - http://bmjopen.bmj.com/site/about/guidelines.xhtml

	Professor of Anesthesiology; M.G. Dijkgraaf, Professor of Health Technology Assessment) and a research physician (S.W. de Jonge, Clinical epidemiologist). The TSC is responsible for design and overall conduct of the trial. This includes overseeing execution of the protocol, preparation of the protocol and potential amendments to the protocol, the statistical analysis plan, yearly progress reports to Amsterdam UMC medical ethics committee (MEC), safety reports to the Data Safety Monitoring Board (DSMB), and the final report.
Study Coordinator	The study coordinator (Q.J.J. Bolding & N. Wolfhagen) will coordinate the overall execution of the trial. This includes communication with local investigators, trial sites, if necessary included patients, and physicians executing the interventions. The study coordinator also oversees follow-up, data collection, data management and recruitment and start-up of new trials sites.
Trial Management Team	The trial management team (Q.J.J. Boldingh, N. Wolfhagen, S.W. de Jonge, W.J. Bom, L.M. Posthuma, and J.C.G. Scheijmans) assists the study coordinator in the overall execution and if necessary assists local investigators with execution. The trial management team also performs screening of potential participants, recruitment of participants, randomisation and allocation of the intervention.
Data Safety Monitoring Board (DSMB)	The DSMB safeguards the interests of trial participants and assesses the safety of the interventions during the trial. In addition, the DSMB performs the recalculation of the sample size. The DSMB consists of two clinicians with relevant subject matter knowledge (J.T.M van der Meer, infectious disease specialist & M. Klimek, anesthesiologist) and a statistician (P.M. Bossuyt). J.T.M. van der Meer chairs the DSMB. The DSMB reports to the trial sponsor.
Independent physician	The independent physician (T. Schepers, trauma surgeon) represents interests of trial participants and mediates conflicts between trial participants and investigators if necessary.

Competing interests

All authors declare no competing interests.

For peer review only - http://bmjopen.bmj.com/site/about/guidelines.xhtml

References

1. de Lissovoy G, Fraeman K, Hutchins V, et al. Surgical site infection: incidence and impact on hospital utilization and treatment costs. *American journal of infection control* 2009;37(5):387-97. doi: 10.1016/j.ajic.2008.12.010
2. O'Brien WJ, Gupta K, Itani KMF. Association of Postoperative Infection With Risk of Long-term Infection and Mortality. *JAMA Surg* 2019:1-8. doi: 10.1001/jamasurg.2019.4539 [published Online First: 2019/11/07]
3. GlobalSurg C. Surgical site infection after gastrointestinal surgery in high-income, middle-income, and low-income countries: a prospective, international, multicentre cohort study. *Lancet Infect Dis* 2018;18(5):516-25. doi: 10.1016/S1473-3099(18)30101-4 [published Online First: 2018/02/18]
4. Suetens C, Latour K, Karki T, et al. Prevalence of healthcare-associated infections, estimated incidence and composite antimicrobial resistance index in acute care hospitals and long-term care facilities: results from two European point prevalence surveys, 2016 to 2017. *Euro Surveill* 2018;23(46) doi: 10.2807/1560-7917.ES.2018.23.46.1800516 [published Online First: 2018/11/22]
5. Umscheid CA, Mitchell MD, Doshi JA, et al. Estimating the proportion of healthcare-associated infections that are reasonably preventable and the related mortality and costs. *Infect Control Hosp Epidemiol* 2011;32(2):101-14. doi: 10.1086/657912
6. Nguyen N, Yegiyants S, Kaloostian C, et al. The Surgical Care Improvement project (SCIP) initiative to reduce infection in elective colorectal surgery: which performance measures affect outcome? *AmSurg* 2008;74(10):1012-16.
7. Stulberg JJ, Delaney CP, Neuhauser DV, et al. Adherence to surgical care improvement project measures and the association with postoperative infections. *JAMA* 2010;303(24):2479-85. doi: 10.1001/jama.2010.841 [published Online First: 2010/06/24]
8. Awad SS. Adherence to surgical care improvement project measures and post-operative surgical site infections. *Surg Infect (Larchmt)* 2012;13(4):234-7. doi: 10.1089/sur.2012.131 [published Online First: 2012/08/24]
9. Bull A, Wilson J, Worth LJ, et al. A bundle of care to reduce colorectal surgical infections: an Australian experience. *J Hosp Infect* 2011;78(4):297-301. doi: 10.1016/j.jhin.2011.03.029 [published Online First: 2011/06/15]
10. Cima R, Dankbar E, Lovely J, et al. Colorectal surgery surgical site infection reduction program: a national surgical quality improvement program--driven multidisciplinary single-institution experience. *J Am Coll Surg* 2013;216(1):23-33. doi: 10.1016/j.jamcollsurg.2012.09.009 [published Online First: 2012/11/07]
11. Keenan JE, Speicher PJ, Thacker JK, et al. The preventive surgical site infection bundle in colorectal surgery: an effective approach to surgical site infection reduction and health care cost savings. *JAMA Surg* 2014;149(10):1045-52. doi: 10.1001/jamasurg.2014.346 [published Online First: 2014/08/28]
12. Resar R, Griffin FA, Haraden C, et al. Using Care Bundles to Improve Health Care Quality. IHI Innovation Series white paper. Cambridge, Massachusetts: Institute for Healthcare Improvement, 2012.
13. Dellinger RP, Levy MM, Rhodes A, et al. Surviving sepsis campaign: international guidelines for management of severe sepsis and septic shock: 2012. *Crit Care Med* 2013;41(2):580-637. doi: 10.1097/CCM.0b013e31827e83af [published Online First: 2013/01/29]

For peer review only - http://bmjopen.bmj.com/site/about/guidelines.xhtml

14. Pronovost P, Needham D, Berenholtz S, et al. An intervention to decrease catheter-related bloodstream infections in the ICU. *N Engl J Med* 2006;355(26):2725-32. doi: 10.1056/NEJMoa061115 [published Online First: 2006/12/29]
15. Morris AC, Hay AW, Swann DG, et al. Reducing ventilator-associated pneumonia in intensive care: impact of implementing a care bundle. *Crit Care Med* 2011;39(10):2218-24. doi: 10.1097/CCM.0b013e3182227d52 [published Online First: 2011/06/15]
16. veiligheidsprogramma V. Praktijkgids 'Voorkomen van wondinfecties na een operatie', 2009.
17. van den Broek P, Bekkers R, Bonten M, et al. Voorkomen van wondinfecties na een operatie: VMSzorg; 2009 [Available from: http://www.vmszorg.nl/_library/5546/web_2009.0111_praktijkgids_powi.pdf.
18. RIVM. PREZIES Resultaten bijeenkomst POWI, 2013.
19. Melling AC, Ali B, Scott EM, et al. Effects of preoperative warming on the incidence of wound infection after clean surgery: A randomised controlled trial. *Lancet* 2001;358(9285):876-80.
20. Kurz A, Sessler DI, Lenhardt R. Perioperative normothermia to reduce the incidence of surgical-wound infection and shorten hospitalization. Study of Wound Infection and Temperature Group. *N Engl J Med* 1996;334(19):1209-15. doi: 10.1056/nejm199605093341901 [published Online First: 1996/05/09]
21. Wong PF, Kumar S, Bohra A, et al. Randomized clinical trial of perioperative systemic warming in major elective abdominal surgery. *Br J Surg* 2007;94(4):421-6. doi: 10.1002/bjs.5631 [published Online First: 2007/03/24]
22. Mahoney CB, Odom J. Maintaining intraoperative normothermia: a meta-analysis of outcomes with costs. *AANA J* 1999;67(2):155-63. [published Online First: 1999/09/17]
23. Hawn MT, Richman JS, Vick CC, et al. Timing of surgical antibiotic prophylaxis and the risk of surgical site infection. *JAMA Surg* 2013;148(7):649-57. doi: 1669977 [pii];10.1001/jamasurg.2013.134 [doi]
24. Tanner J, Norrie P, Melen K. Preoperative hair removal to reduce surgical site infection. *Cochrane Database Syst Rev* 2011(11):CD004122. doi: 10.1002/14651858.CD004122.pub4
25. de Jonge SW, Boldingh QJJ, Koch AH, et al. Timing of Preoperative Antibiotic Prophylaxis and Surgical Site Infection: TAPAS, An Observational Cohort Study. *Ann Surg* 2019 doi: 10.1097/SLA.0000000000003634 [published Online First: 2019/10/31]
26. Berrios-Torres SI, Umscheid CA, Bratzler DW, et al. Centers for Disease Control and Prevention Guideline for the Prevention of Surgical Site Infection, 2017. *JAMA Surg* 2017;152(8):784-91. doi: 10.1001/jamasurg.2017.0904
27. Allegranzi B, Bischoff P, de Jonge S, et al. New WHO recommendations on preoperative measures for surgical site infection prevention: an evidence-based global perspective. *The Lancet Infectious Diseases* 2016;16(12):e276-e87. doi: 10.1016/s1473-3099(16)30398-x
28. Allegranzi B, Zayed B, Bischoff P, et al. New WHO recommendations on intraoperative and postoperative measures for surgical site infection prevention: an evidence-based global perspective. *The Lancet Infectious Diseases* 2016;16(12):e288-e303. doi: 10.1016/s1473-3099(16)30402-9
29. Organization WH. Global Guidelines for the Prevention of Surgical Site Infection. Global Guidelines for the Prevention of Surgical Site Infection. Geneva2016.

For peer review only - http://bmjopen.bmj.com/site/about/guidelines.xhtml

30. Horan TC, Gaynes RP, Martone WJ, et al. CDC definitions of nosocomial surgical site infections, 1992: a modification of CDC definitions of surgical wound infections. *American journal of infection control* 1992;20:271-74. doi: 10.1016/S0196-6553(05)80201-9
31. Castor EDC. Castor Electronic Data Capture 2019 [27 Aug. 2019]. Available from: https://castoredc.com accessed August 28, 2019.
32. Macefield RC, Reeves BC, Milne TK, et al. Development of a single, practical measure of surgical site infection (SSI) for patient report or observer completion. *J Infect Prev* 2017;18(4):170-79. doi: 10.1177/1757177416689724 [published Online First: 2017/10/11]
33. Bluebelle Study G. Validation of the Bluebelle Wound Healing Questionnaire for assessment of surgical-site infection in closed primary wounds after hospital discharge. *Br J Surg* 2019;106(3):226-35. doi: 10.1002/bjs.11008 [published Online First: 2018/12/18]
34. Rahbari NN, Weitz J, Hohenberger W, et al. Definition and grading of anastomotic leakage following anterior resection of the rectum: a proposal by the International Study Group of Rectal Cancer. *Surgery* 2010;147(3):339-51. doi: 10.1016/j.surg.2009.10.012
35. Muysoms FE, Miserez M, Berrevoet F, et al. Classification of primary and incisional abdominal wall hernias. *Hernia* 2009;13(4):407-14. doi: 10.1007/s10029-009-0518-x [published Online First: 2009/06/06]
36. Korenkov M, Paul A, Sauerland S, et al. Classification and surgical treatment of incisional hernia. Results of an experts' meeting. *Langenbecks Arch Surg* 2001;386(1):65-73. doi: 10.1007/s004230000182 [published Online First: 2001/06/19]
37. Organization WH. WHO Disability Assessment Schedule 2.0 (WHODAS 2.0) [Available from: https://www.who.int/classifications/icf/more_whodas/en/November 6th 2019.
38. EuroQol. EQ-5D-3L [Available from: https://euroqol.org/eq-5d-instruments/eq-5d-3l-about/ accessed November 6th 2019.
39. VanderWeele TJ. Principles of confounder selection. *Eur J Epidemiol* 2019;34(3):211-19. doi: 10.1007/s10654-019-00494-6 [published Online First: 2019/03/07]
40. Campbell MK, Piaggio G, Elbourne DR, et al. Consort 2010 statement: extension to cluster randomised trials. *BMJ (Clinical research ed)* 2012;345:e5661. doi: 10.1136/bmj.e5661 [published Online First: 2012/09/07]
41. Carter B. Cluster size variability and imbalance in cluster randomized controlled trials. *Stat Med* 2010;29(29):2984-93. doi: 10.1002/sim.4050 [published Online First: 2010/10/22]
42. Torgerson DJ. Contamination in trials: is cluster randomisation the answer? *BMJ (Clinical research ed)* 2001;322(7282):355-7. doi: 10.1136/bmj.322.7282.355 [published Online First: 2001/02/13]
43. Alexander FE, Anderson TJ, Brown HK, et al. 14 years of follow-up from the Edinburgh randomised trial of breast-cancer screening. *Lancet (London, England)* 1999;353(9168):1903-8. doi: 10.1016/s0140-6736(98)07413-3 [published Online First: 1999/06/17]

For peer review only - http://bmjopen.bmj.com/site/about/guidelines.xhtml

Figure 1. Schedule of enrolment, interventions, and assessments.

	STUDY PERIOD									
	**Enrolment**	**Allocation**	**Post-allocation**							
**TIMEPOINT**	**Up to -1 day**	**-1 day**	**0**	**1 day**	**2 days**	**10 days**	**30 days**	**60 days**	**90 days**	**1 year**
**ENROLMENT:**										
Eligibility screen	X			X						
Informed consent	X									
Allocation		X								
**INTERVENTIONS:**										
Surgical procedure standard care			X							
Surgical procedure standard care supplemented with EPOCH			X	X	X					
**ASSESSMENTS:**										
Baseline characteristics			X							
Perioperative characteristics			X							
Adherence survey surgeon			X							
Adherence survey anesthesiologist			X							
Serious Adverse Event			As needed throughout protocol							
SSI by medical chart review							X		X	
Incidence SSI by survey							X			
Anastomotic leakage							X		X	
Incisional hernia							X			X
EQ-5D-3L						X	X	X	X	
Wound photo						X	X			
WHODAS 2.0							X	X	X	
IMTA-MCQ									X	
IMTA-PCQ									X	

For peer review only

Appendix 1. Additional measurements

Background

Studies have shown a negative correlation between tissue oxygen tension and SSI.[1-4] Conversely, improving tissue oxygen tension has shown reduce the risk of SSI.[5] Several interventions, thought to improve tissue oxygen tension, including hyperoxygenation, active warming and goal directed fluid therapy, have shown to help reduce the risk of SSI.[5-7] However, the evidence for these interventions is based on clinical outcomes and the precise mechanism of effect is not entirely clear. In vitro experiments have indicated that optimized oxygenation may enhance immunologic response,[8] but only one study on these interventions actually measured oxygen tension as a process measure.[5] The study, found a marked increase in oxygen tension and over 50% reduction in SSI.[5] Various investigators have tried to reproduce these results with varying success.[9] Since the early positive results, much has changed in perioperative care with considerable implications for cardiovascular and respiratory physiology. Open procedures with aggressive hydration regimens have made room for laparoscopic surgery with enhanced recovery programs and restricted fluid regimens. These changes could modify the effect on oxygen tension and SSI. The interventions thought to improve oxygen tension, Hyperoxygenation, active warming, and goal directed fluid therapy, are all components of the EPOCH bundle. This poses an extraordinary opportunity to investigate the effect of these interventions combined on tissue oxygen tension and immunologic response in the current era. Further understanding of these underlying mechanisms may facilitate optimization if the interventions prove effective, or understanding if they do not. Her we aim to study the effect of the EPOCH interventions on tissue oxygen tension and immunologic response.

Population

Patients enrolled in the AMC who did not use immunosuppressants in the previous 90 days (e.g. glucocorticoids, cytostatic agents, antibodies, drugs acting on immunophilins, biologicals) will be approached for participation in additional measurements. This includes subcutaneous tissue oxygen tension measurement, mitochondrial tissue oxygen tension measurement and measurement of immunologic effects. Considering the substantial cost involved a small subset of the larger population will be measured based on a separate sample size calculation. Previous studies have shown the standard deviation in mean subcutaneous tissue oxygen tension is approximately 43 mm Hg.[5] Based on an alpha of 0.05, a beta of 0.2 (power of 0.8) and standard deviation in mean subcutaneous tissue oxygen tension of 43 mm Hg, 12 participants per arm are needed to show the previously reported 50 mmHg difference in subcutaneous tissue oxygen tension. Because the EPOCH study includes both laparoscopic and open surgery, and the two approaches have meaningful differences in hemodynamics and hemodynamic management, twenty-four consecutive eligible patients undergoing laparoscopic surgery, and 24 consecutive eligible patients undergoing open surgery will be enrolled until the final sample size is obtained for each. For the measurement of mitochondrial oxygen tension and immunologic effects, no reliable data is available to conduct a sample size estimation. Therefore, both will be measured as a pilot study investigating the 48 participants undergoing subcutaneous tissue oxygen tension measurement. Analysis of these pilot data will inform the need for further data collection.

Outcomes measures

- The difference in mean perioperative subcutaneous PtO_2 (mmHg) at the upper arm between the two treatment arms as measured by the Licox (Integra LifeSciences Corp, Plainsboro, New Jersey).
- The difference in mean perioperative mitochondrial PtO_2 (mmHg) in the epidermis, measured parasternal, between the two treatment arms as measured by the COMET (Photonics Healthcare B.V.).

For peer review only

- The difference in perioperative immune response between the two treatment arms as measured by neutrophil intracellular ROS generation, phagocytosis assays, plasma cytokine concentrations, plasma malondialdehyde concentration, leukocytic HLA-DRA mRNA expression, and ex vivo whole blood stimulation in blood samples withdrawn at five pre-specified timepoints.

Techniques

For subcutaneous tissue oxygen tension measurement, The Licox (Integra LifeSciences Corp, Plainsboro, New Jersey) system will be used. Licox continuously calculates tissue partial tension of oxygen through a Clark type polarographic oxygen electrode in a micro-catheter for oxygen measurements positioned in the tip of a flexible probe. The probe, with an outer diameter of 1.0mm will be inserted in the subcutaneous tissue with a 19-gauge venflon needle after local anaesthesia, and shortly before induction of general anaesthesia. Probes and instruments required for placement are sterile, single use and will be positioned maintaining sterile technique. The probe will be removed before discharge from the recovery room. For mitochondrial tissue oxygen measurement, the Cellular Oxygen METabolism (COMET) device (Photonics Healthcare B.V. (PH) will be used. The COMET is a non-invasive bedside monitoring system. After application of a dermatologic agent aminolevulinic acid (ALA) patch to the patient skin on the night before surgery, a sensor is applied to the skin of the thorax. The skin sensor creates a diverging excitation pulse allowing a superficial optical measurement of the oxygen in the mitochondria of the cells in the epidermis in a circular area of about 5 mm^2. Fluorescence light from the tissue is collected and transmitted via the detection fibre towards the detector system.[10-12] Blood samples (EDTA anticoagulated, LH anticoagulated, PAXgene) will be withdrawn at 5 pre-specified time points in a limited subset of the population (24 patients); along with routine preoperative blood sampling, after induction of anaesthesia but prior to incision, at the end of surgery, one hour postoperatively and at the first postoperative outpatient visit. Neutrophil ROS generation will be quantified using 2',7' dichlorofluoresciein diacetate (DCFH-DA; Sigma, St Louis, MO, USA), as described by Perazzio et al.[13] Neutrophil phagocytosis will be measured using the pHrodo Red *E. coli* BioParticles Phagocytosis Kit for Flow Cytometry according to the manufacturer's instructions (LifeTechnologies, Bleiswijk, the Netherlands). EDTA-anticoagulated blood will be centrifuged immediately after withdrawal (2000 g, 10 minutes, 4 °C). Plasma will be stored at − 80 °C until analysis. Concentrations of cytokines (including but not limited to TNFα, IL-6, IL-8, and IL-10) will be analysed batch-wise using a Luminex assay (detection range 3.2-10000 pg/mL) according to the manufacturer's instructions (Milliplex, Millipore, Billerica, MA, USA). RNA will be isolated for HLA-DRA mRNA expression using the Paxgene Blood RNA kit (Qiagen, Valencia, CA, USA) and transcribed into cDNA using the iScript cDNA Synthesis kit (Bio-rad, Hercules, CA, USA). qPCR analysis was performed using TaqMan gene expression assays (Life Technologies, Paisley, UK) for the reference gene peptidylpropylisomerase B (PPIB) (#Hs00168719_m1) and HLA-DRA (#Hs00219575_m1) on a CFX96 Real-Time PCR Detection System (Bio-Rad, Hercules, CA, USA). Lipid peroxidation will be determined by measuring levels of malondialdehyde (MDA) in EDTA plasma using the Thiobarbituric Acid Reactive Substances (TBARS) Parameter Assay Kit (R&D Systems, Abingdon, UK) according to the manufacturer's instructions. Leukocyte cytokine production capacity will be assessed by ex vivo stimulation of whole blood with Lipopolysacharide (LPS) ex vivo, as described by Kox et al.[14] Blood gas parameters will be analysed in LH anticoagulated blood using the Rapidlab 1200 Series (Bayer Healthcare LLC, Berlin, Germany) according to manufacturer's instructions

References

1. Hunt TK. Surgical wound infections: an overview. Am J Med 1981;70:712-8.

2. Hopf HW, Hunt TK, West JM, et al. Wound tissue oxygen tension predicts the risk of wound infection in surgical patients. Arch Surg 1997;132:997-1004; discussion 5.
3. Govinda R, Kasuya Y, Bala E, et al. Early postoperative subcutaneous tissue oxygen predicts surgical site infection. Anesth Analg 2010;111:946-52.
4. Ives CL, Harrison DK, Stansby GS. Tissue oxygen saturation, measured by near-infrared spectroscopy, and its relationship to surgical-site infections. Br J Surg 2007;94:87-91.
5. Greif R, Akça O, Horn EP, Kurz A, Sessler DI. Supplemental perioperative oxygen to reduce the incidence of surgical-wound infection. N Engl J Med 2000;342:161-7.
6. Kurz A, Sessler DI, Lenhardt R. Perioperative normothermia to reduce the incidence of surgical-wound infection and shorten hospitalization. Study of Wound Infection and Temperature Group. N Engl J Med 1996;334:1209-15.
7. Dalfino L, Giglio MT, Puntillo F, Marucci M, Brienza N. Haemodynamic goal-directed therapy and postoperative infections: earlier is better. A systematic review and meta-analysis. Crit Care 2011;15:R154.
8. Qadan M, Battista C, Gardner SA, Anderson G, Akca O, Polk HC, Jr. Oxygen and surgical site infection: a study of underlying immunologic mechanisms. Anesthesiology 2010;113:369-77.
9. de Jonge S, Egger M, Latif A, et al. Effectiveness of 80% vs 30-35% fraction of inspired oxygen in patients undergoing surgery: an updated systematic review and meta-analysis. Br J Anaesth 2019;122:325-34.
10. Mik EG, Stap J, Sinaasappel M, et al. Mitochondrial PO2 measured by delayed fluorescence of endogenous protoporphyrin IX. Nat Methods 2006;3:939-45.
11. Mik EG, Johannes T, Zuurbier CJ, et al. In vivo mitochondrial oxygen tension measured by a delayed fluorescence lifetime technique. Biophys J 2008;95:3977-90.
12. Harms FA, de Boon WM, Balestra GM, et al. Oxygen-dependent delayed fluorescence measured in skin after topical application of 5-aminolevulinic acid. J Biophotonics 2011;4:731-9.
13. Perazzio SF, Salomao R, Silva NP, Andrade LE. Increased neutrophil oxidative burst metabolism in systemic lupus erythematosus. Lupus 2012;21:1543-51.
14. Kox M, Vrouwenvelder MQ, Pompe JC, van der Hoeven JG, Pickkers P, Hoedemaekers CW. The effects of brain injury on heart rate variability and the innate immune response in critically ill patients. J Neurotrauma 2012;29:747-55.

For peer review only

Patiënt informatie brief
Studie naar het effect van een geoptimaliseerd zorgprogramma rondom operaties op het voorkomen van wondinfecties.

Geachte mevrouw / mijnheer,
U bent door uw behandelend arts gevraagd om mee te doen aan een klinisch medisch wetenschappelijk onderzoek. In deze brief kunt u de informatie over dit onderzoek nog eens rustig nalezen, zodat u een weloverwogen beslissing kunt nemen. Wanneer u dat wilt, betrek dan ook uw eventuele partner of familie in de afweging. Voor algemene informatie verwijzen wij u naar de brochure "Medisch-wetenschappelijk onderzoek; Algemene informatie voor de proefpersoon". Wanneer u deze nog niet in bezit hebt, vraag dan uw arts ernaar.

Achtergrond
Wondinfecties na operaties behoren tot de meest voorkomende ziekenhuisinfecties. In Nederland ontwikkelt ongeveer 9% van alle patiënten die een buikoperatie ondergaat een wondinfectie. Wondinfecties variëren in ernst van een oppervlakkige ontsteking tot abcessen of bloedvergiftiging en geven altijd een verminderd operatie resultaat. Vaak moeten patiënten met een wondinfectie langer in het ziekenhuis blijven, opnieuw opgenomen worden of soms zelfs opnieuw geopereerd worden. Hierdoor zorgen wondinfecties voor onnodig veel pijn, ongemak, extra ziektelast en een aanzienlijke toename in de kosten. Wondinfecties zijn niet altijd te vermijden maar er zijn sterke aanwijzingen dat we onze huidige zorg rond operaties kunnen verbeteren om het risico naar beneden te brengen.

Doel van dit onderzoek
In Nederland is de standaard zorg om wondinfecties te voorkomen nu beschreven in de POWI (Post Operatieve Wond Infectie) bundel. Dit is een bundel van vier maatregelen die het risico op wondinfecties moeten verkleinen. Hierin staat dat patiënten op tijd antibiotica moeten krijgen, de deur tijdens de operatie niet te vaak open mag, u niet voor de operatie geschoren mag worden en dat u na de operatie niet te ver afgekoeld mag zijn. Er zijn sterke aanwijzingen dat andere, relatief eenvoudige, maatregelen tijdens de operatie het risico op wondinfecties terug kunnen brengen. Toch worden ze lang nog niet altijd toegepast. Nog niet alle artsen zijn overtuigd van het effect. Veel van deze maatregelen hebben effect op elkaar. Met deze studie willen we onderzoeken of de maatregelen als bundel elkaar versterken en een sterke vermindering geven op het aantal wondinfecties.

Deze bundel bestaat uit:

- <u>Actieve verwarming rondom de operatie</u>
    - Voor, tijdens en na de operatie krijgt de patiënt verwarmde dekens om te voorkomen dat hij of zij afkoelt

- <u>Extra zuurstof tijdens de operatie</u>
    - Tijdens de operatie geven we de patiënt lucht waar wat extra zuurstof in zit (80% i.p.v. 30%)

For peer review only - http://bmjopen.bmj.com/site/about/guidelines.xhtml

- Bloedsuiker controle tijdens en kort na de operatie
    - Tijdens de operatie houden we het bloedsuikergehalte extra goed in de gaten en geven we de patiënt bloedsuiker verlagende medicijnen als dat nodig is. Na de operatie controleren we nog twee keer of het bloedsuikergehalte in orde is.

- Regulering van uw bloedsomloop op basis van een doelgericht protocol
    - We volgen een duidelijk doelgericht protocol waarin staat wanneer, en hoeveel vocht we toedienen tijdens de operatie om de bloedsomloop optimaal te houden.

- Gestandaardiseerde behandeling van de wond
    - Het wondgebied wordt volgens een gestandaardiseerde manier behandeld: Poetsen met alcohol en wondspoeling met jodium voor het dichtmaken van de wond.

Al deze maatregelen vinden voor het grootste deel plaats terwijl u onder narcose bent.

Daarnaast willen we onderzoeken welke rol patiënten kunnen spelen in het vaststellen van wondinfecties aan de hand van de resultaten van dit onderzoek.

Hoe wordt het onderzoek uitgevoerd?
Alle patiënten die een buikoperatie ondergaan zullen worden gevraagd mee te doen aan dit onderzoek. Om uit te zoeken of onze EPOCH bundel van maatregelen beter werkt dan de standaard zorg worden patiënten in twee groepen verdeeld. De eerste groep patiënten krijgt de standaard zorg zoals de dokters dat nu tijdens de operatie gewend zijn. De tweede groep krijgt de standaard zorg zoals de eerste groep, maar aangevuld met de EPOCH bundel aan extra maatregelen rondom de operatie. Verder gebeurt alles rondom de operatie zoals u dat met uw arts besproken heeft.
Als u besluit mee te doen met het onderzoek, wordt u ingedeeld in een van de twee groepen. Om de verdeling zo eerlijk mogelijk te houden, word deze bepaald door loting. Per dag wordt geloot in welke operatiekamers de standaard zorg wordt toegepast en in welke de EPOCH bundel daaraan toe wordt gevoegd.

Wat is er anders dan de reguliere behandeling die u krijgt?
Afhankelijk van de groep waarin u wordt geloot krijgt u de standaard zorg rondom operaties zoals we die nu gewend zijn, of diezelfde zorg aangevuld met de bundel aan extra maatregelen uit onze studie. Daarnaast wordt u gevraagd om een digitale foto te maken van de wond – indien u daar de technische mogelijkheden toe heeft – na 10 en na 30 dagen, en wanneer u in verband met zorgen om de wond contact hebt gehad met een arts. Met deze foto’s kunnen we het proces van wondgenezing goed vervolgen. Op 30, 60 en 90 dagen na de behandeling wordt u gevraagd een aantal vragenlijsten in te vullen. Dit kan op uw smart Phone via de website of op papier. Deze vragenlijsten hebben betrekking op uw wondgenezing, klachten en welbevinden. Dit zal afhankelijk van de vragenlijst ongeveer 10 of 15 minuten per keer in beslag nemen. Bij de laatste vragenlijsten zitten twee extra vragenlijsten die nog eens 30 minuten in beslag nemen. In totaal 65 minuten verspreid over drie maanden. Hiervoor kunt u benaderd worden door de arts-onderzoeker, die hierdoor inzage heeft in uw persoonsgegevens.

For peer review only - http://bmjopen.bmj.com/site/about/guidelines.xhtml

Effect of an Enhanced PeriOperative Care and Health Program

Academisch Medisch Centrum
Universiteit van Amsterdam

Wat zijn mogelijke voor- en nadelen van deelname aan dit onderzoek?
Bij deelname aan dit onderzoek wordt u geloot tussen de standaard zorg, of de geoptimaliseerde zorg (EPOCH bundel). U heeft dus een 50/50 kans. Wij denken dat de geoptimaliseerde behandeling het risico op wondinfecties zou kunnen verlagen, maar dat moeten we nog bewijzen. Er is dus bij aanvang van dit onderzoek nog geen bewezen voordeel voor deelname aan dit onderzoek. Behalve 3 vingerprikjes voor de bloed glucose controle zijn van de extra maatregelen geen nadelen bekend. Wat wel een mogelijk nadeel van de studie is, is dat het invullen van de vragenlijsten wat extra tijd van u vergt. Verspreid over drie maanden is dat ongeveer een uur.

Wordt u geïnformeerd als er tussentijds voor u relevante informatie over de studie bekend wordt?
Het onderzoek zal zo nauwkeurig mogelijk en volgens een vooraf opgesteld plan verlopen. Maar de situatie kan veranderen. Als dat zo is, bespreken we dat direct met u. U beslist dan zelf of u met het onderzoek wilt stoppen of doorgaan.

Vrijwilligheid van deelname
Uw medewerking aan dit onderzoek is vrijwillig. Als u toestemming geeft om aan dit onderzoek mee te doen, heeft u te allen tijde de vrijheid om op die beslissing terug te komen. U hoeft hiervoor geen reden op te geven. Ook uw behandelend arts kan uw deelname aan het onderzoek stopzetten als deze vindt dat dit ten aanzien van uw gezondheid beter is. Uw arts bespreekt dit dan met u. Een beslissing om uw medewerking te beëindigen zal geen nadelige gevolgen hebben op de verdere behandeling en geen invloed hebben op de zorg en aandacht waarop u in ons ziekenhuis recht hebt.

Wanneer u afziet van deelname?
Als u geen toestemming geeft voor deelname aan het onderzoek, ondergaat u de standaard zorg rondom operaties zoals we dat nu gewend zijn conform de huidige richtlijnen.

Gebruik en bewaren van uw gegevens
Voor dit onderzoek worden uw persoonsgegevens verzameld, gebruikt en bewaard. Het gaat om gegevens zoals uw naam, adres, geboortedatum en om gegevens over uw gezondheid. Het verzamelen, gebruiken en bewaren van uw gegevens is nodig om de vragen die in dit onderzoek worden gesteld te kunnen beantwoorden en de resultaten te kunnen publiceren. Wij vragen voor het gebruik van uw gegevens uw toestemming.

Vertrouwelijkheid van uw gegevens
Om uw privacy te beschermen krijgen uw gegevens een code. Uw naam en andere gegevens die u direct kunnen identificeren worden daarbij weggelaten. Alleen met de sleutel van de code zijn gegevens tot u te herleiden. De sleutel van de code blijft veilig opgeborgen in de lokale onderzoeksinstelling. De gegevens bevatten alleen de code, maar niet uw naam of andere gegevens waarmee u kunt worden geïdentificeerd. Ook in rapporten en publicaties over het onderzoek zijn de gegevens niet tot u te herleiden.

For peer review only - http://bmjopen.bmj.com/site/about/guidelines.xhtml

Toegang tot uw gegevens voor controle
Sommige personen kunnen op de onderzoekslocatie toegang krijgen tot al uw gegevens. Ook tot de gegevens zonder code. Dit is nodig om te kunnen controleren of het onderzoek goed en betrouwbaar is uitgevoerd. Personen die ter controle inzage krijgen in uw gegevens zijn: de commissie die de veiligheid van het onderzoek in de gaten houdt, een controleur die voor het AMC als opdrachtgever werkt, nationale toezichthoudende autoriteiten, bijvoorbeeld, de Inspectie Gezondheidszorg. Zij houden uw gegevens geheim. Wij vragen u voor deze inzage toestemming te geven.

Bewaartermijn gegevens
Uw gegevens moeten 15 jaar worden bewaard op de onderzoekslocatie en 15 jaar bij de opdrachtgever (het AMC).
Het wordt bewaard om daarop in de loop van dit onderzoek nog nieuwe bepalingen te kunnen doen die te maken hebben met dit onderzoek.

Bewaren en gebruik van gegevens voor ander onderzoek
Uw gegevens kunnen na afloop van dit onderzoek ook nog van belang zijn voor ander wetenschappelijk onderzoek op het gebied van wondinfecties. Daarvoor zullen uw gegevens 15 jaar worden bewaard. U kunt op het toestemmingsformulier aangeven of u hier wel of niet mee instemt. Indien u hier niet mee instemt, kunt u gewoon deelnemen aan het huidige onderzoek.

Intrekken toestemming
U kunt uw toestemming voor gebruik van uw persoonsgegevens altijd weer intrekken. Dit geldt voor dit onderzoek en ook voor het bewaren en het gebruik voor het toekomstige onderzoek. De onderzoeksgegevens die zijn verzameld tot het moment dat u uw toestemming intrekt worden nog wel gebruikt in het onderzoek.

Meer informatie over uw rechten bij verwerking van gegevens
Voor algemene informatie over uw rechten bij verwerking van uw persoonsgegevens kunt u de website van de Autoriteit Persoonsgegevens raadplegen.
Bij vragen over uw rechten kunt u contact opnemen met de verantwoordelijke voor de verwerking van uw persoonsgegevens. Voor dit onderzoek is dat het AMC, zie contactgegevens verderop.
Bij vragen of klachten over de verwerking van uw persoonsgegevens raden we u aan eerst contact op te nemen met de onderzoek locatie. U kunt ook contact opnemen met de Functionaris voor de Gegevensbescherming van het AMC (zie contactgegevens) of de Autoriteit Persoonsgegevens.

Registratie van het onderzoek
Informatie over dit onderzoek is ook opgenomen in een overzicht van medisch-wetenschappelijke onderzoeken namelijk (www.trialregister.nl). Daarin zijn geen gegevens opgenomen die naar u herleidbaar zijn. Na het onderzoek kan de website een samenvatting van de resultaten van dit onderzoek tonen. U vindt dit onderzoek onder nummer NTR5694.

For peer review only - http://bmjopen.bmj.com/site/about/guidelines.xhtml

Bent u verzekerd wanneer u aan het onderzoek meedoet?
Aangezien aan deelname aan deze studie geen risico's verbonden zijn, heeft de Medisch Ethische Toetsingscommissie ontheffing verleend van de verplichting om voor de deelnemers een speciale schadeverzekering af te sluiten

Is er een vergoeding wanneer u besluit aan dit onderzoek mee te doen?
Er worden geen vergoedingen gedaan voor het meedoen aan dit onderzoek of voor uw reiskosten.

Hoe te handelen bij klachten?
Als u klachten heeft over het onderzoek, kunt u dit melden aan de onderzoeker. Wilt u dit liever niet, dan kunt u contact opnemen met het Bureau Patiëntvoorlichting van uw ziekenhuis. De patiëntenvoorlichting van het AMC Amsterdam is te bereiken op telefoonnummer: 020-5663355. Uw behandelend arts kan informatie geven over de patiënten service in uw ziekenhuis. Als (mogelijke) deelnemer aan dit onderzoek kunt u voor inlichtingen en advies ook terecht bij de onafhankelijke arts van de studie Dr. dr. T. Schepers chirurg AMC Amsterdam, te bereiken via telefoonnummer 020-5661895. Deze arts is niet bij de uitvoering van de studie betrokken, maar wel goed op de hoogte. Nadere uitleg kan uiteraard verstrekt worden door de behandelend arts, door ondergetekenden of door de onafhankelijk arts verbonden aan deze studie.

Tot slot
Mocht u na het lezen van deze brief of tijdens de onderzoeksperiode nog nadere informatie willen ontvangen of komen er nog vragen bij u op, dan kunt u altijd contact opnemen met een van de onderstaande contactpersonen of de website www.epochtrial.com bezoeken. Voor advies over deelname aan studie kunt u ook terecht bij een onafhankelijk arts die veel weet over de studie. Zijn gegevens vindt u hieronder.

Contactgegevens onderzoekers:
Studiecoördinator
Niels Wolfhagen
Academisch Medisch Centrum
Afdeling chirurgie
Postbus 22660 1100 DD Amsterdam
Telefoonnummer: 020-5666626
Email: epoch@amc.uva.nl

Hoofdonderzoeker
Prof. dr. M.A. Boermeester, Chirurg
Academisch Medisch Centrum
Afdeling chirurgie
Postbus 22660 1100 DD Amsterdam
Telefoonnummer: 020-5662666
Email: m.a.boermeester@amc.uva.nl

For peer review only - http://bmjopen.bmj.com/site/about/guidelines.xhtml

Effect of an Enhanced PeriOperative Care and Health Program

Academisch Medisch Centrum
Universiteit van Amsterdam

Onafhankelijk arts
dr. T. Schepers
Academisch Medisch Centrum, Afdeling chirurgie
Postbus 22660 1100 DD Amsterdam
020-5661895
Email: t.schepers@amc.uva.nl

Functionaris voor de Gegevensbescherming van de instelling:
Mw mr. J.B.M. Inge
Meibergdreef 9, kamer E2-212
1105AZ Amsterdam
Telefoonnummer: 020-5662015
E-mail: fg@amc.nl
Website:www.amc.nl

Wij hopen op uw medewerking.
Met vriendelijke groet namens de studiegroep,

Prof. M.A. Boermeester
Chirurg
Academisch Medisch Centrum

For peer review only - http://bmjopen.bmj.com/site/about/guidelines.xhtml

Toestemmingsformulier (Informed Consent)
Studie naar het effect van een geoptimaliseerd zorgprogramma rondom operaties.

Titel studie: EPOCH-trial: Effect of an Enhanced PeriOperative Care and Health protection program

Mijn specialist heeft mij gevraagd deel te nemen aan een studie naar een geoptimaliseerd zorgprogramma . Ik heb de bijgevoegde patiënt informatiebrief gelezen, het betreffende onderzoek besproken met de arts, ik heb de gelegenheid gehad om vragen te stellen en begrepen wat het onderzoek inhoudt.

Ik begrijp dat deelname aan dit onderzoek vrijwillig is en dat ik mij op elk moment, zonder opgave van reden, terug kan trekken uit dit onderzoek. Als ik dit doe, zal dit geen enkele invloed hebben op de verdere behandeling die ik van mijn arts(-en) zal ontvangen. Mijn huisarts wordt op de hoogte gebracht van mijn deelname aan dit onderzoek.

Ik geef toestemming tot inzage in mijn relevante medische gegevens door medewerkers van autoriteiten die belast zijn met de controle van medisch onderzoek. Ik begrijp dat de informatie die ik verstrek, zal worden verwerkt en geanalyseerd op de wijze die nodig is voor dit klinisch onderzoek en in overeenstemming met de Wet Bescherming Persoonsgegevens. Mijn gegevens worden geanonimiseerd en gekoppeld aan een studie nummer.

Ik verklaar deel te willen nemen aan dit onderzoek. Het ondertekenen van dit formulier heeft geen gevolgen voor mijn wettelijke rechten. Ik weet dat ik ook mag beslissen niet mee te doen. Mijn gegevens worden tot 15 jaar na afloop van het onderzoek bewaard.

- Ik geef **wel/geen toestemming*** om mij na dit onderzoek te benaderen voor vervolgonderzoek.
- **Ik geef wel/geen toestemming*** om mijn persoonsgegevens langer te bewaren en te gebruiken voor toekomstig onderzoek op het gebied van wondinfecties**.**
- Ik wil **wel/niet*** ingelicht worden over de eindresultaten van dit onderzoek.

Naam patiënt: ………………
Handtekening: ………………
Datum: … - … - ….

Ik heb de inhoud en het doel van het onderzoek uitgelegd aan bovenstaande patiënt. Hij/zij begrijpt de informatie en is in de gelegenheid gesteld vragen te stellen.

Naam arts: ………………
Naam ziekenhuis: ………………
Handtekening: ………………
Datum: … - … - …

For peer review only - http://bmjopen.bmj.com/site/about/guidelines.xhtml

For peer review only

8/9

For peer review only - http://bmjopen.bmj.com/site/about/guidelines.xhtml

EPOCH
Effect of an Enhanced PeriOperative Care and Health Program

amc
Academisch Medisch Centrum
Universiteit van Amsterdam

Toestemmingsformulier (Informed Consent)

Studie naar het effect van een geoptimaliseerd zorgprogramma rondom operaties.

Titel studie: EPOCH-trial: Effect of an Enhanced PeriOperative Care and Health protection program

Mijn specialist heeft mij gevraagd deel te nemen aan een studie naar een geoptimaliseerd zorgprogramma . Ik heb de bijgevoegde patiënt informatiebrief gelezen, het betreffende onderzoek besproken met de arts, ik heb de gelegenheid gehad om vragen te stellen en begrepen wat het onderzoek inhoudt.

Ik begrijp dat deelname aan dit onderzoek vrijwillig is en dat ik mij op elk moment, zonder opgave van reden, terug kan trekken uit dit onderzoek. Als ik dit doe, zal dit geen enkele invloed hebben op de verdere behandeling die ik van mijn arts(-en) zal ontvangen. Mijn huisarts wordt op de hoogte gebracht van mijn deelname aan dit onderzoek.

Ik geef toestemming tot inzage in mijn relevante medische gegevens door medewerkers van autoriteiten die belast zijn met de controle van medisch onderzoek. Ik begrijp dat de informatie die ik verstrek, zal worden verwerkt en geanalyseerd op de wijze die nodig is voor dit klinisch onderzoek en in overeenstemming met de Wet Bescherming Persoonsgegevens. Mijn gegevens worden geanonimiseerd en gekoppeld aan een studie nummer.

Ik verklaar deel te willen nemen aan dit onderzoek. Het ondertekenen van dit formulier heeft geen gevolgen voor mijn wettelijke rechten. Ik weet dat ik ook mag beslissen niet mee te doen. Mijn gegevens worden tot 15 jaar na afloop van het onderzoek bewaard.

- Ik geef **wel/geen toestemming*** om mij na dit onderzoek te benaderen voor vervolgonderzoek.
- **Ik geef wel/geen toestemming*** om mijn persoonsgegevens langer te bewaren en te gebruiken voor toekomstig onderzoek op het gebied van wondinfecties.
- Ik wil **wel/niet*** ingelicht worden over de eindresultaten van dit onderzoek.

Naam patiënt: ………………
Handtekening: ………………
Datum: … - … - ….

Ik heb de inhoud en het doel van het onderzoek uitgelegd aan bovenstaande patiënt. Hij/zij begrijpt de informatie en is in de gelegenheid gesteld vragen te stellen.

Naam arts: ………………
Naam ziekenhuis: ………………
Handtekening: ………………
Datum: … - … - ….

For peer review only - http://bmjopen.bmj.com/site/about/guidelines.xhtml

STANDARD PROTOCOL ITEMS: RECOMMENDATIONS FOR INTERVENTIONAL TRIALS

SPIRIT 2013 Checklist: Recommended items to address in a clinical trial protocol and related documents*

Section/item	Item No	Description	Report page no
**Administrative information**			
Title	1	Descriptive title identifying the study design, population, interventions, and, if applicable, trial acronym	1
Trial registration	2a	Trial identifier and registry name. If not yet registered, name of intended registry	3
	2b	All items from the World Health Organization Trial Registration Data Set	18
Protocol version	3	Date and version identifier	16
Funding	4	Sources and types of financial, material, and other support	20
Roles and responsibilities	5a	Names, affiliations, and roles of protocol contributors	1
	5b	Name and contact information for the trial sponsor	20
	5c	Role of study sponsor and funders, if any, in study design; collection, management, analysis, and interpretation of data; writing of the report; and the decision to submit the report for publication, including whether they will have ultimate authority over any of these activities	20

For peer review only

	5d	Composition, roles, and responsibilities of the coordinating centre, steering committee, endpoint adjudication committee, data management team, and other individuals or groups overseeing the trial, if applicable (see Item 21a for data monitoring committee)	20
**Introduction**			
Background and rationale	6a	Description of research question and justification for undertaking the trial, including summary of relevant studies (published and unpublished) examining benefits and harms for each intervention	5
	6b	Explanation for choice of comparators	5
Objectives	7	Specific objectives or hypotheses	6
Trial design	8	Description of trial design including type of trial (eg, parallel group, crossover, factorial, single group), allocation ratio, and framework (eg, superiority, equivalence, noninferiority, exploratory)	6
**Methods: Participants, interventions, and outcomes**			
Study setting	9	Description of study settings (eg, community clinic, academic hospital) and list of countries where data will be collected. Reference to where list of study sites can be obtained	7
Eligibility criteria	10	Inclusion and exclusion criteria for participants. If applicable, eligibility criteria for study centres and individuals who will perform the interventions (eg, surgeons, psychotherapists)	7
Interventions	11a	Interventions for each group with sufficient detail to allow replication, including how and when they will be administered	7, 8

For peer review only

	11b	Criteria for discontinuing or modifying allocated interventions for a given trial participant (eg, drug dose change in response to harms, participant request, or improving/worsening disease)	8
	11c	Strategies to improve adherence to intervention protocols, and any procedures for monitoring adherence (eg, drug tablet return, laboratory tests)	9
	11d	Relevant concomitant care and interventions that are permitted or prohibited during the trial	8
Outcomes	12	Primary, secondary, and other outcomes, including the specific measurement variable (eg, systolic blood pressure), analysis metric (eg, change from baseline, final value, time to event), method of aggregation (eg, median, proportion), and time point for each outcome. Explanation of the clinical relevance of chosen efficacy and harm outcomes is strongly recommended	10-11
Participant timeline	13	Time schedule of enrolment, interventions (including any run-ins and washouts), assessments, and visits for participants. A schematic diagram is highly recommended (see Figure)	11 + attachment figure 1
Sample size	14	Estimated number of participants needed to achieve study objectives and how it was determined, including clinical and statistical assumptions supporting any sample size calculations	12
Recruitment	15	Strategies for achieving adequate participant enrolment to reach target sample size	12
**Methods: Assignment of interventions (for controlled trials)**			
Allocation:			

Sequence generation	16a	Method of generating the allocation sequence (eg, computer-generated random numbers), and list of any factors for stratification. To reduce predictability of a random sequence, details of any planned restriction (eg, blocking) should be provided in a separate document that is unavailable to those who enrol participants or assign interventions	9
Allocation concealment mechanism	16b	Mechanism of implementing the allocation sequence (eg, central telephone; sequentially numbered, opaque, sealed envelopes), describing any steps to conceal the sequence until interventions are assigned	9
Implementation	16c	Who will generate the allocation sequence, who will enrol participants, and who will assign participants to interventions	9
Blinding (masking)	17a	Who will be blinded after assignment to interventions (eg, trial participants, care providers, outcome assessors, data analysts), and how	12
	17b	If blinded, circumstances under which unblinding is permissible, and procedure for revealing a participant's allocated intervention during the trial	
**Methods: Data collection, management, and analysis**			
Data collection methods	18a	Plans for assessment and collection of outcome, baseline, and other trial data, including any related processes to promote data quality (eg, duplicate measurements, training of assessors) and a description of study instruments (eg, questionnaires, laboratory tests) along with their reliability and validity, if known. Reference to where data collection forms can be found, if not in the protocol	13
	18b	Plans to promote participant retention and complete follow-up, including list of any outcome data to be collected for participants who discontinue or deviate from intervention protocols	13

Data management	19	Plans for data entry, coding, security, and storage, including any related processes to promote data quality (eg, double data entry; range checks for data values). Reference to where details of data management procedures can be found, if not in the protocol	13
Statistical methods	20a	Statistical methods for analysing primary and secondary outcomes. Reference to where other details of the statistical analysis plan can be found, if not in the protocol	13-14
	20b	Methods for any additional analyses (eg, subgroup and adjusted analyses)	14
	20c	Definition of analysis population relating to protocol non-adherence (eg, as randomised analysis), and any statistical methods to handle missing data (eg, multiple imputation)	14
**Methods: Monitoring**			
Data monitoring	21a	Composition of data monitoring committee (DMC); summary of its role and reporting structure; statement of whether it is independent from the sponsor and competing interests; and reference to where further details about its charter can be found, if not in the protocol. Alternatively, an explanation of why a DMC is not needed	14
	21b	Description of any interim analyses and stopping guidelines, including who will have access to these interim results and make the final decision to terminate the trial	15
Harms	22	Plans for collecting, assessing, reporting, and managing solicited and spontaneously reported adverse events and other unintended effects of trial interventions or trial conduct	15

For peer review only - http://bmjopen.bmj.com/site/about/guidelines.xhtml

Auditing	23	Frequency and procedures for auditing trial conduct, if any, and whether the process will be independent from investigators and the sponsor	15
**Ethics and dissemination**			
Research ethics approval	24	Plans for seeking research ethics committee/institutional review board (REC/IRB) approval	16
Protocol amendments	25	Plans for communicating important protocol modifications (eg, changes to eligibility criteria, outcomes, analyses) to relevant parties (eg, investigators, REC/IRBs, trial participants, trial registries, journals, regulators)	16
Consent or assent	26a	Who will obtain informed consent or assent from potential trial participants or authorised surrogates, and how (see Item 32)	9
	26b	Additional consent provisions for collection and use of participant data and biological specimens in ancillary studies, if applicable	Appendix 2
Confidentiality	27	How personal information about potential and enrolled participants will be collected, shared, and maintained in order to protect confidentiality before, during, and after the trial	17
Declaration of interests	28	Financial and other competing interests for principal investigators for the overall trial and each study site	21
Access to data	29	Statement of who will have access to the final trial dataset, and disclosure of contractual agreements that limit such access for investigators	17
Ancillary and post-trial care	30	Provisions, if any, for ancillary and post-trial care, and for compensation to those who suffer harm from trial participation	17

For peer review only

Dissemination polic	31a	Plans for investigators and sponsor to communicate trial results to participants, healthcare professionals, the public, and other relevant groups (eg, via publication, reporting in results databases, or other data sharing arrangements), including any publication restrictions	17
	31b	Authorship eligibility guidelines and any intended use of professional writers	17
	31c	Plans, if any, for granting public access to the full protocol, participant-level dataset, and statistical code	17
**Appendices**			
Informed consent materials	32	Model consent form and other related documentation given to participants and authorised surrogates	Appendix 2
Biological specimens	33	Plans for collection, laboratory evaluation, and storage of biological specimens for genetic or molecular analysis in the current trial and for future use in ancillary studies, if applicable	Appendix 1

*It is strongly recommended that this checklist be read in conjunction with the SPIRIT 2013 Explanation & Elaboration for important clarification on the items. Amendments to the protocol should be tracked and dated. The SPIRIT checklist is copyrighted by the SPIRIT Group under the Creative Commons "Attribution-NonCommercial-NoDerivs 3.0 Unported" license.

For peer review only

BMJ Open

Enhanced PeriOperative Care and Health protection program for the prevention of surgical site infections after elective abdominal surgery (EPOCH); study protocol of a randomised controlled multicentre superiority trial

Journal:	*BMJ Open*
Manuscript ID	bmjopen-2020-038196.R1
Article Type:	Protocol
Date Submitted by the Author:	16-Apr-2020
Complete List of Authors:	de Jonge, Stijn; Amsterdam UMC - Locatie AMC, Department of Surgery<br>Wolfhagen, Niels; Amsterdam UMC - Locatie AMC, Department of Surgery<br>Boldingh, Quirine; Amsterdam UMC - Locatie AMC, Department of Surgery<br>Bom, Wouter; Amsterdam UMC - Locatie AMC, Department of Surgery<br>Posthuma, Linda; Amsterdam UMC - Locatie AMC, Department of Anesthesiology<br>Scheijmans, Jochem; Amsterdam UMC - Locatie AMC, Department of Surgery<br>van der Leeuw, Bart; Albert Schweitzer Hospital, Department of Anesthesiology<br>van der Hoeven, Joost; Albert Schweitzer Hospital, Department of Surgery<br>Hering, Jens Peter; Dijklander Ziekenhuis, Anesthesiology<br>Sonneveld, Dirk; Dijklander Ziekenhuis, Department of Surgery<br>van Geffen, Otto; Tergooiziekenhuizen, Department of Anesthesiology<br>Hendriks, Eduard; Tergooiziekenhuizen, Department of Surgery<br>Kluyver, Ewoud; Rode Kruis Ziekenhuis, Department of Anesthesiology<br>Demirkiran, Ahmet; Rode Kruis Ziekenhuis, Department of Surgery<br>van Lonkhuijzen, Luc; Amsterdam UMC - Locatie AMC, Department of gynaecologic oncology<br>Slotema, Thomas; Jeroen Bosch Hospital, Department of Anesthesiology<br>Draaisma, Werner; Jeroen Bosch Ziekenhuis, Department of Surgery<br>Koopman, Seppe; Maasstad Ziekenhuis, Department of Anesthesiology<br>van Rossem, Charles; Maasstad Ziekenhuis, Department of Surgery<br>Over, Linda; Gelre Ziekenhuizen, Department of Anesthesiology<br>van Duijvendijk, Peter; Gelre Ziekenhuizen, Department of Surgery<br>Dijkgraaf, Marcel; Amsterdam UMC - Locatie AMC, Clinical Epidemiology, Biostatistics and Bioinformatics<br>Hollmann, Markus; Amsterdam UMC - Locatie AMC, Department of Anesthesiology<br>Boermeester, Marja; Amsterdam UMC - Locatie AMC, Department of Surgery
**Primary Subject Heading**:	Surgery

Secondary Subject Heading:	Anaesthesia
Keywords:	Adult surgery < SURGERY, Adult anaesthesia < ANAESTHETICS, PREVENTIVE MEDICINE, WOUND MANAGEMENT

SCHOLARONE™
Manuscripts

I, the Submitting Author has the right to grant and does grant on behalf of all authors of the Work (as defined in the below author licence), an exclusive licence and/or a non-exclusive licence for contributions from authors who are: i) UK Crown employees; ii) where BMJ has agreed a CC-BY licence shall apply, and/or iii) in accordance with the terms applicable for US Federal Government officers or employees acting as part of their official duties; on a worldwide, perpetual, irrevocable, royalty-free basis to BMJ Publishing Group Ltd ("BMJ") its licensees and where the relevant Journal is co-owned by BMJ to the co-owners of the Journal, to publish the Work in this journal and any other BMJ products and to exploit all rights, as set out in our licence.

The Submitting Author accepts and understands that any supply made under these terms is made by BMJ to the Submitting Author unless you are acting as an employee on behalf of your employer or a postgraduate student of an affiliated institution which is paying any applicable article publishing charge ("APC") for Open Access articles. Where the Submitting Author wishes to make the Work available on an Open Access basis (and intends to pay the relevant APC), the terms of reuse of such Open Access shall be governed by a Creative Commons licence – details of these licences and which Creative Commons licence will apply to this Work are set out in our licence referred to above.

Other than as permitted in any relevant BMJ Author's Self Archiving Policies, I confirm this Work has not been accepted for publication elsewhere, is not being considered for publication elsewhere and does not duplicate material already published. I confirm all authors consent to publication of this Work and authorise the granting of this licence.

For peer review only

TITLE PAGE

Enhanced PeriOperative Care and Health protection program for the prevention of surgical site infections after elective abdominal surgery (EPOCH); study protocol of a randomised controlled multicentre superiority trial

S.W. de Jonge[1], N. Wolfhagen[1], Q.J.J. Boldingh[1], W.J. Bom[1], L.M. Posthuma[2], J.C.G. Scheijmans[1], B.M.F. van der Leeuw[3], J.A.B. van der Hoeven[4], J.P. Hering[5], D.J.A. Sonneveld[6], O.E. van Geffen[7], E.R. Hendriks[8], E.B. Kluyver[9], A.Demirkiran[10], L.R.C.W. van Lonkhuijzen[11], T. Slotema[12], W.A. Draaisma[13], J.S.H.A. Koopman[14], C.C. van Rossem[15], L.M. Over[16], P. van Duijvendijk[17], M.G.W. Dijkgraaf[18], M.W. Hollmann[2], M.A. Boermeester[1]

[1] Department of surgery, Amsterdam UMC, location AMC, Amsterdam Gastroenterology & Metabolism, University of Amsterdam, Amsterdam, The Netherlands

[2]Department of anesthesiology, Amsterdam UMC, location AMC, University of Amsterdam, Amsterdam, The Netherlands

[3]Department of anesthesiology, Albert Schweitzer Hospital, Dordrecht, The Netherlands

[4]Department of surgery, Albert Schweitzer Hospital, Dordrecht, The Netherlands

[5]Department of anesthesiology, Dijklander Ziekenhuis, Hoorn, The Netherlands

[6]Department of surgery, Dijklander Ziekenhuis, Hoorn, The Netherlands

[7]Department of anesthesiology, Tergooi Ziekenhuis, Hilversum, The Netherlands

[8]Department of surgery, Tergooi Ziekenhuis, Hilversum, The Netherlands

[9]Department of anesthesiology, Rode Kruis Hospital, The Netherlands

[10]Department of surgery, Rode Kruis Hospital, The Netherlands

[11]Department of gynaecologic oncology, Amsterdam UMC, University of Amsterdam, Centre for Gynaecologic Oncology Amsterdam, Amsterdam, The Netherlands

[12]Department of anesthesiology, Jeroen Bosch Hospital, 's-Hertogenbosch, the Netherland

[13]Department of surgery, Jeroen Bosch Hospital, 's-Hertogenbosch, the Netherland

[14]Department of anesthesiology, Maasstad Ziekenhuis, Rotterdam, the Netherlands

[15]Department of surgery, Maasstad Ziekenhuis, Rotterdam, the Netherlands

[16]Department of anesthesiology, Gelre Ziekenhuizen, Apeldoorn

[17]Department of surgery, Gelre Ziekenhuizen, Apeldoorn, the Netherlands

[18]Department of clinical epidemiology, biostatistics and bioinformatics, Amsterdam UMC, location AMC, University of Amsterdam, Amsterdam, The Netherlands

Word count: 4193 (excluding title page, abstract, article summary, figures and tables)

Corresponding author: M.A. Boermeester ; m.a.boermeester@amsterdamumc.nl

For peer review only

For peer review only - http://bmjopen.bmj.com/site/about/guidelines.xhtml

Appendices

1. Protocol regarding additional measurements
2. Informed consent form

For peer review only

For peer review only - http://bmjopen.bmj.com/site/about/guidelines.xhtml

ABSTRACT

Introduction: Surgical site infections (SSI) are a common postoperative complication. During the development of the new WHO guidelines on SSI prevention, also in the Netherlands was concluded that perioperative care could be optimised beyond the current standard practice. We selected a limited set of readily available, cheap and evidence-based interventions from these new guidelines that are not part of standard practice in the Netherlands and formulated an Enhanced PeriOperative Care and Health bundle (EPOCH). Here, we describe the protocol for an open label randomised controlled parallel group superiority trial to test the effect of the EPOCH bundle added to (national) standard care in comparison to standard care alone on the incidence of SSI.

Methods and analysis: EPOCH consists of intraoperative high FiO_2 (0.80), Goal Directed Fluid Therapy, active pre- intra- and postoperative warming, perioperative glucose control and treatment of severe hyperglycemia (>10mmol^{l-1}), and standardised surgical site handling. Patients scheduled for elective abdominal surgery with an incision larger than five centimetres are eligible for inclusion. Participants are randomised daily, 1:1 according to variable block sizes, and stratified per participating centre to either EPOCH added to standard care or standard care only. The primary endpoint will be SSI incidence according to the CDC definition within 30 days as part of routine clinical follow-up. Four additional questionnaires will be sent out over the course of 90 days to capture disability and costs. Other secondary endpoints include anastomotic leakage, incidence of incisional hernia, serious adverse events, hospital readmissions, length of stay and cost effectiveness. Analysis of the primary endpoint will be on an intention-to-treat basis.

Ethics and dissemination: Ethics approval granted by the Amsterdam UMC Medical Ethics Committee (reference 2015_121). Results will be disseminated through peer-reviewed journals and summaries shared with stakeholders.

Registration number: Registered the Dutch Trial Register: NL5572

For peer review only - http://bmjopen.bmj.com/site/about/guidelines.xhtml

ARTICLE SUMMARY

Strength and limitations of this study

- The intervention under investigation is a bundle of readily available, cheap and evidence-based interventions to reduce surgical site infection rates.
- A randomised controlled trial is used to test the effectiveness of the intervention bundle.
- Because the study investigates a bundle of interventions, inference on the attributive effect of each separate intervention of the bundle is challenging.
- Subjects and researchers cannot be blinded, but outcome assessors are unaware of treatment allocation.

For peer review only

For peer review only - http://bmjopen.bmj.com/site/about/guidelines.xhtml

INTRODUCTION

Background and rationale

Surgical site infections (SSI) cause excess morbidity, mortality, prolonged hospital stay and consequently increase costs.[1 2] In high income countries, 9.4% of all patients undergoing gastrointestinal surgery suffer from SSI.[3] All SSI combined add up to the most frequently reported healthcare associated infection on admission.[4]

Approximately 55% of SSI are deemed preventable with evidence-based strategies.[5] However, individual measures alone have not resulted in a clinically relevant SSI reduction.[6-9] In contrast, efforts that have used systematic approaches, or bundles, have been successful to varying degrees.[10-12] The Institute for Healthcare Improvement developed the care-bundle concept as small set of evidence-based interventions to improve predefined clinical outcomes. When interventions are implemented as a bundle, better outcomes are reported compared to implementation of these same interventions individually.[13] Care bundles have been successfully used to reduce central venous catheter-line infection rates, ventilator-associated pneumonia rates and to improve the outcomes of patients presenting with sepsis.[13-16]

A national care bundle for SSI prevention (Postoperative Wound Infection or POWI bundle) was implemented in 2009 in the Netherlands.[17] The bundle comprises of hygiene discipline (measured by operating theatre door movements), timing of antibiotic prophylaxis (between 15 and 60 minutes prior to incision), normothermia (aimed at a core temperature of 36.5°C postoperatively), and the avoidance of preoperative hair removal.[18] However, a clear national reduction in SSI has not been demonstrated since start.[19] Of these interventions, only normothermia is evidence based.[20-23] For the other three interventions, there is either no evidence at all, or the available evidence indicates there is no strong association with SSI.[24-26]

The development of two new guidelines on SSI prevention by the World Health Organisation (WHO) and the Centers for Disease Control and prevention highlighted that perioperative care can be optimised beyond this limited, four-interventions, Dutch POWI bundle.[27-30] Nonetheless these interventions have not been widely implemented yet. Considering current standards of practice, costs and the potential for implementation, we selected from these guidelines a limited set of readily available, cheap and evidence-based interventions that are not part of current national standard perioperative care and formulated an enhanced perioperative care and health program (EPOCH). EPOCH comprises of intraoperative high FiO_2, intraoperative Goal Directed Fluid Therapy (GDFT), active pre- intra- and postoperative warming, glucose control and treatment of hyperglycemia (>10mmol^{l-1}), in diabetic as well as non-diabetic patients, and wound irrigation before closure using an aqueous antiseptic. Intraoperative high FiO_2, GDFT, active perioperative warming are expected to increase tissue perfusion and hence stimulate wound healing.

From a pragmatic perspective, as control to the EPOCH bundle elements, we chose standard care by discretion of the surgeon and anesthesiologist as the comparator to the same standard care, supplemented with EPOCH. EPOCH interventions and contrasting typical standard care are listed in table 1.

Objectives

Our primary objective is to determine if the EPOCH program added to standard care is superior to standard care with respect to the incidence of SSI as defined by the Centers for Disease Control and prevention (CDC)

For peer review only

For peer review only - http://bmjopen.bmj.com/site/about/guidelines.xhtml

within 30 days post-surgery in patients undergoing elective abdominal surgery with abdominal incisions larger than five centimetres. We hypothesise that the EPOCH program is superior to standard care alone, for the prevention of SSI.[31] Secondary objectives are described in table 2.

Trial Design

The EPOCH trial is designed as an open label pragmatic 1:1 randomised controlled parallel-group multicentre superiority trial.

For peer review only

For peer review only - http://bmjopen.bmj.com/site/about/guidelines.xhtml

METHODS AND ANALYSIS

Study setting

The trial will be conducted in a multicentre setting involving academic, top-clinical and general hospitals throughout the Netherlands. A list of all participating centres and anticipated inclusions will be kept up to date with the Dutch central commission on human research (CCMO) on www.toetsingonline.nl (dossier number: NL52796.018.15). All study sites are expected to be able to perform the EPOCH interventions. When needed, the anesthesiology or surgery expert on the Trial Steering Committee will instruct local clinicians before study initiation.

For peer review only

Eligibility criteria

Inclusion criteria:

In order to be eligible to participate in this study, a subject must meet the following criteria:

- Adult patients
- Scheduled for elective abdominal surgery involving an abdominal incision larger than five centimetres (this includes open surgery as well as laparoscopic surgery with incisions larger than five centimetres i.e. for the excision specimen)

Exclusion criteria

Subject meeting any of the following criteria will be excluded from participation in this study:

- The need for emergency surgery
- Scheduled operation concerning a reoperation for complications from recent surgery (within three months after the initial procedure)
- Scheduled operation as part of a two stage surgical procedure and with second procedure possibly scheduled before follow-up of primary endpoint.
- The inability to read or understand informed consent material or study questionnaires
- Participation in another study with interference of study interventions
- Pregnancy

Interventions

The EPOCH intervention bundle and typical standard care are listed in table 1. Beyond this, standard SSI prevention measures typically include preoperative antibiotic prophylaxis when indicated, sterile technique, surgical site preparation and adherence to current national four-interventions SSI prevention (POWI) bundle consisting of hygiene discipline (measured by operating theatre door movements), timing of antibiotic prophylaxis (between 15 and 60 minutes prior to incision), normothermia (aimed at a core temperature of 36.5°C postoperatively), and the avoidance of preoperative hair removal. To avoid withholding good care, surgeons and anesthesiologists can provide what they consider standard care at their discretion. However, it will be agreed that, whatever standard care is at the study initiation, this will not be structurally expanded to EPOCH interventions during the study period. We will aim to recruit study sites for collaboration that have not

included the EPOCH interventions in their standard of care. In 2015, at the time of the study design and initiation, none of the interventions that comprise EPOCH were regularly applied in Dutch hospitals.

Given that this is a pragmatic trial, patient care and comfort are prioritised at all times. Patient requests to cease any of the interventions are immediately honoured. Similarly, when a patient in the control group requests measures that would not have been withheld from them outside of the trial (i.e. warming in case of discomfort from cold) these are also honoured. In case of iodine allergy, only chlorhexidine gluconate will be used for all aspects of surgical site handling. In case of chlorhexidine gluconate allergy, povidone iodine solution will be used. In the absence of allergy for either compound, chlorhexidine gluconate in alcohol is preferred for skin preparation.[32] For wound irrigation, the choice of agent is left to the discretion of the surgeon. In case of active atrial fibrillation, goal directed therapy based on dynamic preload parameters is impossible. Therefore, hypotensive periods after induction are treated with a vasopressive agent (norepinephrine) until a maximum of 0.08 gamma and thereafter with fluids. Beyond this, there are no criteria for discontinuation or modification of the allocated intervention. The interventions included in EPOCH are individually recommended by the WHO and the CDC and are considered safe.[27-29] At all times, the care team is permitted to amend any of the interventions in both groups to guarantee safe anesthesia and surgery.

Participation in trials that intervene with any of the interventions is not permitted. No other restrictions or recommendations are called for due to the pragmatic nature of the trial. Given the size of the study, any co-intervention that may affect the outcome is expected to randomise evenly to both treatment arms.

Table 1: Overview EPOCH interventions and typical standard care

**EPOCH interventions***	**(Expected) Typical standard care**
Intraoperative administration of high FiO2: Patients will be administered a FiO_2 of 80% after intubation during the entire procedure until extubation.	Intraoperative administration of a FiO2 of 30-40%.
Goal directed fluid therapy: Patients will receive hemodynamic therapy based on a goal directed approach. Optimisation is preferably based on dynamic pre-load parameters (pulse pressure variation or systolic pressure variation) derived from arterial catheter measurements, when such catheter is used based on clinical indication. Arterial catheters will not be placed solely for the trial. When no arterial catheter is indicated, a goal directed approach using a non-invasive hemodynamic monitoring system may be used. When both an arterial catheter and a non-invasive hemodynamic monitoring system are not available, hypotensive periods following induction should be compensated for by employing a vasopressive agent (norepinephrine) until a maximum of 0.08 gamma before fluids are administered.	Hemodynamic management based on clinical judgement including amongst other things heart rate, blood pressure and fluid balance.

For peer review only

For peer review only - http://bmjopen.bmj.com/site/about/guidelines.xhtml

Active pre-, intra- and postoperative warming: Patients will be put under a hot air blanket (for example a bair hugger) from arrival at the pre-operative holding, continued after arrival in the OR, until two hours after surgery in the recovery room. Core temperature is aimed at > 36.5 ^{0}C. If necessary additional legwarmers may be applied. Pre-warmed blankets, replaced when cooled down, can be used as an alternative when a hot air blankets are not available.	Active intraoperative warming - but no pre- and postoperative warming -.
Perioperative blood glucose control: All patients (diabetic and non-diabetic) will be subjected to blood glucose control throughout the procedure and on the first and second postoperative day. Blood glucose will be measured every hour during surgery (at least twice during procedures lasting more than two hours), once after surgery in the recovery room, and twice randomly (not fasting) on postoperative days 1 and 2. Blood glucose measurements over 10mmol^{l-1} will be treated with insulin. Success of glucose interventions will be checked.	Glucose control at discretion of the anesthesiologist
Surgical site handling: An alcohol based antiseptic agent (chlorhexidine gluconate will be used for skin preparation before incision. Before wound closure (after closure of the fascia), tissue will be irrigated with an aqueous antiseptic agent (0.35% - 10% aqueous povidone iodine solution or 1% aqueous chlorhexidine gluconate) followed by lavage with normal saline.	No specific requirements for pre-incisional skin preparation agent or wound irrigation before closure
* EPOCH interventions are supported by evidence-based WHO and CDC guideline recommendations	

Randomisation and treatment allocation

Patients are randomised per day, stratified by centre with a 1:1 ratio using variable block sizes to either intervention or control group with an internet based automated assignment system (CASTOR EDC).[33] The trial management team, or local study staff, will perform randomisation for each day and trial site after all potential participants for that day have decided on trial participation. This ensures allocation concealment.

Participation to the trial and treatment allocation is registered in a specific research section of the electronic medical record. Patients are randomised per day to avoid contamination between groups. Every day, an email will be sent to inform everyone involved in execution of the interventions, including anesthesiologists, surgeons, operation assistants, recovery nurses, and anesthesia and ward nurses, of the treatment allocation for the day.

Outcomes

The primary outcome measure is the difference in the proportion of patients developing a SSI between the two treatment arms as defined by the CDC definition within follow-up of 30 days, which will be evaluated from the Dutch National Surgical Complication Registry (LHCR) with parallel assessment through medical chart

For peer review only

For peer review only - http://bmjopen.bmj.com/site/about/guidelines.xhtml

review by the trial management team to account for possible registration effect.[31] All study outcomes, their definitions and data collection methods are listed in table 2. Additionally, in a sub-study, perioperative subcutaneous PtO2 (mmHg), mitochondrial PtO2 (mmHg) and perioperative immune response will also be studied in a smaller population of 48 patients. Further details regarding these measurements and respective analysis is described in appendix 1.

Table 2: Outcomes EPOCH trial

**Primary outcome**		
Outcome	Definition	Method of data collection
The incidence of surgical site infection within 30 days of follow-up after surgery.	SSI defined by the Centers for Disease Control and prevention (CDC)[31]	LHCR registry and medical chart review
**Secondary outcomes**		
The incidence of surgical site infection within 90 days of follow-up after surgery.	SSI defined by the Centers for Disease Control and prevention (CDC)[31]	LHCR registry and medical chart review
The incidence of surgical site infection within 30 days follow-up after surgery.	SSI defined through a post discharge self-assessment questionnaire[34 35]	Survey including a Dutch translation of the Bluebelle Wound Healing questionnaire[34 35]
The incidence of surgical site infection within 30 days follow-up after surgery.	SSI defined by visual characteristics of definition of SSI of the Centers for Disease Control and prevention (CDC)[31]	Survey including self-reported wound photos.
The incidence of anastomotic leakage within 30 days follow-up after surgery.	Anastomotic leakage defined by the international study group for rectal cancer[36]	LHCR registry and medical chart review
The incidence of incisional hernia within year follow-up after surgery.	Incisional hernia defined by the European Hernia Society, as any abdominal wall gap with or without bulge in the area of a postoperative scar perceptible or palpable by clinical examination or imaging[37 38]	LHCR registry and medical chart review

For peer review only

For peer review only - http://bmjopen.bmj.com/site/about/guidelines.xhtml

All-cause mortality within one year follow up after surgery.		Data retrieved from Dutch national registry (Basisregistratie Personen, BRP)
Serious adverse events (SAE) within 30 days follow up after surgery.	SAE defined as any event that results in death, is life threatening (at the time of the event), requires prolonged hospitalisation or re-hospitalisation, results in persistent or significant disability or incapacity, is a congenital anomaly or birth defect, or any other event that may jeopardise the subject or require an intervention to prevent one of the outcomes listed before.	LHCR registry and medical chart review
Length of hospital stay	Number of days between surgery and discharge, and the number of days of potential readmissions.	Data retrieved from medical chart
Related costs	Direct, indirect, medical and non-medical costs. Costs per quality adjusted life year (QALY) constitute the primary health economic outcome.	Hospital health care utilisation retrieved from health informatics system. Survey on out-of-hospital healthcare utilisation and productivity losses from surveys containing the institute for Medical Technology Assessment's (iMTA) Medical Consumption Questionnaire (iMCQ) adjusted to the study setting and the iMTA Productivity Cost Questionnaire (iPCQ).[39] A survey with out-of-pocket expenses will be added to the survey
Hospital readmissions	Yes or no	Data extraction from medical chart
Health and disability		Gathered with the WHO disability assessment schedule (WHODAS) 2.0 at 30, 60 and 90 days[40]
Health utility and QALY		Gathered with the EuroQol EQ-5D-3L at 30, 60 and 90 days[41]
Additional secondary outcome, depending on additional funding		
All-cause mortality within five years follow up after surgery.		Data retrieved from Dutch national registry (Basisregistratie Personen, BRP)

For peer review only - http://bmjopen.bmj.com/site/about/guidelines.xhtml

Participant timeline

A schematic diagram of the participant timeline is depicted in figure 1.

Sample size

Local SSI surveillance data (Unpublished: Amsterdam UMC, Location AMC; 2008-2012) show a 9.1% SSI risk with standard care. A maximum proportion of approximately 55% of SSI is deemed preventable by application of evidence-based strategies.[5] Accounting for a limited degree of contamination of intervention between groups and other factors contributing to SSI, not affected by the bundle, we deem a clinically relevant relative risk reduction of 30% reasonable. This 30% relative reduction represents a 9.1% SSI risk with standard care and a 6.37% SSI risk in the intervention group. A sample size of 3000 evaluable patients, 1500 in each arm, is sufficient to detect this 30% difference in proportion of SSI between the two groups using a two-tailed Z-test with 80% power and a 5% level of significance.

A report by the European Centre of Disease prevention and Control (ECDC) indicates SSI incidence in the Netherlands to be considerably higher than 9.1% we used for our sample size estimation. We chose to use the more conservative value to prevent underestimation of the sample size. In addition, the randomisation per day per centre enables within-centre comparison, which is not yet accounted for in the current sample size estimation. Accounting for this may increase power or lower the number of patients required to attain the same power. Therefore, the independent statistician of the Data Safety Monitoring Board (DSMB) will perform a sample size recalculation after inclusion of the first 1500 patients. This recalculation will account for within-centre effects and includes evaluation of the pre-study sample size assumption of the SSI incidence in the control group. As the primary outcome falls within regular follow-up visits, no missing data is expected. Therefore, the sample size is not corrected for missing data.

Recruitment

At the study initiation, 11 hospitals committed to trial participation with a combined expected enrolment of 5419 participants over 33 months representing nearly 50% oversampling. Inclusion criteria are broad and straightforward and, given the low risk and potential benefit of the interventions, willingness to participate is expected to be high. No financial incentives are provided to investigators or participants.

Blinding

Due to the nature of the interventions, trial participants and providers will not be blinded. Outcome assessors, ward doctors responsible for LHCR registration, are not involved in execution of the interventions and will be kept unaware of the treatment allocation.

Data collection methods

All data will be collected and stored in a web-based electronic data capture system (CASTOR EDC).[33] Clinical data with regard to safety and efficacy outcomes will be collected manually by the trial management team during routine trial site visits using a standardised electronic case record form (eCRF). Baseline and clinical data

For peer review only - http://bmjopen.bmj.com/site/about/guidelines.xhtml

For peer review only

will be collected through a detailed export directly from the electronic medical record to avoid human error. After data validation, data will be imported into the eCRF. Any modification to entered data is password secured and can only be processed with a corresponding explanation for the audit trail.

Survey data (from surgeon, anesthesiologist and patients) will be collected via an eCRF on the EDC. When email is not available, printed CRFs via surface mail will be used. A trial nurse will process surface mail responses in the eCRF. The trial management team will track survey responses, send reminders and conduct follow up phone calls when necessary.

To monitor intervention adherence and potential contamination between groups, a postoperative survey is sent to surgeons and anesthesiologists on execution of the EPOCH interventions and other interventions that may affect the outcome. In addition, relevant process measures (e.g. administered FiO_2, measured temperature perioperative) are extracted from clinical data. For goal directed fluid therapy and surgical site preparation we rely fully on the self-assessment checklist.

For the primary outcome, SSI is prospectively evaluated according to the CDC criteria.[31] SSI are registered by treating physicians as part of routine postoperative care and outpatient follow-up in accordance with the standardised Dutch National Surgical Complication Registration (LHCR) up to 30 days after surgery. Physicians involved are expected to be familiar with the CDC criteria. Physicians at the surgical ward are typically responsible for SSI registration but are not involved in the operative procedure. We do not expect operating surgeons to actively manipulate this registration and no additional measures are taken to formally exclude them from outcome assessment. Outside routine postoperative care, no additional follow-up visits are planned specifically for the trial. Timing of these follow-up visits vary and occur within 30 days after surgery. Patients are expected to return to the hospital whenever complications occur. If they do not, we will assume they did not develop a SSI. The research physicians from the trial management team will review all medical charts to account for a potential registration effect. Data collection on secondary outcomes is included in table 2.

Retention and withdrawal

The burden of participation in the study will be minimised. With the exception of two glucose measurements postoperatively and active pre- and postoperative warming, the interventions take place while the patient is under anesthesia. No active adherence from the patient is required and non-adherence from patients is therefore not expected. No additional visits to the hospital are required. Respondents are asked to complete four questionnaires over a period of 90 days. The trial management team will provide support and actively remind respondents via email, surface mail and phone calls. No (financial) incentives will be provided. Whenever a subject does decide to withdraw, all data collected up until withdrawal will be used.

The investigator can decide to withdraw a subject from the study for urgent medical reasons or when intraoperative findings indicate that inclusion criteria are not met anymore (e.g. diagnostic laparoscopy, change in operative plan resulting in trans-anal approach), rendering the patient ineligible for the study. Patients withdrawn from the study are replaced.

For peer review only - http://bmjopen.bmj.com/site/about/guidelines.xhtml

Data management

Subjects are coded by a numeric randomisation code. Local investigators are responsible for the subject identification log. Data are stored digitally and will be kept by the project leader for 15 years after the inclusion of the last patient.

Statistical methods

All outcomes will be analysed according to the intention-to-treat principle. Binary outcomes will be analysed using log binomial regression to estimate relative risks. If this regression fails to converge, logistic regression will be performed using odds ratio and corresponding confidence interval for statistical interpretation and recalculated relative risk for point estimate interpretation. Continuous outcome data will be analysed using linear regression. Quality of life data will be analysed as repeated measurement using a linear mixed model. In all analyses, statistical uncertainties are expressed in 95% two-sided confidence intervals. P-values of <0.05 will indicate statistical significance.

Randomisation daily per trial site enables within-centre comparison of treatments and increases power. A sensitivity analysis of the primary outcome will be conducted that accounts for within centre comparison of treatments. When the hypothesis test of this analysis diverges from the primary analysis, all further analysis will also be conducted using this effect estimate. All outcomes will also be analysed according to the per protocol population after adjustment for confounding due to incomplete adherence to the assigned treatments or use of off-protocol concomitant therapies. Variables will be considered for adjustment based on Tyler van de Weele's principles for confounder selection and include preoperative body mass index (BMI), (insulin dependent) diabetes mellitus (DM), cardiovascular heart diseases other than hypertension, chronic obstructive pulmonary disease (COPD) and any other variables that meet these criteria and pass statistical variable selection.[42] Procedure duration is considered an important proxy for complexity of procedure and independent of the interventions comprising EPOCH. Therefore, it will also be considered for adjustment despite being measured during the exposure. In an additional analysis, this method will also be applied to each individual intervention of the EPOCH bundle, the POWI bundle, and other promising interventions outside the bundle under investigation to assess the attributive effect of each intervention separately.

The intention-to-treat population will comprise of all evaluable randomised patients regardless of adherence of treatment allocation. The per protocol population will comprise of all patients who were treated per protocol according to the postoperative adherence surveys and process measures, and all patients in the control group who were not treated with these interventions according to the postoperative adherence surveys and process measures. The safety analysis will be conducted on all patients exposed to the interventions, regardless of evaluable primary outcome. Missing data will be handled by multiple imputation by chained equation when the required assumptions apply. A more detailed description of statistical methods and analyses is described in the statistical analysis plan (SAP). The EPOCH SAP will be published separate from the EPOCH Protocol timely before analysis of the data.

Monitoring

For peer review only - http://bmjopen.bmj.com/site/about/guidelines.xhtml

An independent data safety and monitoring board (DSMB) safeguards the interests of trial participants and assesses the safety of the interventions during the trial. The DSMB consists of a statistician, an anesthesiologist and an infectious disease specialist. The DSMB will regularly monitor safety data, recruitment and loss to follow up and make recommendations about continuation of the trial. After every 50 new serious adverse events (any type) or 10 new deaths, the DSMB will re-evaluate. If required, monitoring can also be on ad hoc basis. There will be no interim analysis of the treatment effect. At its own discretion, the DSMB may advice to stop the study prematurely based on its findings. This advice is not bound to specific guidelines other than that it should serve the interest of the trial participants. If the sponsor decides not to (fully) implement the advice, the sponsor will inform the MEC.

Harms

All Serious Adverse Events (SAE) that occur within 30 days after surgery are recorded and reported to the DSMB irrespective of perceived relation to the intervention. SAE are defined as any event within 30 days of the intervention that results in death, is life threatening (at the time of the event), requires prolonged hospitalisation or re-hospitalisation, results in persistent or significant disability or incapacity, is a congenital anomaly or birth defect, or any other event that may jeopardise the subject or require an intervention to prevent one of the outcomes listed before based on appropriate medical judgement. SAE will be followed up until they have abated, or until a stable situation has been reached.

All physicians involved in the EPOCH study are instructed to report serious adverse events to the study coordinator. Mortality has to be reported within 48 hours after the occurrence. In addition to spontaneous reporting, the trial management team will review medical charts within 30-day intervals. The MEC will receive a biannual line listing of all reported SAE.

In accordance with the Dutch human research law (WMO), the sponsor will suspend the study if there is sufficient ground that continuation of the study will jeopardise health or safety of study participants. The sponsor will notify the MEC without undue delay of a temporary halt including the reason for such an action. The study will be suspended pending a further decision by the MEC. When appropriate, the investigator will ensure that all subjects will be informed.

Auditing

The Amsterdam UMC clinical research unit may decide to conduct audits and monitoring visits at random, independently of trial coordinator and sponsor. Due to minimal risks of the interventions there are no scheduled monitoring visits.

Patient and Public involvement

The Dutch patient and healthcare consumer federation (NPCF) endorsed the study and provided input on the study proposal.

Rationale for the study design

For peer review only - http://bmjopen.bmj.com/site/about/guidelines.xhtml

An alternative to the present study design of 1:1 parallel group randomisation per day per trial site is cluster randomisation. Cluster randomisation can overcome the risk of contamination by randomising study participants by trial site. However, disadvantages of cluster randomisation include the risk of residual confounding due to imbalanced distribution of trial site characteristics (like baseline level of SSI prevention), and the need for a larger sample size to ensure equivalent statistical power.[43-45] This may occur despite large numbers of participants and clusters.[46] Substantial contamination can be tolerated within an individual randomised trial before a cluster design is better in terms of total sample size. By randomising per trial site per day, every patient treated at that particular site by that particular team will receive the same care during the entire day. At the individual patient level, the allocation remains completely random. This design foregoes the weaknesses of full cluster randomisation, while minimizing the risk of contamination of usual care by the EPOCH enhanced care. Furthermore, contamination is taken in to account in the sample size calculation.

ETHICS AND DISSEMINATION

Ethics

This study will be conducted in accordance with the Declaration of Helsinki (64th version, October 2013) and the laws governing human research in the Netherlands (Wet Medisch-wetenschappelijk Onderzoek met mensen – WMO and Best Clinical Practice (BCP)) and the guidelines of the Central Committee for Research involving Human Subjects (Centrale Commissie Mensgebonden Onderzoek - CCMO). The Amsterdam UMC MEC has approved this trial (reference 2015_121).

All intended protocol modifications with implications beyond administrative changes will be submitted to the MEC and only effectuated after formal approval for the change. Strictly administrative amendments will be notified to the MEC. Local principle investigators will be notified of all amendments. Participants will be notified if necessary. Current version of the protocol is version 10, 17-12-2019. All amendments are described in table 3.

Table 3. Revision chronology EPOCH study protocol

Revision chronology EPOCH study protocol		
Version no.	Date	Main reasons for change
1.	28-03-2015	NA, first submission to MEC
2.	14-07-2015	Modifications requested by MEC d.d. 09-05-2015
3.	28-07-2015	The use of sutures, instead of staples, for skin closure is no longer required in the intervention group. Evidence emerged before the study start that rebuked earlier perceptions on the risk of SSI after the use of staples.
4.	12-11-2015	Administrative modification requested by MEC d.d. 12-11-2015 to comply with renewed law for human research.
5.	22-02-2016	Addition of patient generated wound photos to data collection.

For peer review only

6.	05-07-2018	Addition of a post discharge SSI surveillance checklist at 30 days postoperatively to outcome data for all trial participants and tissue oxygen tension measurement and immunologic analysis to investigate mechanism of potential effect of the interventions for a limited subset of trial participants. Modification of SAE reporting from individual event reports to a list of all events every six months to alleviate administrative pressure.
7.	24-01-2019	Modification of technique for tissue oxygenation measurement and immunologic analysis before first measurement was conducted.
8.	26-05-2019	Modification of DSMB charter at request of the DSMB and modifications of the wording of inclusion criteria and replacement of non-evaluable patients to better reflect practice, and practical modifications to ease administrative burden and logistics.
9.	23-08-2019	Modification of sample size and inclusion criteria for additional measurements in limited subset of trial participants.
10	17-12-2019	Modification of exclusion criteria

Confidentiality

Informed consent forms will be securely stored at local study sites in locked cabinets with limited access. All data will be linked to an anonymised study number. The local investigators are responsible for the subject identification log. All local databases will be secured by password. All other information regarding the trial will be stored securely in a local trial master file. Data collected during this trial will only be used for the objective of this study described in the informed consent unless additional consent of the participant is retrieved. The informed consent form is enclosed in appendix 2. The trial steering committee and study coordinator will control access of the final data set.

Ancillary and post-trial care

No provisions for post-trial care are made. MEC has waived the obligation to take out a special insurance for participants because there are no risks associated with participation to this study.

Dissemination

No arrangements have been made concerning public disclosure. The study is registered in the Dutch trial register as Trial NL5572 (https://www.trialregister.nl/trial/5572). An overview of the WHO trial registration data set is described in table 4. The trial results will be submitted to a peer-reviewed journal regardless of the outcome and made open access available in accordance with the Netherlands Organization for Health Research and innovation (ZonMW) policy. Co-authorship will be based on the international committee of medical journal editors' guidelines. Contributors that do not fulfil these criteria will be listed as collaborator. The order of authors will be based on scientific input.

For peer review only

For peer review only - http://bmjopen.bmj.com/site/about/guidelines.xhtml

403 The study protocol and statistical analysis plan will be published after half of the planned sample size, 1500 evaluable participants, have been enrolled and before any of the outcome data will be available. Participant level data and statistical code will be made available on request after all trials results have been published and a data sharing agreement has been establishment.

For peer review only

For peer review only - http://bmjopen.bmj.com/site/about/guidelines.xhtml

Table 4: WHO trial registration data set

**WHO trial registration data set**	
Primary registry and trial identifying number	Trial NL5572 (NTR5694)
Date of registration in primary registry	2016-03-03
Secondary identifying numbers	NA
Source(s) of monetary or material support	ZonMW Dutch Healthcare efficacy program<br>Innovation fund Dutch healthcare insurers<br>Ethicon, Johnson & Johnson
Primary sponsor	Amsterdam University Medical Centre, Location AMC
Secondary sponsor(s)	NA
Contact for public queries	m.a.boermeester@amsterdamumc.nl
Contact for scientific queries	m.a.boermeester@amsterdamumc.nl
Public title	Enhanced care around surgery for the prevention of surgical site infections
Scientific title	Enhanced perioperative care for the prevention of surgical site infections, A Pragmatic, randomised controlled parallel-group multicentre superiority trial
Countries of recruitment	The Netherlands
Health condition(s) or problem(s) studied	Surgical Site infection
Intervention(s)	Intervention:<br>An enhanced perioperative care program added to standard care consisting of:<br>- Supplemental oxygen (FiO_2 0.80)<br>- Pre, intra, and postoperative warming<br>- Goal directed fluid therapy<br>- Perioperative glucose control < 10mmol^{l-1}<br>- Surgical wound irrigation<br><br>Control:<br>Standard care at discretion of treating physician
Key inclusion and exclusion criteria	Inclusion criteria:<br>Adult patients undergoing elective open or laparoscopic abdominal surgery with an incision larger than 5 cm.<br><br>Exclusion criteria:<br>- The need for emergency surgery

For peer review only

	- Scheduled operation concerning a reoperation for complications from recent surgery (within three months after the initial procedure)<br>- Scheduled operation as part of a two staged surgical procedure and second procedure possibly scheduled before follow-up of primary endpoint<br>- The inability to read or understand informed consent material or study questionnaires<br>- Participation in another study with interference of study interventions or outcomes<br>- Pregnancy
Study type	A pragmatic, randomised controlled parallel-group multicentre superiority trial
Date of first enrolment	2016-03-01
Target sample size	3000
Recruitment status	Recruiting
Primary outcome(s)	Incidence of surgical site infections within 30 days evaluated from the Dutch National Surgical Complication Registry (LHCR) with parallel assessment by the CDC definition through medical chart reviews.
Key secondary outcomes	- SSI incidence evaluated at 30 days and three months follow-up by the CDC definition through medical chart review<br>- Readmissions rate within 30 days follow-up through LHCR registration and medical chart review<br>- All-cause mortality within one and five years after surgery<br>- WHO disability assessment schedule 2.0 by self-administration through online/paper-form questionnaires at postop day 30, 60 and 90<br>- (In)direct medical and non-medical costs, quality adjusted life years (QALY)<br>- Incidence of anastomotic leakage at 30 days follow-up through LHCR registration and medical chart review<br>- Incidence of incisional hernia by medical chart review (diagnosed by either physical examination and/or ultrasonography or CT) one year after surgery

For peer review only

Contribution

MB and MWH conceived of the study. MB, MWH, MD and SJ designed the study. MD provided statistical and methodological expertise. MB provided expertise from the surgical perspective and MW provided expertise from the anesthesiological perspective. MB, MWH, MD, SJ, QB, LP, WB, NW, and JS contributed to the study protocol. All local investigators (BL, JH, JPH, DS, OG, ER, EK, AD, LL, TS, WD, JK, CR, LO, PD) provided essential critical input on the study protocol. MB guided and supervised SJ with the grant application, obtaining medical ethics approval, and writing of the study protocol. SJ, QB, LP, WB, NW and JS will help with implementation and execution of the trial. SJ and NW will conduct the statistical analysis supervised by MD. SJ, NW and QB will draft the final manuscript under supervision of MB, MD and MWH.

Funding

The EPOCH trial is funded by the Netherlands Organization for Health Research and innovation (ZonMw; project 843002606) with 10% compulsory co-financing provided by the Innovation Fund of Dutch healthcare insurers and by Ethicon (Johnson & Johnson).

Roles and responsibilities

Roles and responsibilities are described in table 5.

Table 5: roles and responsibilities in the EPOCH trial

Roles and responsibilities in the EPOCH trial	
**Role**	**Details and responsibilities**
Trial sponsor	Trial sponsor: Amsterdam University Medical Centre, Location AMC, Department of Surgery and Department of Anesthesiology<br>Principle Investigators: Prof. dr. M.A. Boermeester and prof. dr. dr. M.W. Hollmann<br>Address: Meibergdreef 9, 1105 AZ Amsterdam, The Netherlands<br>Email address: m.a.boermeester@amsterdamumc.nl, m.w.hollmann@amsterdamumc.nl<br>The sponsor conceived of the study and chairs the trial steering committee. The sponsor is not responsible for data collection, but is responsible for trial management, analysis, interpretations, writing of the report and decision to submit the report for publication. The sponsor does not provide any funding.
Principal investigator	The principal investigators (M.A. Boermeester and M.W. Hollmann) are responsible for the overall conduct of the trial.
Local investigators	The local investigator team consists of an anesthesiologist and a surgeon at each trial site. The local investigators are responsible for local trial execution.
Trial Steering Committee (TSC)	The Trials Steering Committee (TSC) consists of the principal investigators (M.A. Boermeester, Professor of Surgery and Clinical epidemiologist; M.W. Hollmann,

For peer review only - http://bmjopen.bmj.com/site/about/guidelines.xhtml

	Professor of Anesthesiology; M.G. Dijkgraaf, Professor of Health Technology Assessment) and a research physician (S.W. de Jonge, Clinical epidemiologist). The TSC is responsible for design and overall conduct of the trial. This includes overseeing execution of the protocol, preparation of the protocol and potential amendments to the protocol, the statistical analysis plan, yearly progress reports to Amsterdam UMC medical ethics committee (MEC), safety reports to the Data Safety Monitoring Board (DSMB), and the final report.
Study Coordinator	The study coordinator (Q.J.J. Bolding & N. Wolfhagen) will coordinate the overall execution of the trial. This includes communication with local investigators, trial sites, if necessary included patients, and physicians executing the interventions. The study coordinator also oversees follow-up, data collection, data management and recruitment and start-up of new trials sites.
Trial Management Team	The trial management team (Q.J.J. Boldingh, N. Wolfhagen, S.W. de Jonge, W.J. Bom, L.M. Posthuma, and J.C.G. Scheijmans) assists the study coordinator in the overall execution and if necessary assists local investigators with execution. The trial management team also performs screening of potential participants, recruitment of participants, randomisation and allocation of the intervention.
Data Safety Monitoring Board (DSMB)	The DSMB safeguards the interests of trial participants and assesses the safety of the interventions during the trial. In addition, the DSMB performs the recalculation of the sample size. The DSMB consists of two clinicians with relevant subject matter knowledge (J.T.M van der Meer, infectious disease specialist & M. Klimek, anesthesiologist) and a statistician (P.M. Bossuyt). J.T.M. van der Meer chairs the DSMB. The DSMB reports to the trial sponsor.
Independent physician	The independent physician (T. Schepers, trauma surgeon) represents interests of trial participants and mediates conflicts between trial participants and investigators if necessary.

Competing interests

All authors declare no competing interests.

For peer review only

References

1. de Lissovoy G, Fraeman K, Hutchins V, et al. Surgical site infection: incidence and impact on hospital utilization and treatment costs. *American journal of infection control* 2009;37(5):387-97. doi: 10.1016/j.ajic.2008.12.010
2. O'Brien WJ, Gupta K, Itani KMF. Association of Postoperative Infection With Risk of Long-term Infection and Mortality. *JAMA Surg* 2019:1-8. doi: 10.1001/jamasurg.2019.4539 [published Online First: 2019/11/07]
3. GlobalSurg C. Surgical site infection after gastrointestinal surgery in high-income, middle-income, and low-income countries: a prospective, international, multicentre cohort study. *Lancet Infect Dis* 2018;18(5):516-25. doi: 10.1016/S1473-3099(18)30101-4 [published Online First: 2018/02/18]
4. Suetens C, Latour K, Karki T, et al. Prevalence of healthcare-associated infections, estimated incidence and composite antimicrobial resistance index in acute care hospitals and long-term care facilities: results from two European point prevalence surveys, 2016 to 2017. *Euro Surveill* 2018;23(46) doi: 10.2807/1560-7917.ES.2018.23.46.1800516 [published Online First: 2018/11/22]
5. Umscheid CA, Mitchell MD, Doshi JA, et al. Estimating the proportion of healthcare-associated infections that are reasonably preventable and the related mortality and costs. *Infect Control Hosp Epidemiol* 2011;32(2):101-14. doi: 10.1086/657912
6. Nguyen N, Yegiyants S, Kaloostian C, et al. The Surgical Care Improvement project (SCIP) initiative to reduce infection in elective colorectal surgery: which performance measures affect outcome? *AmSurg* 2008;74(10):1012-16.
7. Stulberg JJ, Delaney CP, Neuhauser DV, et al. Adherence to surgical care improvement project measures and the association with postoperative infections. *JAMA* 2010;303(24):2479-85. doi: 10.1001/jama.2010.841 [published Online First: 2010/06/24]
8. Awad SS. Adherence to surgical care improvement project measures and post-operative surgical site infections. *Surg Infect (Larchmt)* 2012;13(4):234-7. doi: 10.1089/sur.2012.131 [published Online First: 2012/08/24]
9. Diener MK, Knebel P, Kieser M, et al. Effectiveness of triclosan-coated PDS Plus versus uncoated PDS II sutures for prevention of surgical site infection after abdominal wall closure: the randomised controlled PROUD trial. *Lancet* 2014;384(9938):142-52. doi: 10.1016/S0140-6736(14)60238-5 [published Online First: 2014/04/11]
10. Bull A, Wilson J, Worth LJ, et al. A bundle of care to reduce colorectal surgical infections: an Australian experience. *J Hosp Infect* 2011;78(4):297-301. doi: 10.1016/j.jhin.2011.03.029 [published Online First: 2011/06/15]
11. Cima R, Dankbar E, Lovely J, et al. Colorectal surgery surgical site infection reduction program: a national surgical quality improvement program--driven multidisciplinary single-institution experience. *J Am Coll Surg* 2013;216(1):23-33. doi: 10.1016/j.jamcollsurg.2012.09.009 [published Online First: 2012/11/07]
12. Keenan JE, Speicher PJ, Thacker JK, et al. The preventive surgical site infection bundle in colorectal surgery: an effective approach to surgical site infection reduction and health care cost savings. *JAMA Surg* 2014;149(10):1045-52. doi: 10.1001/jamasurg.2014.346 [published Online First: 2014/08/28]
13. Resar R, Griffin FA, Haraden C, et al. Using Care Bundles to Improve Health Care Quality. IHI Innovation Series white paper. Cambridge, Massachusetts: Institute for Healthcare Improvement, 2012.

For peer review only

For peer review only - http://bmjopen.bmj.com/site/about/guidelines.xhtml

14. Dellinger RP, Levy MM, Rhodes A, et al. Surviving sepsis campaign: international guidelines for management of severe sepsis and septic shock: 2012. *Crit Care Med* 2013;41(2):580-637. doi: 10.1097/CCM.0b013e31827e83af [published Online First: 2013/01/29]

15. Pronovost P, Needham D, Berenholtz S, et al. An intervention to decrease catheter-related bloodstream infections in the ICU. *N Engl J Med* 2006;355(26):2725-32. doi: 10.1056/NEJMoa061115 [published Online First: 2006/12/29]

16. Morris AC, Hay AW, Swann DG, et al. Reducing ventilator-associated pneumonia in intensive care: impact of implementing a care bundle. *Crit Care Med* 2011;39(10):2218-24. doi: 10.1097/CCM.0b013e3182227d52 [published Online First: 2011/06/15]

17. veiligheidsprogramma V. Praktijkgids 'Voorkomen van wondinfecties na een operatie', 2009.

18. van den Broek P, Bekkers R, Bonten M, et al. Voorkomen van wondinfecties na een operatie: VMSzorg; 2009 [Available from: http://www.vmszorg.nl/_library/5546/web_2009.0111_praktijkgids_powi.pdf.

19. RIVM. PREZIES Resultaten bijeenkomst POWI, 2013.

20. Melling AC, Ali B, Scott EM, et al. Effects of preoperative warming on the incidence of wound infection after clean surgery: A randomised controlled trial. *Lancet* 2001;358(9285):876-80.

21. Kurz A, Sessler DI, Lenhardt R. Perioperative normothermia to reduce the incidence of surgical-wound infection and shorten hospitalization. Study of Wound Infection and Temperature Group. *N Engl J Med* 1996;334(19):1209-15. doi: 10.1056/nejm199605093341901 [published Online First: 1996/05/09]

22. Wong PF, Kumar S, Bohra A, et al. Randomized clinical trial of perioperative systemic warming in major elective abdominal surgery. *The British journal of surgery* 2007;94(4):421-6. doi: 10.1002/bjs.5631 [published Online First: 2007/03/24]

23. Mahoney CB, Odom J. Maintaining intraoperative normothermia: a meta-analysis of outcomes with costs. *AANA J* 1999;67(2):155-63. [published Online First: 1999/09/17]

24. Hawn MT, Richman JS, Vick CC, et al. Timing of surgical antibiotic prophylaxis and the risk of surgical site infection. *JAMA Surg* 2013;148(7):649-57. doi: 1669977 [pii];10.1001/jamasurg.2013.134 [doi]

25. Tanner J, Norrie P, Melen K. Preoperative hair removal to reduce surgical site infection. *Cochrane Database Syst Rev* 2011(11):CD004122. doi: 10.1002/14651858.CD004122.pub4

26. de Jonge SW, Boldingh QJJ, Koch AH, et al. Timing of Preoperative Antibiotic Prophylaxis and Surgical Site Infection: TAPAS, An Observational Cohort Study. *Ann Surg* 2019 doi: 10.1097/SLA.0000000000003634 [published Online First: 2019/10/31]

27. Berrios-Torres SI, Umscheid CA, Bratzler DW, et al. Centers for Disease Control and Prevention Guideline for the Prevention of Surgical Site Infection, 2017. *JAMA Surg* 2017;152(8):784-91. doi: 10.1001/jamasurg.2017.0904

28. Allegranzi B, Bischoff P, de Jonge S, et al. New WHO recommendations on preoperative measures for surgical site infection prevention: an evidence-based global perspective. *The Lancet Infectious Diseases* 2016;16(12):e276-e87. doi: 10.1016/s1473-3099(16)30398-x

29. Allegranzi B, Zayed B, Bischoff P, et al. New WHO recommendations on intraoperative and postoperative measures for surgical site infection prevention: an evidence-

For peer review only

based global perspective. *The Lancet Infectious Diseases* 2016;16(12):e288-e303. doi: 10.1016/s1473-3099(16)30402-9

30. Organization WH. Global Guidelines for the Prevention of Surgical Site Infection. Global Guidelines for the Prevention of Surgical Site Infection. Geneva2016.
31. Horan TC, Gaynes RP, Martone WJ, et al. CDC definitions of nosocomial surgical site infections, 1992: a modification of CDC definitions of surgical wound infections. *American journal of infection control* 1992;20:271-74. doi: 10.1016/S0196-6553(05)80201-9
32. WHO. WHO Surgical Site Infection Prevention Guidelines; Webappendix 8; Summary of a systematic literature review on surgical site preparation [Available from: https://www.who.int/gpsc/appendix8.pdf accessed 14-04-2020.
33. Castor EDC. Castor Electronic Data Capture 2019 [27 Aug. 2019]. Available from: https://castoredc.com accessed August 28, 2019.
34. Macefield RC, Reeves BC, Milne TK, et al. Development of a single, practical measure of surgical site infection (SSI) for patient report or observer completion. *J Infect Prev* 2017;18(4):170-79. doi: 10.1177/1757177416689724 [published Online First: 2017/10/11]
35. Bluebelle Study G. Validation of the Bluebelle Wound Healing Questionnaire for assessment of surgical-site infection in closed primary wounds after hospital discharge. *The British journal of surgery* 2019;106(3):226-35. doi: 10.1002/bjs.11008 [published Online First: 2018/12/18]
36. Rahbari NN, Weitz J, Hohenberger W, et al. Definition and grading of anastomotic leakage following anterior resection of the rectum: a proposal by the International Study Group of Rectal Cancer. *Surgery* 2010;147(3):339-51. doi: 10.1016/j.surg.2009.10.012
37. Muysoms FE, Miserez M, Berrevoet F, et al. Classification of primary and incisional abdominal wall hernias. *Hernia* 2009;13(4):407-14. doi: 10.1007/s10029-009-0518-x [published Online First: 2009/06/06]
38. Korenkov M, Paul A, Sauerland S, et al. Classification and surgical treatment of incisional hernia. Results of an experts' meeting. *Langenbecks Arch Surg* 2001;386(1):65-73. doi: 10.1007/s004230000182 [published Online First: 2001/06/19]
39. Hakkaart-van Roijen L, van der Linden N, Bouwmans C, et al. Kostenhandleiding: Methodologie van kostenonderzoek en referentieprijzen voor economische evaluaties in de gezondheidszorg, geactualiseerde versie 2015: Institute for Medical Technology Assessment, Erasmus Universiteit Rotterdam.
40. Organization WH. WHO Disability Assessment Schedule 2.0 (WHODAS 2.0) [Available from: https://www.who.int/classifications/icf/more_whodas/en/November 6th 2019.
41. EuroQol. EQ-5D-3L [Available from: https://euroqol.org/eq-5d-instruments/eq-5d-3l-about/ accessed November 6th 2019.
42. VanderWeele TJ. Principles of confounder selection. *Eur J Epidemiol* 2019;34(3):211-19. doi: 10.1007/s10654-019-00494-6 [published Online First: 2019/03/07]
43. Campbell MK, Piaggio G, Elbourne DR, et al. Consort 2010 statement: extension to cluster randomised trials. *BMJ (Clinical research ed)* 2012;345:e5661. doi: 10.1136/bmj.e5661 [published Online First: 2012/09/07]
44. Carter B. Cluster size variability and imbalance in cluster randomized controlled trials. *Stat Med* 2010;29(29):2984-93. doi: 10.1002/sim.4050 [published Online First: 2010/10/22]

For peer review only - http://bmjopen.bmj.com/site/about/guidelines.xhtml

45. Torgerson DJ. Contamination in trials: is cluster randomisation the answer? *BMJ (Clinical research ed)* 2001;322(7282):355-7. doi: 10.1136/bmj.322.7282.355 [published Online First: 2001/02/13]

46. Alexander FE, Anderson TJ, Brown HK, et al. 14 years of follow-up from the Edinburgh randomised trial of breast-cancer screening. *Lancet (London, England)* 1999;353(9168):1903-8. doi: 10.1016/s0140-6736(98)07413-3 [published Online First: 1999/06/17]

Figure 1: Schedule of enrolment, interventions, and assessments.

For peer review only

	STUDY PERIOD									
	**Enrolment**	**Allocation**	**Post-allocation**							
**TIMEPOINT**	**Up to -1 day**	**-1 day**	**0**	**1 day**	**2 days**	**10 days**	**30 days**	**60 days**	**90 days**	**1 year**
**ENROLMENT:**										
Eligibility screen	X			X						
Informed consent	X									
Allocation		X								
**INTERVENTIONS:**										
Surgical procedure standard care			X							
Surgical procedure standard care supplemented with EPOCH			X	X	X					
**ASSESSMENTS:**										
Baseline characteristics			X							
Perioperative characteristics			X							
Adherence survey surgeon			X							
Adherence survey anesthesiologist			X							
Serious Adverse Event			As needed throughout protocol							
SSI by medical chart review							X		X	
Incidence SSI by survey							X			
Anastomotic leakage							X		X	
Incisional hernia							X			X
EQ-5D-3L						X	X	X	X	
Wound photo						X	X			
WHODAS 2.0							X	X	X	
IMTA-MCQ									X	
IMTA-PCQ									X	

For peer review only

Appendix 1. Additional measurements

Background

Studies have shown a negative correlation between tissue oxygen tension and SSI.[1-4] Conversely, improving tissue oxygen tension has shown reduce the risk of SSI.[5] Several interventions, thought to improve tissue oxygen tension, including hyperoxygenation, active warming and goal directed fluid therapy, have shown to help reduce the risk of SSI.[5-7] However, the evidence for these interventions is based on clinical outcomes and the precise mechanism of effect is not entirely clear. In vitro experiments have indicated that optimized oxygenation may enhance immunologic response,[8] but only one study on these interventions actually measured oxygen tension as a process measure.[5] The study, found a marked increase in oxygen tension and over 50% reduction in SSI.[5] Various investigators have tried to reproduce these results with varying success.[9] Since the early positive results, much has changed in perioperative care with considerable implications for cardiovascular and respiratory physiology. Open procedures with aggressive hydration regimens have made room for laparoscopic surgery with enhanced recovery programs and restricted fluid regimens. These changes could modify the effect on oxygen tension and SSI. The interventions thought to improve oxygen tension, Hyperoxygenation, active warming, and goal directed fluid therapy, are all components of the EPOCH bundle. This poses an extraordinary opportunity to investigate the effect of these interventions combined on tissue oxygen tension and immunologic response in the current era. Further understanding of these underlying mechanisms may facilitate optimization if the interventions prove effective, or understanding if they do not. Her we aim to study the effect of the EPOCH interventions on tissue oxygen tension and immunologic response.

Population

Patients enrolled in the Amsterdam UMC, location AMC who did not use immunosuppressants in the previous 90 days (e.g. glucocorticoids, cytostatic agents, antibodies, drugs acting on immunophilins, biologicals) will be approached for participation in additional measurements. This includes subcutaneous tissue oxygen tension measurement, mitochondrial tissue oxygen tension measurement and measurement of immunologic effects. As immunosuppressants blunt immunologic effects, potential differences, if any, will not be detectable in these patients. Therefore, patients using immunosuppressants are excluded for these additional measurements

Considering the substantial cost involved a small subset of the larger population will be measured based on a separate sample size calculation. Previous studies have shown the standard deviation in mean subcutaneous tissue oxygen tension is approximately 43 mm Hg.[5] Based on an alpha of 0.05, a beta of 0.2 (power of 0.8) and standard deviation in mean subcutaneous tissue oxygen tension of 43 mm Hg, 12 participants per arm are needed to show the previously reported 50 mmHg difference in subcutaneous tissue oxygen tension. Because the EPOCH study includes both laparoscopic and open surgery, and the two approaches have meaningful differences in hemodynamics and hemodynamic management, 24 consecutive eligible patients undergoing laparoscopic surgery, and 24 consecutive eligible patients undergoing open surgery will be enrolled until the final sample size is obtained for each. For the measurement of mitochondrial oxygen tension and immunologic effects, no reliable data is available to conduct a sample size estimation. Therefore, both will be measured as a pilot study investigating the 48 participants undergoing subcutaneous tissue oxygen tension measurement. Analysis of these pilot data will inform the need for further data collection.

Outcomes measures

- The difference in mean perioperative subcutaneous PtO_2 (mmHg) at the upper arm between the two treatment arms as measured by the Licox (Integra LifeSciences Corp, Plainsboro, New Jersey).

- The difference in mean perioperative mitochondrial PtO_2 (mmHg) in the epidermis, measured parasternal, between the two treatment arms as measured by the COMET (Photonics Healthcare B.V.).
- The difference in perioperative immune response between the two treatment arms as measured by neutrophil intracellular ROS generation, phagocytosis assays, plasma cytokine concentrations, plasma malondialdehyde concentration, leukocytic HLA-DRA mRNA expression, and ex vivo whole blood stimulation in blood samples withdrawn at five pre-specified timepoints.

Techniques

For subcutaneous tissue oxygen tension measurement, The Licox (Integra LifeSciences Corp, Plainsboro, New Jersey) system will be used. Licox continuously calculates tissue partial tension of oxygen through a Clark type polarographic oxygen electrode in a micro-catheter for oxygen measurements positioned in the tip of a flexible probe. The probe, with an outer diameter of 1.0mm will be inserted in the subcutaneous tissue with a 19-gauge venflon needle after local anaesthesia, and shortly before induction of general anaesthesia. Probes and instruments required for placement are sterile, single use and will be positioned maintaining sterile technique. The probe will be removed before discharge from the recovery room. For mitochondrial tissue oxygen measurement, the Cellular Oxygen METabolism (COMET) device (Photonics Healthcare B.V. (PH) will be used. The COMET is a non-invasive bedside monitoring system. After application of a dermatologic agent aminolevulinic acid (ALA) patch to the patient skin on the night before surgery, a sensor is applied to the skin of the thorax. The skin sensor creates a diverging excitation pulse allowing a superficial optical measurement of the oxygen in the mitochondria of the cells in the epidermis in a circular area of about 5 mm^2. Fluorescence light from the tissue is collected and transmitted via the detection fibre towards the detector system.[10-12] Blood samples (EDTA anticoagulated, LH anticoagulated, PAXgene) will be withdrawn at 5 pre-specified time points in a limited subset of the population (24 patients); along with routine preoperative blood sampling, after induction of anaesthesia but prior to incision, at the end of surgery, one hour postoperatively and at the first postoperative outpatient visit. Neutrophil ROS generation will be quantified using 2’,7’ dichlorofluorescein diacetate (DCFH-DA; Sigma, St Louis, MO, USA), as described by Perazzio et al.[13] Neutrophil phagocytosis will be measured using the pHrodo Red *E. coli* BioParticles Phagocytosis Kit for Flow Cytometry according to the manufacturer’s instructions (LifeTechnologies, Bleiswijk, the Netherlands). EDTA-anticoagulated blood will be centrifuged immediately after withdrawal (2000 g, 10 minutes, 4 °C). Plasma will be stored at − 80 °C until analysis. Concentrations of cytokines (including but not limited to TNFα, IL-6, IL-8, and IL-10) will be analysed batch-wise using a Luminex assay (detection range 3.2-10000 pg/mL) according to the manufacturer’s instructions (Milliplex, Millipore, Billerica, MA, USA). RNA will be isolated for HLA-DRA mRNA expression using the Paxgene Blood RNA kit (Qiagen, Valencia, CA, USA) and transcribed into cDNA using the iScript cDNA Synthesis kit (Bio-rad, Hercules, CA, USA). qPCR analysis was performed using TaqMan gene expression assays (Life Technologies, Paisley, UK) for the reference gene peptidylpropylisomerase B (PPIB) (#Hs00168719_m1) and HLA-DRA (#Hs00219575_m1) on a CFX96 Real-Time PCR Detection System (Bio-Rad, Hercules, CA, USA). Lipid peroxidation will be determined by measuring levels of malondialdehyde (MDA) in EDTA plasma using the Thiobarbituric Acid Reactive Substances (TBARS) Parameter Assay Kit (R&D Systems, Abingdon, UK) according to the manufacturer’s instructions. Leukocyte cytokine production capacity will be assessed by ex vivo stimulation of whole blood with Lipopolysacharide (LPS) ex vivo, as described by Kox et al.[14] Blood gas parameters will be analysed in LH anticoagulated blood using the Rapidlab 1200 Series (Bayer Healthcare LLC, Berlin, Germany) according to manufacturer’s instructions

For peer review only

References

1. Hunt TK. Surgical wound infections: an overview. Am J Med 1981;70:712-8.
2. Hopf HW, Hunt TK, West JM, et al. Wound tissue oxygen tension predicts the risk of wound infection in surgical patients. Arch Surg 1997;132:997-1004; discussion 5.
3. Govinda R, Kasuya Y, Bala E, et al. Early postoperative subcutaneous tissue oxygen predicts surgical site infection. Anesth Analg 2010;111:946-52.
4. Ives CL, Harrison DK, Stansby GS. Tissue oxygen saturation, measured by near-infrared spectroscopy, and its relationship to surgical-site infections. Br J Surg 2007;94:87-91.
5. Greif R, Akça O, Horn EP, Kurz A, Sessler DI. Supplemental perioperative oxygen to reduce the incidence of surgical-wound infection. N Engl J Med 2000;342:161-7.
6. Kurz A, Sessler DI, Lenhardt R. Perioperative normothermia to reduce the incidence of surgical-wound infection and shorten hospitalization. Study of Wound Infection and Temperature Group. N Engl J Med 1996;334:1209-15.
7. Dalfino L, Giglio MT, Puntillo F, Marucci M, Brienza N. Haemodynamic goal-directed therapy and postoperative infections: earlier is better. A systematic review and meta-analysis. Crit Care 2011;15:R154.
8. Qadan M, Battista C, Gardner SA, Anderson G, Akca O, Polk HC, Jr. Oxygen and surgical site infection: a study of underlying immunologic mechanisms. Anesthesiology 2010;113:369-77.
9. de Jonge S, Egger M, Latif A, et al. Effectiveness of 80% vs 30-35% fraction of inspired oxygen in patients undergoing surgery: an updated systematic review and meta-analysis. Br J Anaesth 2019;122:325-34.
10. Mik EG, Stap J, Sinaasappel M, et al. Mitochondrial PO2 measured by delayed fluorescence of endogenous protoporphyrin IX. Nat Methods 2006;3:939-45.
11. Mik EG, Johannes T, Zuurbier CJ, et al. In vivo mitochondrial oxygen tension measured by a delayed fluorescence lifetime technique. Biophys J 2008;95:3977-90.
12. Harms FA, de Boon WM, Balestra GM, et al. Oxygen-dependent delayed fluorescence measured in skin after topical application of 5-aminolevulinic acid. J Biophotonics 2011;4:731-9.
13. Perazzio SF, Salomao R, Silva NP, Andrade LE. Increased neutrophil oxidative burst metabolism in systemic lupus erythematosus. Lupus 2012;21:1543-51.
14. Kox M, Vrouwenvelder MQ, Pompe JC, van der Hoeven JG, Pickkers P, Hoedemaekers CW. The effects of brain injury on heart rate variability and the innate immune response in critically ill patients. J Neurotrauma 2012;29:747-55.

For peer review only

Academisch Medisch Centrum
Universiteit van Amsterdam

Patiënt informatie brief
Studie naar het effect van een geoptimaliseerd zorgprogramma rondom operaties op het voorkomen van wondinfecties.

Geachte mevrouw / mijnheer,
U bent door uw behandelend arts gevraagd om mee te doen aan een klinisch medisch wetenschappelijk onderzoek. In deze brief kunt u de informatie over dit onderzoek nog eens rustig nalezen, zodat u een weloverwogen beslissing kunt nemen. Wanneer u dat wilt, betrek dan ook uw eventuele partner of familie in de afweging. Voor algemene informatie verwijzen wij u naar de brochure "Medisch-wetenschappelijk onderzoek; Algemene informatie voor de proefpersoon". Wanneer u deze nog niet in bezit hebt, vraag dan uw arts ernaar.

Achtergrond
Wondinfecties na operaties behoren tot de meest voorkomende ziekenhuisinfecties. In Nederland ontwikkelt ongeveer 9% van alle patiënten die een buikoperatie ondergaat een wondinfectie. Wondinfecties variëren in ernst van een oppervlakkige ontsteking tot abcessen of bloedvergiftiging en geven altijd een verminderd operatie resultaat. Vaak moeten patiënten met een wondinfectie langer in het ziekenhuis blijven, opnieuw opgenomen worden of soms zelfs opnieuw geopereerd worden. Hierdoor zorgen wondinfecties voor onnodig veel pijn, ongemak, extra ziektelast en een aanzienlijke toename in de kosten. Wondinfecties zijn niet altijd te vermijden maar er zijn sterke aanwijzingen dat we onze huidige zorg rond operaties kunnen verbeteren om het risico naar beneden te brengen.

Doel van dit onderzoek
In Nederland is de standaard zorg om wondinfecties te voorkomen nu beschreven in de POWI (Post Operatieve Wond Infectie) bundel. Dit is een bundel van vier maatregelen die het risico op wondinfecties moeten verkleinen. Hierin staat dat patiënten op tijd antibiotica moeten krijgen, de deur tijdens de operatie niet te vaak open mag, u niet voor de operatie geschoren mag worden en dat u na de operatie niet te ver afgekoeld mag zijn. Er zijn sterke aanwijzingen dat andere, relatief eenvoudige, maatregelen tijdens de operatie het risico op wondinfecties terug kunnen brengen. Toch worden ze lang nog niet altijd toegepast. Nog niet alle artsen zijn overtuigd van het effect. Veel van deze maatregelen hebben effect op elkaar. Met deze studie willen we onderzoeken of de maatregelen als bundel elkaar versterken en een sterke vermindering geven op het aantal wondinfecties.

Deze bundel bestaat uit:

- Actieve verwarming rondom de operatie
    - Voor, tijdens en na de operatie krijgt de patiënt verwarmde dekens om te voorkomen dat hij of zij afkoelt

- Extra zuurstof tijdens de operatie
    - Tijdens de operatie geven we de patiënt lucht waar wat extra zuurstof in zit (80% i.p.v. 30%)

For peer review only - http://bmjopen.bmj.com/site/about/guidelines.xhtml

EPOCH2 — Effect of an Enhanced PeriOperative Care and Health Program

amc — Academisch Medisch Centrum, Universiteit van Amsterdam

- Bloedsuiker controle tijdens en kort na de operatie
  - Tijdens de operatie houden we het bloedsuikergehalte extra goed in de gaten en geven we de patiënt bloedsuiker verlagende medicijnen als dat nodig is. Na de operatie controleren we nog twee keer of het bloedsuikergehalte in orde is.

- Regulering van uw bloedsomloop op basis van een doelgericht protocol
  - We volgen een duidelijk doelgericht protocol waarin staat wanneer, en hoeveel vocht we toedienen tijdens de operatie om de bloedsomloop optimaal te houden.

- Gestandaardiseerde behandeling van de wond
  - Het wondgebied wordt volgens een gestandaardiseerde manier behandeld: Poetsen met alcohol en wondspoeling met jodium voor het dichtmaken van de wond.

Al deze maatregelen vinden voor het grootste deel plaats terwijl u onder narcose bent.

Daarnaast willen we onderzoeken welke rol patiënten kunnen spelen in het vaststellen van wondinfecties aan de hand van de resultaten van dit onderzoek.

Hoe wordt het onderzoek uitgevoerd?
Alle patiënten die een buikoperatie ondergaan zullen worden gevraagd mee te doen aan dit onderzoek. Om uit te zoeken of onze EPOCH bundel van maatregelen beter werkt dan de standaard zorg worden patiënten in twee groepen verdeeld. De eerste groep patiënten krijgt de standaard zorg zoals de dokters dat nu tijdens de operatie gewend zijn. De tweede groep krijgt de standaard zorg zoals de eerste groep, maar aangevuld met de EPOCH bundel aan extra maatregelen rondom de operatie. Verder gebeurt alles rondom de operatie zoals u dat met uw arts besproken heeft.
Als u besluit mee te doen met het onderzoek, wordt u ingedeeld in een van de twee groepen. Om de verdeling zo eerlijk mogelijk te houden, word deze bepaald door loting. Per dag wordt geloot in welke operatiekamers de standaard zorg wordt toegepast en in welke de EPOCH bundel daaraan toe wordt gevoegd.

Wat is er anders dan de reguliere behandeling die u krijgt?
Afhankelijk van de groep waarin u wordt geloot krijgt u de standaard zorg rondom operaties zoals we die nu gewend zijn, of diezelfde zorg aangevuld met de bundel aan extra maatregelen uit onze studie. Daarnaast wordt u gevraagd om een digitale foto te maken van de wond – indien u daar de technische mogelijkheden toe heeft – na 10 en na 30 dagen, en wanneer u in verband met zorgen om de wond contact hebt gehad met een arts. Met deze foto's kunnen we het proces van wondgenezing goed vervolgen. Op 30, 60 en 90 dagen na de behandeling wordt u gevraagd een aantal vragenlijsten in te vullen. Dit kan op uw smart Phone via de website of op papier. Deze vragenlijsten hebben betrekking op uw wondgenezing, klachten en welbevinden. Dit zal afhankelijk van de vragenlijst ongeveer 10 of 15 minuten per keer in beslag nemen. Bij de laatste vragenlijsten zitten twee extra vragenlijsten die nog eens 30 minuten in beslag nemen. In totaal 65 minuten verspreid over drie maanden. Hiervoor kunt u benaderd worden door de arts-onderzoeker, die hierdoor inzage heeft in uw persoonsgegevens.

For peer review only

For peer review only - http://bmjopen.bmj.com/site/about/guidelines.xhtml

Effect of an Enhanced PeriOperative Care and Health Program

Academisch Medisch Centrum
Universiteit van Amsterdam

Wat zijn mogelijke voor- en nadelen van deelname aan dit onderzoek?
Bij deelname aan dit onderzoek wordt u geloot tussen de standaard zorg, of de geoptimaliseerde zorg (EPOCH bundel). U heeft dus een 50/50 kans. Wij denken dat de geoptimaliseerde behandeling het risico op wondinfecties zou kunnen verlagen, maar dat moeten we nog bewijzen. Er is dus bij aanvang van dit onderzoek nog geen bewezen voordeel voor deelname aan dit onderzoek. Behalve 3 vingerprikjes voor de bloed glucose controle zijn van de extra maatregelen geen nadelen bekend. Wat wel een mogelijk nadeel van de studie is, is dat het invullen van de vragenlijsten wat extra tijd van u vergt. Verspreid over drie maanden is dat ongeveer een uur.

Wordt u geïnformeerd als er tussentijds voor u relevante informatie over de studie bekend wordt?
Het onderzoek zal zo nauwkeurig mogelijk en volgens een vooraf opgesteld plan verlopen. Maar de situatie kan veranderen. Als dat zo is, bespreken we dat direct met u. U beslist dan zelf of u met het onderzoek wilt stoppen of doorgaan.

Vrijwilligheid van deelname
Uw medewerking aan dit onderzoek is vrijwillig. Als u toestemming geeft om aan dit onderzoek mee te doen, heeft u te allen tijde de vrijheid om op die beslissing terug te komen. U hoeft hiervoor geen reden op te geven. Ook uw behandelend arts kan uw deelname aan het onderzoek stopzetten als deze vindt dat dit ten aanzien van uw gezondheid beter is. Uw arts bespreekt dit dan met u. Een beslissing om uw medewerking te beëindigen zal geen nadelige gevolgen hebben op de verdere behandeling en geen invloed hebben op de zorg en aandacht waarop u in ons ziekenhuis recht hebt.

Wanneer u afziet van deelname?
Als u geen toestemming geeft voor deelname aan het onderzoek, ondergaat u de standaard zorg rondom operaties zoals we dat nu gewend zijn conform de huidige richtlijnen.

Gebruik en bewaren van uw gegevens
Voor dit onderzoek worden uw persoonsgegevens verzameld, gebruikt en bewaard. Het gaat om gegevens zoals uw naam, adres, geboortedatum en om gegevens over uw gezondheid. Het verzamelen, gebruiken en bewaren van uw gegevens is nodig om de vragen die in dit onderzoek worden gesteld te kunnen beantwoorden en de resultaten te kunnen publiceren. Wij vragen voor het gebruik van uw gegevens uw toestemming.

Vertrouwelijkheid van uw gegevens
Om uw privacy te beschermen krijgen uw gegevens een code. Uw naam en andere gegevens die u direct kunnen identificeren worden daarbij weggelaten. Alleen met de sleutel van de code zijn gegevens tot u te herleiden. De sleutel van de code blijft veilig opgeborgen in de lokale onderzoeksinstelling. De gegevens bevatten alleen de code, maar niet uw naam of andere gegevens waarmee u kunt worden geïdentificeerd. Ook in rapporten en publicaties over het onderzoek zijn de gegevens niet tot u te herleiden.

For peer review only - http://bmjopen.bmj.com/site/about/guidelines.xhtml

Toegang tot uw gegevens voor controle
Sommige personen kunnen op de onderzoekslocatie toegang krijgen tot al uw gegevens. Ook tot de gegevens zonder code. Dit is nodig om te kunnen controleren of het onderzoek goed en betrouwbaar is uitgevoerd. Personen die ter controle inzage krijgen in uw gegevens zijn: de commissie die de veiligheid van het onderzoek in de gaten houdt, een controleur die voor het AMC als opdrachtgever werkt, nationale toezichthoudende autoriteiten, bijvoorbeeld, de Inspectie Gezondheidszorg. Zij houden uw gegevens geheim. Wij vragen u voor deze inzage toestemming te geven.

Bewaartermijn gegevens
Uw gegevens moeten 15 jaar worden bewaard op de onderzoekslocatie en 15 jaar bij de opdrachtgever (het AMC).
Het wordt bewaard om daarop in de loop van dit onderzoek nog nieuwe bepalingen te kunnen doen die te maken hebben met dit onderzoek.

Bewaren en gebruik van gegevens voor ander onderzoek
Uw gegevens kunnen na afloop van dit onderzoek ook nog van belang zijn voor ander wetenschappelijk onderzoek op het gebied van wondinfecties. Daarvoor zullen uw gegevens 15 jaar worden bewaard. U kunt op het toestemmingsformulier aangeven of u hier wel of niet mee instemt. Indien u hier niet mee instemt, kunt u gewoon deelnemen aan het huidige onderzoek.

Intrekken toestemming
U kunt uw toestemming voor gebruik van uw persoonsgegevens altijd weer intrekken. Dit geldt voor dit onderzoek en ook voor het bewaren en het gebruik voor het toekomstige onderzoek. De onderzoeksgegevens die zijn verzameld tot het moment dat u uw toestemming intrekt worden nog wel gebruikt in het onderzoek.

Meer informatie over uw rechten bij verwerking van gegevens
Voor algemene informatie over uw rechten bij verwerking van uw persoonsgegevens kunt u de website van de Autoriteit Persoonsgegevens raadplegen.
Bij vragen over uw rechten kunt u contact opnemen met de verantwoordelijke voor de verwerking van uw persoonsgegevens. Voor dit onderzoek is dat het AMC, zie contactgegevens verderop.
Bij vragen of klachten over de verwerking van uw persoonsgegevens raden we u aan eerst contact op te nemen met de onderzoek locatie. U kunt ook contact opnemen met de Functionaris voor de Gegevensbescherming van het AMC (zie contactgegevens) of de Autoriteit Persoonsgegevens.

Registratie van het onderzoek
Informatie over dit onderzoek is ook opgenomen in een overzicht van medisch-wetenschappelijke onderzoeken namelijk (www.trialregister.nl). Daarin zijn geen gegevens opgenomen die naar u herleidbaar zijn. Na het onderzoek kan de website een samenvatting van de resultaten van dit onderzoek tonen. U vindt dit onderzoek onder nummer NTR5694.

For peer review only - http://bmjopen.bmj.com/site/about/guidelines.xhtml

Bent u verzekerd wanneer u aan het onderzoek meedoet?
Aangezien aan deelname aan deze studie geen risico's verbonden zijn, heeft de Medisch Ethische Toetsingscommissie ontheffing verleend van de verplichting om voor de deelnemers een speciale schadeverzekering af te sluiten

Is er een vergoeding wanneer u besluit aan dit onderzoek mee te doen?
Er worden geen vergoedingen gedaan voor het meedoen aan dit onderzoek of voor uw reiskosten.

Hoe te handelen bij klachten?
Als u klachten heeft over het onderzoek, kunt u dit melden aan de onderzoeker. Wilt u dit liever niet, dan kunt u contact opnemen met het Bureau Patiëntvoorlichting van uw ziekenhuis. De patiëntenvoorlichting van het AMC Amsterdam is te bereiken op telefoonnummer: 020-5663355. Uw behandelend arts kan informatie geven over de patiënten service in uw ziekenhuis. Als (mogelijke) deelnemer aan dit onderzoek kunt u voor inlichtingen en advies ook terecht bij de onafhankelijke arts van de studie Dr. dr. T. Schepers chirurg AMC Amsterdam, te bereiken via telefoonnummer 020-5661895. Deze arts is niet bij de uitvoering van de studie betrokken, maar wel goed op de hoogte. Nadere uitleg kan uiteraard verstrekt worden door de behandelend arts, door ondergetekenden of door de onafhankelijk arts verbonden aan deze studie.

Tot slot
Mocht u na het lezen van deze brief of tijdens de onderzoeksperiode nog nadere informatie willen ontvangen of komen er nog vragen bij u op, dan kunt u altijd contact opnemen met een van de onderstaande contactpersonen of de website www.epochtrial.com bezoeken. Voor advies over deelname aan studie kunt u ook terecht bij een onafhankelijk arts die veel weet over de studie. Zijn gegevens vindt u hieronder.

Contactgegevens onderzoekers:

Studiecoördinator
Niels Wolfhagen
Academisch Medisch Centrum
Afdeling chirurgie
Postbus 22660 1100 DD Amsterdam
Telefoonnummer: 020-5666626
Email: epoch@amc.uva.nl

Hoofdonderzoeker
Prof. dr. M.A. Boermeester, Chirurg
Academisch Medisch Centrum
Afdeling chirurgie
Postbus 22660 1100 DD Amsterdam
Telefoonnummer: 020-5662666
Email: m.a.boermeester@amc.uva.nl

For peer review only - http://bmjopen.bmj.com/site/about/guidelines.xhtml

Effect of an Enhanced PeriOperative Care and Health Program

Academisch Medisch Centrum
Universiteit van Amsterdam

<u>Onafhankelijk arts</u>
dr. T. Schepers
Academisch Medisch Centrum, Afdeling chirurgie
Postbus 22660 1100 DD Amsterdam
020-5661895
Email: t.schepers@amc.uva.nl

<u>Functionaris voor de Gegevensbescherming van de instelling:</u>
Mw mr. J.B.M. Inge
Meibergdreef 9, kamer E2-212
1105AZ Amsterdam
Telefoonnummer: 020-5662015
E-mail: fg@amc.nl
Website:www.amc.nl

Wij hopen op uw medewerking.
Met vriendelijke groet namens de studiegroep,

Prof. M.A. Boermeester
Chirurg
Academisch Medisch Centrum

For peer review only
For peer review only - http://bmjopen.bmj.com/site/about/guidelines.xhtml

Effect of an Enhanced PeriOperative Care and Health Program

Academisch Medisch Centrum
Universiteit van Amsterdam

Toestemmingsformulier (Informed Consent)
Studie naar het effect van een geoptimaliseerd zorgprogramma rondom operaties.

Titel studie: EPOCH-trial: Effect of an Enhanced PeriOperative Care and Health protection program

Mijn specialist heeft mij gevraagd deel te nemen aan een studie naar een geoptimaliseerd zorgprogramma . Ik heb de bijgevoegde patiënt informatiebrief gelezen, het betreffende onderzoek besproken met de arts, ik heb de gelegenheid gehad om vragen te stellen en begrepen wat het onderzoek inhoudt.

Ik begrijp dat deelname aan dit onderzoek vrijwillig is en dat ik mij op elk moment, zonder opgave van reden, terug kan trekken uit dit onderzoek. Als ik dit doe, zal dit geen enkele invloed hebben op de verdere behandeling die ik van mijn arts(-en) zal ontvangen. Mijn huisarts wordt op de hoogte gebracht van mijn deelname aan dit onderzoek.

Ik geef toestemming tot inzage in mijn relevante medische gegevens door medewerkers van autoriteiten die belast zijn met de controle van medisch onderzoek. Ik begrijp dat de informatie die ik verstrek, zal worden verwerkt en geanalyseerd op de wijze die nodig is voor dit klinisch onderzoek en in overeenstemming met de Wet Bescherming Persoonsgegevens. Mijn gegevens worden geanonimiseerd en gekoppeld aan een studie nummer.

Ik verklaar deel te willen nemen aan dit onderzoek. Het ondertekenen van dit formulier heeft geen gevolgen voor mijn wettelijke rechten. Ik weet dat ik ook mag beslissen niet mee te doen. Mijn gegevens worden tot 15 jaar na afloop van het onderzoek bewaard.

- Ik geef **wel/geen toestemming*** om mij na dit onderzoek te benaderen voor vervolgonderzoek.
- **Ik geef wel/geen toestemming*** om mijn persoonsgegevens langer te bewaren en te gebruiken voor toekomstig onderzoek op het gebied van wondinfecties**.**
- Ik wil **wel/niet*** ingelicht worden over de eindresultaten van dit onderzoek.

Naam patiënt: ………………
Handtekening: ………………
Datum: … - … - ….

Ik heb de inhoud en het doel van het onderzoek uitgelegd aan bovenstaande patiënt. Hij/zij begrijpt de informatie en is in de gelegenheid gesteld vragen te stellen.

Naam arts: ………………
Naam ziekenhuis: ………………
Handtekening: ………………
Datum: … - … - …

For peer review only - http://bmjopen.bmj.com/site/about/guidelines.xhtml

Effect of an Enhanced PeriOperative Care and Health Program

Academisch Medisch Centrum
Universiteit van Amsterdam

For peer review only

For peer review only - http://bmjopen.bmj.com/site/about/guidelines.xhtml

Toestemmingsformulier (Informed Consent)
Studie naar het effect van een geoptimaliseerd zorgprogramma rondom operaties.

Titel studie: EPOCH-trial: Effect of an Enhanced PeriOperative Care and Health protection program

Mijn specialist heeft mij gevraagd deel te nemen aan een studie naar een geoptimaliseerd zorgprogramma . Ik heb de bijgevoegde patiënt informatiebrief gelezen, het betreffende onderzoek besproken met de arts, ik heb de gelegenheid gehad om vragen te stellen en begrepen wat het onderzoek inhoudt.

Ik begrijp dat deelname aan dit onderzoek vrijwillig is en dat ik mij op elk moment, zonder opgave van reden, terug kan trekken uit dit onderzoek. Als ik dit doe, zal dit geen enkele invloed hebben op de verdere behandeling die ik van mijn arts(-en) zal ontvangen. Mijn huisarts wordt op de hoogte gebracht van mijn deelname aan dit onderzoek.

Ik geef toestemming tot inzage in mijn relevante medische gegevens door medewerkers van autoriteiten die belast zijn met de controle van medisch onderzoek. Ik begrijp dat de informatie die ik verstrek, zal worden verwerkt en geanalyseerd op de wijze die nodig is voor dit klinisch onderzoek en in overeenstemming met de Wet Bescherming Persoonsgegevens. Mijn gegevens worden geanonimiseerd en gekoppeld aan een studie nummer.

Ik verklaar deel te willen nemen aan dit onderzoek. Het ondertekenen van dit formulier heeft geen gevolgen voor mijn wettelijke rechten. Ik weet dat ik ook mag beslissen niet mee te doen. Mijn gegevens worden tot 15 jaar na afloop van het onderzoek bewaard.

- Ik geef **wel/geen toestemming*** om mij na dit onderzoek te benaderen voor vervolgonderzoek.
- **Ik geef wel/geen toestemming*** om mijn persoonsgegevens langer te bewaren en te gebruiken voor toekomstig onderzoek op het gebied van wondinfecties**.**
- Ik wil **wel/niet*** ingelicht worden over de eindresultaten van dit onderzoek.

Naam patiënt: ………………
Handtekening: ………………
Datum: … - … - ….

Ik heb de inhoud en het doel van het onderzoek uitgelegd aan bovenstaande patiënt. Hij/zij begrijpt de informatie en is in de gelegenheid gesteld vragen te stellen.

Naam arts: ………………
Naam ziekenhuis: ………………
Handtekening: ………………
Datum: … - … - ….

9/9

Versie 8, 23-08-2019

For peer review only - http://bmjopen.bmj.com/site/about/guidelines.xhtml

SPIRIT

STANDARD PROTOCOL ITEMS: RECOMMENDATIONS FOR INTERVENTIONAL TRIALS

SPIRIT 2013 Checklist: Recommended items to address in a clinical trial protocol and related documents*

Section/item	Item No	Description	Report page no
**Administrative information**			
Title	1	Descriptive title identifying the study design, population, interventions, and, if applicable, trial acronym	1
Trial registration	2a	Trial identifier and registry name. If not yet registered, name of intended registry	3
	2b	All items from the World Health Organization Trial Registration Data Set	18
Protocol version	3	Date and version identifier	16
Funding	4	Sources and types of financial, material, and other support	20
Roles and responsibilities	5a	Names, affiliations, and roles of protocol contributors	1
	5b	Name and contact information for the trial sponsor	20
	5c	Role of study sponsor and funders, if any, in study design; collection, management, analysis, and interpretation of data; writing of the report; and the decision to submit the report for publication, including whether they will have ultimate authority over any of these activities	20

For peer review only

	5d	Composition, roles, and responsibilities of the coordinating centre, steering committee, endpoint adjudication committee, data management team, and other individuals or groups overseeing the trial, if applicable (see Item 21a for data monitoring committee)	20
**Introduction**			
Background and rationale	6a	Description of research question and justification for undertaking the trial, including summary of relevant studies (published and unpublished) examining benefits and harms for each intervention	5
	6b	Explanation for choice of comparators	5
Objectives	7	Specific objectives or hypotheses	6
Trial design	8	Description of trial design including type of trial (eg, parallel group, crossover, factorial, single group), allocation ratio, and framework (eg, superiority, equivalence, noninferiority, exploratory)	6
**Methods: Participants, interventions, and outcomes**			
Study setting	9	Description of study settings (eg, community clinic, academic hospital) and list of countries where data will be collected. Reference to where list of study sites can be obtained	7
Eligibility criteria	10	Inclusion and exclusion criteria for participants. If applicable, eligibility criteria for study centres and individuals who will perform the interventions (eg, surgeons, psychotherapists)	7
Interventions	11a	Interventions for each group with sufficient detail to allow replication, including how and when they will be administered	7, 8

For peer review only

	11b	Criteria for discontinuing or modifying allocated interventions for a given trial participant (eg, drug dose change in response to harms, participant request, or improving/worsening disease)	8
	11c	Strategies to improve adherence to intervention protocols, and any procedures for monitoring adherence (eg, drug tablet return, laboratory tests)	9
	11d	Relevant concomitant care and interventions that are permitted or prohibited during the trial	8
Outcomes	12	Primary, secondary, and other outcomes, including the specific measurement variable (eg, systolic blood pressure), analysis metric (eg, change from baseline, final value, time to event), method of aggregation (eg, median, proportion), and time point for each outcome. Explanation of the clinical relevance of chosen efficacy and harm outcomes is strongly recommended	10-11
Participant timeline	13	Time schedule of enrolment, interventions (including any run-ins and washouts), assessments, and visits for participants. A schematic diagram is highly recommended (see Figure)	11 + attachment figure 1
Sample size	14	Estimated number of participants needed to achieve study objectives and how it was determined, including clinical and statistical assumptions supporting any sample size calculations	12
Recruitment	15	Strategies for achieving adequate participant enrolment to reach target sample size	12
**Methods: Assignment of interventions (for controlled trials)**			
Allocation:			

For peer review only - http://bmjopen.bmj.com/site/about/guidelines.xhtml

Sequence generation	16a	Method of generating the allocation sequence (eg, computer-generated random numbers), and list of any factors for stratification. To reduce predictability of a random sequence, details of any planned restriction (eg, blocking) should be provided in a separate document that is unavailable to those who enrol participants or assign interventions	9
Allocation concealment mechanism	16b	Mechanism of implementing the allocation sequence (eg, central telephone; sequentially numbered, opaque, sealed envelopes), describing any steps to conceal the sequence until interventions are assigned	9
Implementation	16c	Who will generate the allocation sequence, who will enrol participants, and who will assign participants to interventions	9
Blinding (masking)	17a	Who will be blinded after assignment to interventions (eg, trial participants, care providers, outcome assessors, data analysts), and how	12
	17b	If blinded, circumstances under which unblinding is permissible, and procedure for revealing a participant's allocated intervention during the trial	
**Methods: Data collection, management, and analysis**			
Data collection methods	18a	Plans for assessment and collection of outcome, baseline, and other trial data, including any related processes to promote data quality (eg, duplicate measurements, training of assessors) and a description of study instruments (eg, questionnaires, laboratory tests) along with their reliability and validity, if known. Reference to where data collection forms can be found, if not in the protocol	13
	18b	Plans to promote participant retention and complete follow-up, including list of any outcome data to be collected for participants who discontinue or deviate from intervention protocols	13

Data management	19	Plans for data entry, coding, security, and storage, including any related processes to promote data quality (eg, double data entry; range checks for data values). Reference to where details of data management procedures can be found, if not in the protocol	13
Statistical methods	20a	Statistical methods for analysing primary and secondary outcomes. Reference to where other details of the statistical analysis plan can be found, if not in the protocol	13-14
	20b	Methods for any additional analyses (eg, subgroup and adjusted analyses)	14
	20c	Definition of analysis population relating to protocol non-adherence (eg, as randomised analysis), and any statistical methods to handle missing data (eg, multiple imputation)	14
**Methods: Monitoring**			
Data monitoring	21a	Composition of data monitoring committee (DMC); summary of its role and reporting structure; statement of whether it is independent from the sponsor and competing interests; and reference to where further details about its charter can be found, if not in the protocol. Alternatively, an explanation of why a DMC is not needed	14
	21b	Description of any interim analyses and stopping guidelines, including who will have access to these interim results and make the final decision to terminate the trial	15
Harms	22	Plans for collecting, assessing, reporting, and managing solicited and spontaneously reported adverse events and other unintended effects of trial interventions or trial conduct	15

Auditing	23	Frequency and procedures for auditing trial conduct, if any, and whether the process will be independent from investigators and the sponsor	15
**Ethics and dissemination**			
Research ethics approval	24	Plans for seeking research ethics committee/institutional review board (REC/IRB) approval	16
Protocol amendments	25	Plans for communicating important protocol modifications (eg, changes to eligibility criteria, outcomes, analyses) to relevant parties (eg, investigators, REC/IRBs, trial participants, trial registries, journals, regulators)	16
Consent or assent	26a	Who will obtain informed consent or assent from potential trial participants or authorised surrogates, and how (see Item 32)	9
	26b	Additional consent provisions for collection and use of participant data and biological specimens in ancillary studies, if applicable	Appendix 2
Confidentiality	27	How personal information about potential and enrolled participants will be collected, shared, and maintained in order to protect confidentiality before, during, and after the trial	17
Declaration of interests	28	Financial and other competing interests for principal investigators for the overall trial and each study site	21
Access to data	29	Statement of who will have access to the final trial dataset, and disclosure of contractual agreements that limit such access for investigators	17
Ancillary and post-trial care	30	Provisions, if any, for ancillary and post-trial care, and for compensation to those who suffer harm from trial participation	17

For peer review only

Dissemination polic	31a	Plans for investigators and sponsor to communicate trial results to participants, healthcare professionals, the public, and other relevant groups (eg, via publication, reporting in results databases, or other data sharing arrangements), including any publication restrictions	17
	31b	Authorship eligibility guidelines and any intended use of professional writers	17
	31c	Plans, if any, for granting public access to the full protocol, participant-level dataset, and statistical code	17
**Appendices**			
Informed consent materials	32	Model consent form and other related documentation given to participants and authorised surrogates	Appendix 2
Biological specimens	33	Plans for collection, laboratory evaluation, and storage of biological specimens for genetic or molecular analysis in the current trial and for future use in ancillary studies, if applicable	Appendix 1

*It is strongly recommended that this checklist be read in conjunction with the SPIRIT 2013 Explanation & Elaboration for important clarification on the items. Amendments to the protocol should be tracked and dated. The SPIRIT checklist is copyrighted by the SPIRIT Group under the Creative Commons "Attribution-NonCommercial-NoDerivs 3.0 Unported" license.

For peer review only
